# Sequestration of histidine kinases by non-cognate response regulators establishes a threshold level of stimulation for bacterial two-component signaling

**DOI:** 10.1038/s41467-023-40095-2

**Published:** 2023-07-25

**Authors:** Gaurav D. Sankhe, Rubesh Raja, Devendra Pratap Singh, Sneha Bheemireddy, Subinoy Rana, P. J. Athira, Narendra M. Dixit, Deepak Kumar Saini

**Affiliations:** 1 Centre for Biosystems Science and Engineering, Indian Institute of Science, Bengaluru, India; 2 Department of Chemical Engineering, Indian Institute of Science, Bengaluru, India; 3 Department of Developmental Biology and Genetics, Indian Institute of Science, Bengaluru, India; 4 Molecular Biophysics Unit, Indian Institute of Science, Bengaluru, India; 5 Materials Research Centre, Indian Institute of Science, Bengaluru, India

**Keywords:** Kinases, Bacteria, Regulatory networks

## Abstract

Bacterial two-component systems (TCSs) consist of a sensor histidine kinase (HK) that perceives a specific signal, and a cognate response regulator (RR) that modulates the expression of target genes. Positive autoregulation improves TCS sensitivity to stimuli, but may trigger disproportionately large responses to weak signals, compromising bacterial fitness. Here, we combine experiments and mathematical modelling to reveal a general design that prevents such disproportionate responses: phosphorylated HKs (HK~Ps) can be sequestered by non-cognate RRs. We study five TCSs of *Mycobacterium tuberculosis* and find, for all of them, non-cognate RRs that show higher affinity than cognate RRs for HK~Ps. Indeed, in vitro assays show that HK~Ps preferentially bind higher affinity non-cognate RRs and get sequestered. Mathematical modelling indicates that this sequestration would introduce a ‘threshold’ stimulus strength for eliciting responses, thereby preventing responses to weak signals. Finally, we construct tunable expression systems in *Mycobacterium bovis* BCG to show that higher affinity non-cognate RRs suppress responses in vivo.

## Abstract (web summary)

Bacterial two-component systems consist of a sensor histidine kinase (HK) that perceives a signal, and a cognate response regulator (RR) that modulates target gene expression. Here, the authors combine experiments and mathematical modelling to show that phosphorylated HKs can be sequestered by non-cognate RRs, which prevents responses to weak signals.

## Introduction

Two-component signaling systems (TCSs) form the primary apparati in bacteria for sensing and responding to extracellular cues^1^. Bacteria can have a few tens to a few hundred distinct TCSs. Each TCS comprises a sensor histidine kinase (HK), which is usually a transmembrane protein with a variable sensory domain and conserved catalytic domains, and a cognate cytosolic response regulator (RR) protein, which also contains a conserved catalytic domain and a variable output domain^1,2^. Stimulation by an extracellular cue through the sensory domain leads to HK autophosphorylation, followed by phosphotransfer from the HK to its cognate RR involving interaction between the conserved catalytic domains of both the proteins. The phosphorylation alters the DNA or, infrequently, RNA^3,4^ binding properties of the output domain of the RR, resulting in transcriptional changes or regulation of downstream genes and an adaptive response to the external stimulus^1,2^.

An important feature of the adaptive response, prevalent across TCSs, is positive autoregulation:^5^ The phosphorylated RR upregulates the expression of the corresponding HK and RR proteins. The increased HK and RR levels can increase the sensitivity of the TCS to the external stimulus and the magnitude of the adaptive response, respectively^6^. When the external stimulus is strong and persistent, positive autoregulation confers an advantage on the bacteria by expediting and amplifying the adaptive response. Indeed, positive autoregulation is a widely recognized biological design for amplifying responses, in addition to its effects on promoting step-like responses, hysteresis, and memory^7–10^. When the stimulus is weak or fleeting, however, positive autoregulation can be a disadvantage as it can lead to the mounting of a response that is disproportionately amplified and sustained given the stimulus. An important question that follows is how bacteria guard themselves against such disproportionate responses despite the presence of positive autoregulation.

Recent studies have presented designs that could create the requirement of a threshold level of stimulation before a response is mounted. For instance, in some plant TCSs and, more recently, in the TCS ArcAB of *E. coli*, multiple intermediate phosphotransfer events, resulting in a phosphorelay, have been identified in the phosphotransfer from HK to the cognate RR^11,12^. During the process, it is conceivable that a decay of a short-lived stimulus would cause the deactivation of HK and abort phosphotransfer. A minimum strength and/or duration of stimulation is thus necessary to complete the signal transduction event. Similarly, negative feedback could prevent the autoregulatory loop from getting activated until a threshold level of stimulation is realized^13,14^. Other designs include scaffolds and ligands that bind HK and dampen its activity^3,15,16^, small molecules that allosterically increase the phosphatase activity of HKs leading to rapid dephosphorylation of RRs following phosphotransfer^17–19^, and phosphate sinks that prematurely terminate signaling^20^. Indeed, some of these designs are being explored as routes to actively tune the detection thresholds of TCSs^18^. The designs explain the emergence of thresholds but imply that different designs exist in different settings for achieving the same goal. Given the ubiquitous nature of TCSs, we reasoned that a more widely prevalent motif for preventing disproportionate responses, both in strength and duration, may exist in bacterial systems.

Here, we identify a new design principle, involving the sequestration of autophosphorylated HKs by non-cognate RRs, that precludes an amplified response unless stimulation crosses a threshold level. We reasoned that if a phosphorylated HK has a higher binding affinity for a non-cognate RR than its cognate counterpart, then the HK is likely to be sequestered by the non-cognate RR. Only if the external stimulus leads to the accumulation of a sufficient amount of the phosphorylated HKs will the sequestration be overcome and phosphotransfer to the cognate RR occur. We unravel this design using mycobacterial TCSs, which we studied under in vitro and in vivo conditions, and using mathematical modeling. Our findings suggest that the design may be a widely prevalent mechanism for regulating TCS signaling.

## Results

Sequestration would occur if phosphorylated HKs were to bind preferentially to non-cognate RRs, so that their ability to transfer phosphoryl groups to their cognate RR partners is compromised. TCSs of *M. tuberculosis* have been shown in vitro to exhibit extensive crosstalk, where phosphorylated HKs not only bind but also transfer phosphoryl groups to non-cognate RRs^21,22^. They thus offered an excellent model system for us to test our hypothesis.

### The phosphorylated HK MtrB has a higher affinity for some non-cognate RRs than its cognate RR MtrA

**Fig. 1:**
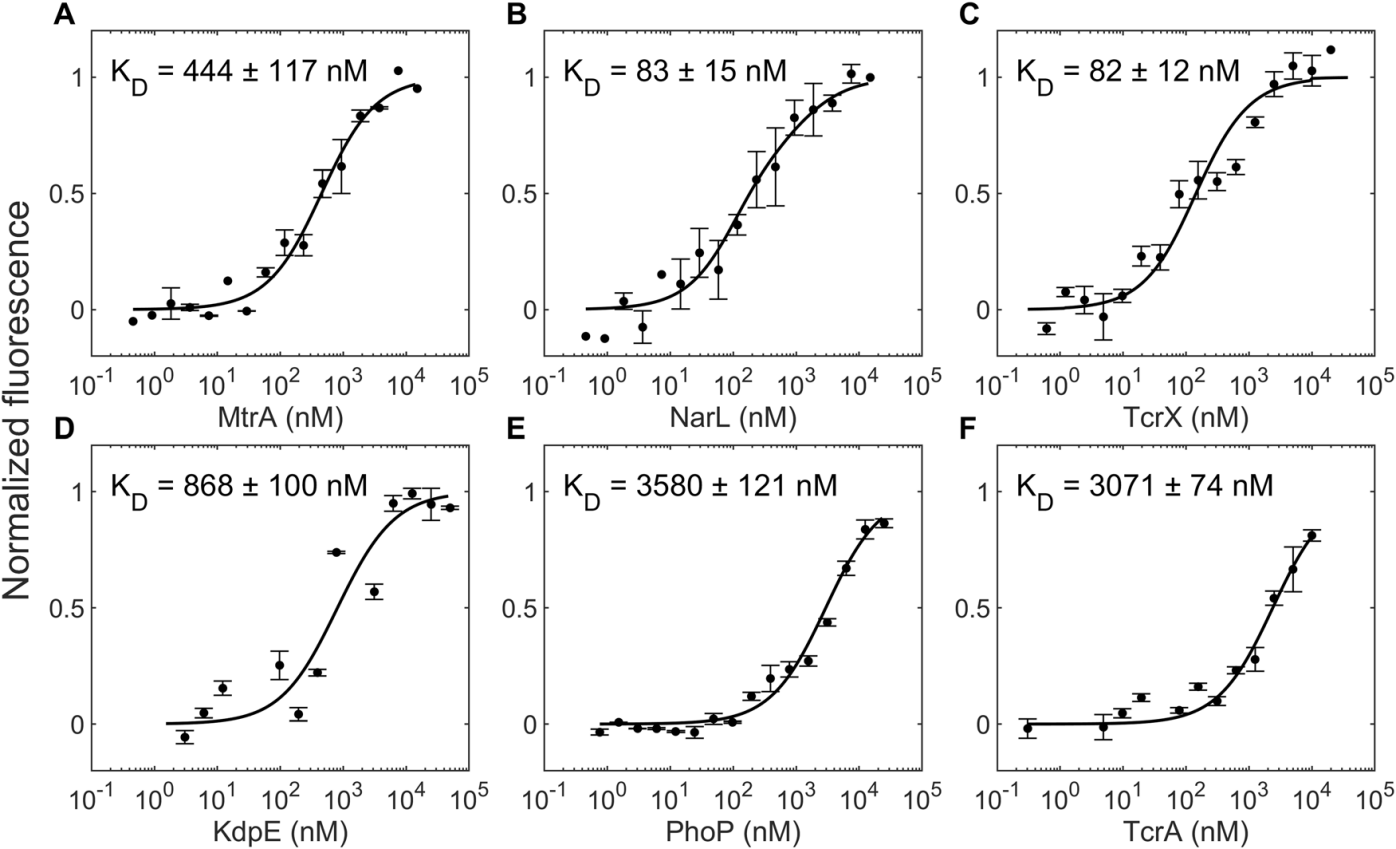
Binding affinities of phosphorylated MtrB for cognate and non-cognate RRs. Normalized fluorescence intensity obtained from microscale thermophoresis (see “Methods” section) of 50 nM of fluorescently tagged MtrB post autophosphorylation, P~MtrB-GFP, as a function of the concentration of the titrant RR (concentration range): **A** MtrA (0.45 nM to 15 μM), **B** NarL (0.46 nM to 15 μM), **C** TcrX (0.61 nM to 10 μM), **D** KdpE (3.1 nM to 50 μM), **E** PhoP (0.76 nM to 25 μM), and **F** TcrA (0.31 nM to 10 μM). The resulting K_D_ values are indicated. Curves are best-fits and symbols are mean ± S.E.M (*n* = 4 independent experiments for MtrB~P with TcrX and *n* = 3 independent experiments for the remaining plots).

We considered first the TCS MtrAB, which is among the most promiscuous of the TCSs of *M. tuberculosis*^21^ and is known to exhibit positive autoregulation^23^. We measured the binding affinities of the phosphorylated, GFP-tagged HK MtrB, reported to be active in previous studies^24^, for its cognate RR MtrA and several non-cognate RRs, using microscale thermophoresis (Methods). We found that the equilibrium dissociation constant, *K*_*D*_, of the phosphorylated MtrB, denoted MtrB~P, for MtrA was 444 ± 117 nM, whereas it was 83 ± 15 nM and 82 ± 12 nM, for the non-cognate RRs NarL and TcrX, respectively (Fig. 1). Remarkably, MtrB~P thus had a higher affinity for NarL and TcrX than for its cognate partner MtrA. For three other non-cognate RRs, namely KdpE, PhoP, and TcrA, the affinities were 868 ± 100 nM, 3580 ± 121 nM, and 3071 ± 74 nM, respectively, all lower than for MtrA. The order of affinities of MtrB~P for the different RRs tested was thus NarL~TcrX>*MtrA*>KdpE>TcrA>PhoP.

In the unphosphorylated state, the binding affinities of MtrB were consistently lower (Fig. S1). The *K*_*D*_ of GFP-tagged MtrB for MtrA was 936 ± 113 nM, indicating an over 2-fold weaker binding than its phosphorylated analog. Although the affinities were lower, NarL continued to display tighter binding to MtrB (*K*_*D*_ = 402 ± 58 nM) than MtrA. However, the above rank ordering was not conserved in the unphosphorylated state. For the other two non-cognate RRs we examined, namely, TcrX and PhoP, the affinities were 21715 ± 5022 nM and 18087 ± 2736 nM, respectively, so the rank ordering was NarL>*MtrA*>PhoP>TcrX (Fig. S1).

We recognized that our affinity measurements may be confounded by the presence of species combinations other than HK~P/RR, namely, HK~P/RR~P, HK/RR~P, and HK/RR, in our reaction mixture. To rule out these confounding effects, we repeated our experiments with phosphor-defective RR mutants. Specifically, we generated the mutants MtrA^D56N^ and NarL^D61N^, both capable of binding MtrB~P but incapable of accepting phosphoryl groups from it (see below). Thus, two of the four species complexes, namely those involving RR~P, would be eliminated from the reaction mixture. We found that the affinities of MtrB~P for MtrA and the mutant MtrA^D56N^ were similar; *K*_D_ = 444 ± 117 nM and 371 ± 130 nM, respectively (Fig. S2). Similarly, the affinities of MtrB~P for NarL and the mutant NarL^D61N^ were similar; K_D_ = 83 ± 15 nM and 78 ± 13 nM, respectively (Fig. S2). This implied that the presence of RR~P did not confound our estimates. Of the remaining two combinations, HK~P/RR and HK/RR, we inferred that our measurements were dominated by HK~P/RR affinities as follows: We independently measured the affinity of MtrB for MtrA and found it to be about 2-fold lower than that of MtrB~P for MtrA (*K*_D_ = 936 ± 113 nM vs. 444 ± 117 nM; see Fig. S1) implying that HK~P/RR complexes would outnumber HK/RR complexes. Further, the technique we used, microscale thermophoresis, measures the change in the thermophoretic movement of a fluorescently tagged species (change in fluorescence intensities) as a function of the change in the concentrations of bound complexes (subjected to temperature gradients)^25^. Given that the HK~P/RR combinations have the highest affinities among the combinations and that the concentrations of the HK~P/RR complexes are likely to be the highest, the measurements would be dominated by HK~P/RR binding, whose concentrations are likely to change the most when HK~P is titrated against RR at different concentrations. Our affinity measurements are thus expected to reflect HK~P/RR affinities. Finally, to ensure that the cognate and non-cognate RRs did not differentially affect the levels of HK~P in the reaction mix, which could confound inferences of affinity, we measured the levels of MtrB~P when incubated with either MtrA^D56N^ or NarL^D61N^ over timescales similar to those in the thermophoresis assays. The MtrB~P levels were unaffected by the RRs (Fig. S3). We concluded therefore that the various species combinations above did not affect our inferences of the affinities.

To further establish our findings, we repeated our measurements using two independent techniques. First, we used another solution phase interaction analysis technique, isothermal titration calorimetry (ITC), and found that the affinity of MtrB~P for NarL was nearly 3-fold higher than for MtrA (Table S1 and Fig. S4). Second, we used Biolayer interferometry (BLI), where biotinylated MtrB~P was immobilized on a streptavidin-coated surface and titrated against MtrA or NarL. Again, we found, remarkably, that MtrB~P had a 4.3-fold higher affinity for NarL than MtrA (Fig. S5). The numerical values of the affinities are expected to differ from those obtained with MST, given the different measurement modalities involved^25^. Yet, the similarity of the relative magnitudes obtained from 3 different techniques validates our finding that the affinity of HK~Ps for some non-cognate RRs can be higher than for their cognate partners.

To elucidate plausible origins of the observed binding affinities, we employed homology modeling of the kinase domain of MtrB bound to the receiver domain of MtrA or NarL and found that the MtrB:NarL complex was stronger (interaction energy of −40 kcal/mol) than the MtrB:MtrA complex (−15 kcal/mol), the difference attributable to a greater presence of ionic interactions in the MtrB:NarL interaction interface (Note S1, Table S2 and S3, and Figs. S6 and S7). As a control, we found that MtrB had hardly any interaction with the known non-binder RR PdtaR (interaction energy of +5 kcal/mol). Molecular modeling thus reinforces our finding and offers plausible insights into the tighter binding of NarL than MtrA with MtrB.

We examined next whether this preferential binding of phosphorylated HKs to non-cognate RRs was also seen with other TCSs of *M. tuberculosis*.

### Preferential binding of phosphorylated HKs to non-cognate RRs appears widely prevalent

**Fig. 2:**
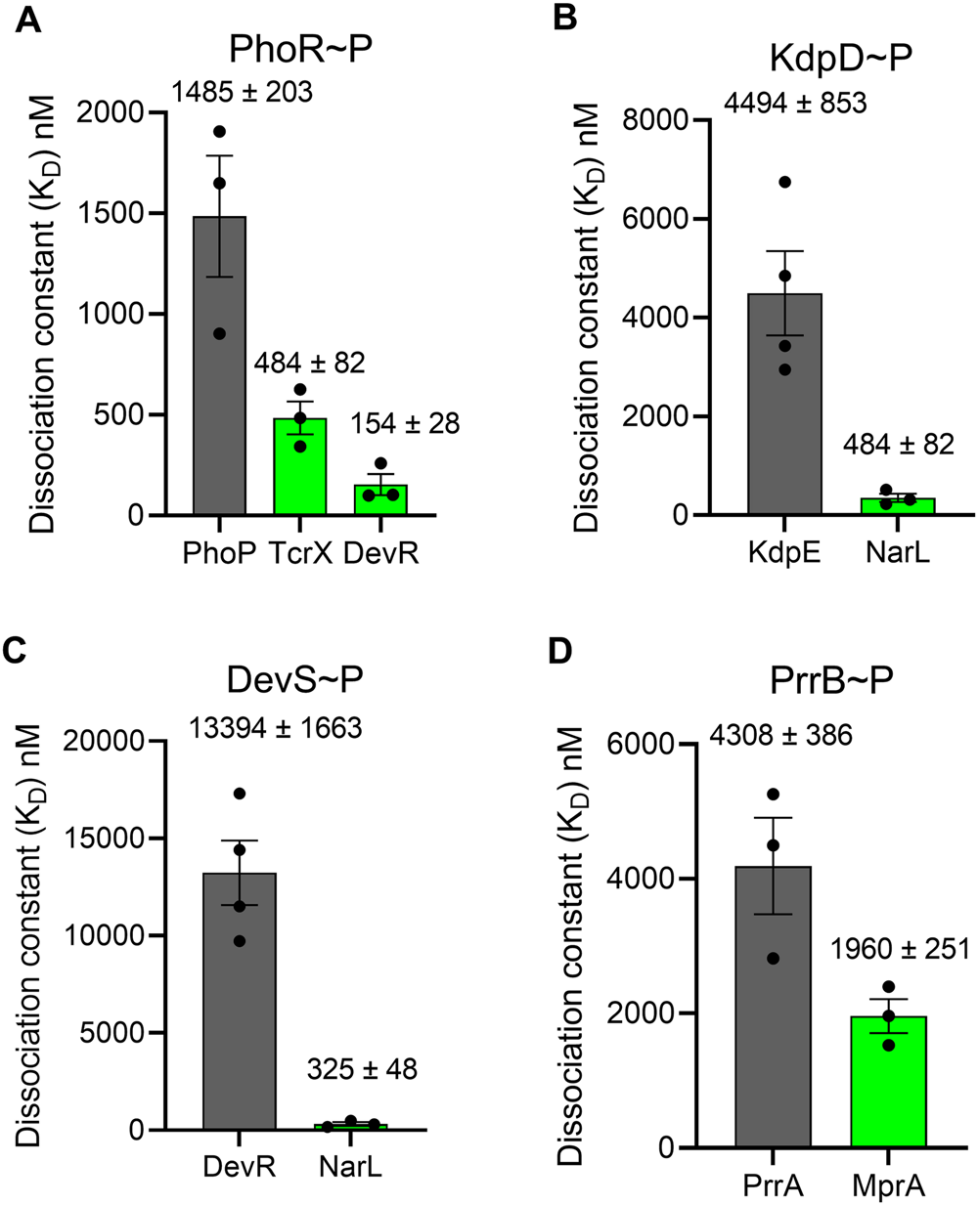
Binding affinities of several HKs for their cognate and non-cognate RRs. Affinities measured as in Fig. 1 for phosphorylated (**A**) PhoR, (**B**) KdpD, (**C**) DevS, and (**D**) PrrB, for their respective cognate (gray) and some non-cognate (green) RRs implicated in crosstalk with the HKs. The affinities as mean ± S.E.M. from at least three repeats are indicated. Detailed measurements leading to the affinity estimates are in Figs. S8 and S9, including for any non-cognate RRs with weaker affinities than the cognate ones. The error bars represent mean ± S.E.M (*n* = 4 independent experiments for KdpD~P with KdpE and DevS~P with DevR interaction, *n* = 3 independent experiments for remaining bar plots).

We measured the binding affinities of four other phosphorylated, GFP-tagged (MtrB and PhoR) or histidine tag labeled (KdpD, DevS, and PrrB) HKs of *M. tuberculosis* for their cognate as well as several non-cognate RRs, the latter implicated previously to be involved in crosstalk with the corresponding HKs^21^. Remarkably, we found that in every case there was at least one non-cognate RR for which the HK had a higher affinity than its cognate partner (Figs. 2, S8, and S9). The HK PhoR had a higher affinity for the non-cognate RRs TcrX and DevR (*K*_*D*_ = 484 ± 82 nM and 154 ± 28 nM, respectively) than its cognate partner PhoP (*K*_*D*_ = 1485 ± 203 nM) and another non-cognate RR TcrA (*K*_*D*_ = 1827 ± 163 nM); the HK KdpD had a higher affinity (*K*_*D*_ = 355 ± 44 nM) for NarL than its cognate partner KdpE (*K*_*D*_ = 4494 ± 853 nM); the HK DevS had a higher affinity (*K*_*D*_ = 325 ± 48 nM) for NarL than its cognate partner DevR (*K*_*D*_ = 13234 ± 1663 nM); and the HK PrrB had a higher affinity (*K*_*D*_ = 1960 ± 251 nM) for the RR MprA than its cognate partner PrrA (*K*_*D*_ = 4308 ± 386 nM).

We thus found that in every one of the five TCSs of *M. tuberculosis* we examined, including MtrAB, the phosphorylated HKs had a higher binding affinity for some non-cognate RR than their cognate partners. The affinities were at least 2-fold higher (as with PrrAB) but could be over 40-fold higher (as with DevRS) for the non-cognate RRs compared to the cognate partners. A consequence would be that the phosphorylated HKs bind preferentially to the higher affinity non-cognate RRs compared to their cognate partners. Thus, phosphorylated MtrB, for instance, would bind preferentially to NarL and TcrX over its cognate counterpart MtrA. Similarly, phosphorylated DevS would bind NarL in preference over DevR. Because phosphotransfer to non-cognate counterparts is typically inefficient^26,27^, this binding could amount to the sequestration of the HKs from their cognate RRs and the arrest of signal transduction through the cognate pathway. We examined next the impact of this preferential binding order on phosphotransfer and signal transduction. We focused on MtrAB.

### Sequestration of MtrB by the non-cognate RR NarL inhibits phosphotransfer to the cognate RR MtrA in vitro

**Fig. 3:**
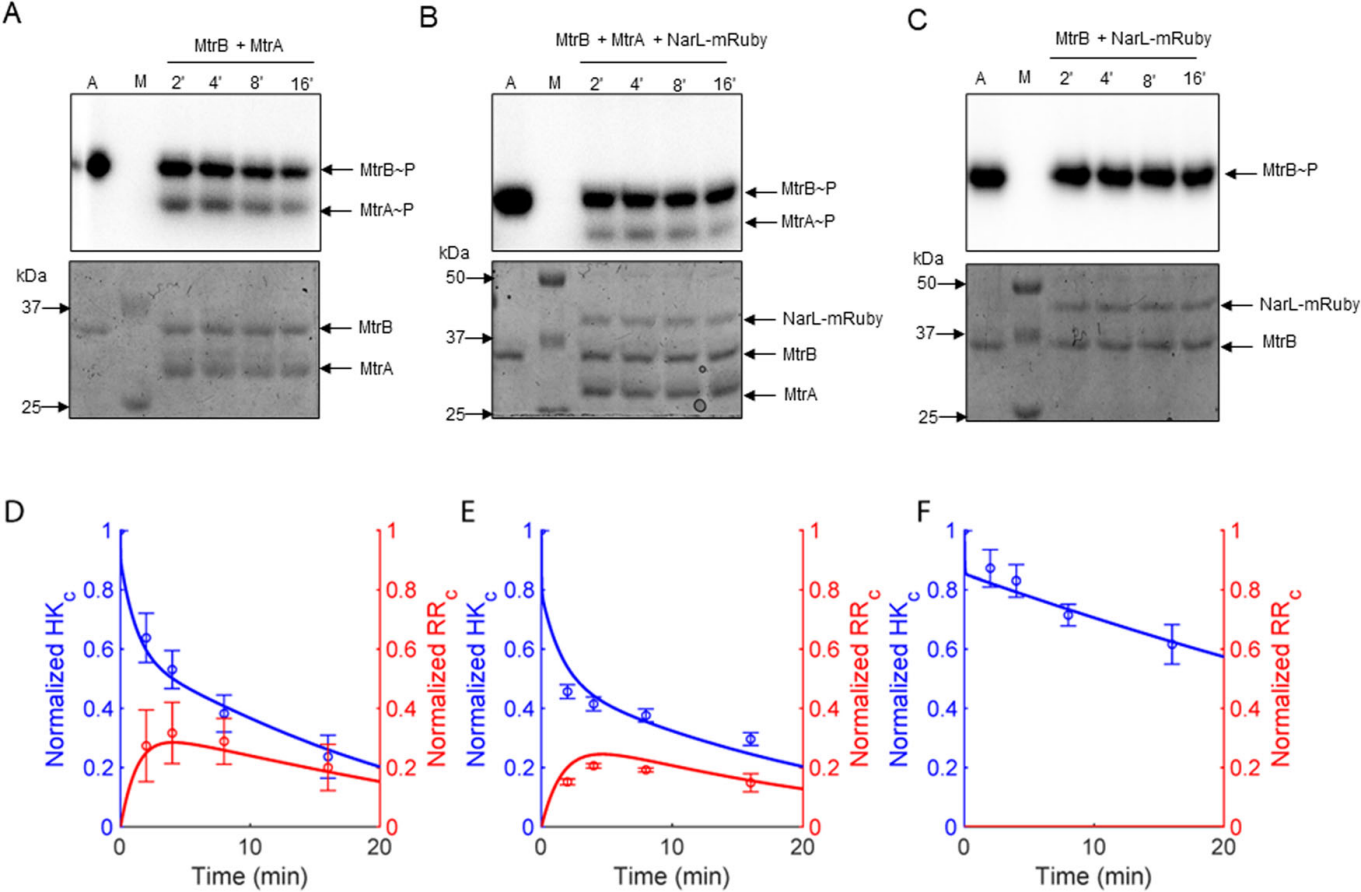
Phosphotransfer kinetics from MtrB to MtrA with and without sequestration in vitro. Time course assay of the phosphotransfer from the phosphorylated HK MtrB~P to the cognate RR MtrA in the (**A**) absence or (**B**) presence of the non-cognate RR NarL-mRuby. The concentrations used were 50 pmol for MtrB and NarL and 100 pmol for MtrA. **C** The same as (**B**) but in the absence of MtrA. M represents marker and A represents autophosphorylation control of MtrB~P. Top panels in **A**–**C** are autoradiograms and bottom panels are the corresponding Coomassie Brilliant Blue (CBB) stained gels. **D**–**F** Densitometric analysis of the time course assays in **A**–**C**, respectively, performed using autoradiograph band intensities normalized by the same band in the CBB stained gel. The autophosphorylation control was used to normalize the intensities of the individual bands. Blue symbols represent MtrB~P and red symbols MtrA~P. Lines represent best-fits of our model (“Methods” section). The error bars represent mean ± S.E.M (*n* = 3).

We co-incubated the phosphorylated HK MtrB with its cognate RR, MtrA, in the presence or absence of the higher affinity non-cognate RR NarL tagged with mRuby, termed NarL-mRuby. We measured the levels of phosphorylated MtrB, MtrB~P, and phosphorylated MtrA, MtrA~P, as functions of time to assess the extent of phosphostransfer. The RRs were both in 2-fold excess (100 pmol each) of the HK (50 pmol), so that phosphotransfer was not limited by the availability of RRs. We found that as the MtrB~P levels decreased, MtrA~P levels rose (Fig. 3A, B). In the presence of NarL, the latter rise was subdued. Whereas peak MtrA~P levels reached 30% (a.u.) without NarL, they remained at a significantly lower level of 20% with NarL (Fig. 3D, E; *P* = 0.011 using a one-tailed Student’s *t* test). In the absence of MtrA, the decline of MtrB~P was far lower; whereas MtrB~P levels declined to ~20% of their initial value in 20 min in the presence of MtrA, they remained at ~60% without MtrA (Fig. 3C, F), ruling out significant phosphotransfer from MtrB~P to NarL. In another control, we replaced NarL-mRuby with the RR PdtaR-RFP, which did not interact with MtrB, and found that peak MtrA~P levels reached 30% (Fig. S10), similar to that in the absence of non-cognate RR (*P* = 0.37, using a one-tailed Student’s *t* test), ruling out any non-specific effects of non-cognate RRs or their fluorescent tags on the phosphotransfer reaction.

To further understand the observed kinetics, we examined MtrB autophosphorylation in the absence of any RR, cognate or otherwise, and found negligible decline in MtrB~P levels over time (Fig. S11), suggesting that spontaneous dephosphorylation of MtrB~P, which is expected to be weak^28^ (see Table S4), may not explain the decline in MtrB~P levels seen above (Fig. 3C, F). This was also true when we coincubated MtrB with the mutants MtrA^D56N^ or NarL^D61N^, both incapable of receiving phosphoryl groups from HK~P (Fig. S11), indicating that unphosphorylated RR did not interfere with the autophosphorylation of HK. The minimal loss of MtrB~P in the presence of NarL alone (Fig. 3C, F) was thus likely due to weak phosphotransfer to NarL and possible dephosphorylation of MtrB~P in the presence of NarL. Importantly, the reduced MtrA~P levels in the presence of NarL were thus a consequence of the ‘sequestration’ of MtrB~P by NarL.

A consequence of the sequestration would be the suppression of signaling through the cognate pathway. It would imply that higher levels of stimulation of the cognate pathway would be required to trigger positive autoregulation and mount a significant cognate response. To establish this concretely and to elucidate the design principle underlying the sequestration by non-cognate RRs we observed, we constructed a mathematical model.

### Mathematical model of TCS signaling in the presence of non-cognate RR

**Fig. 4:**
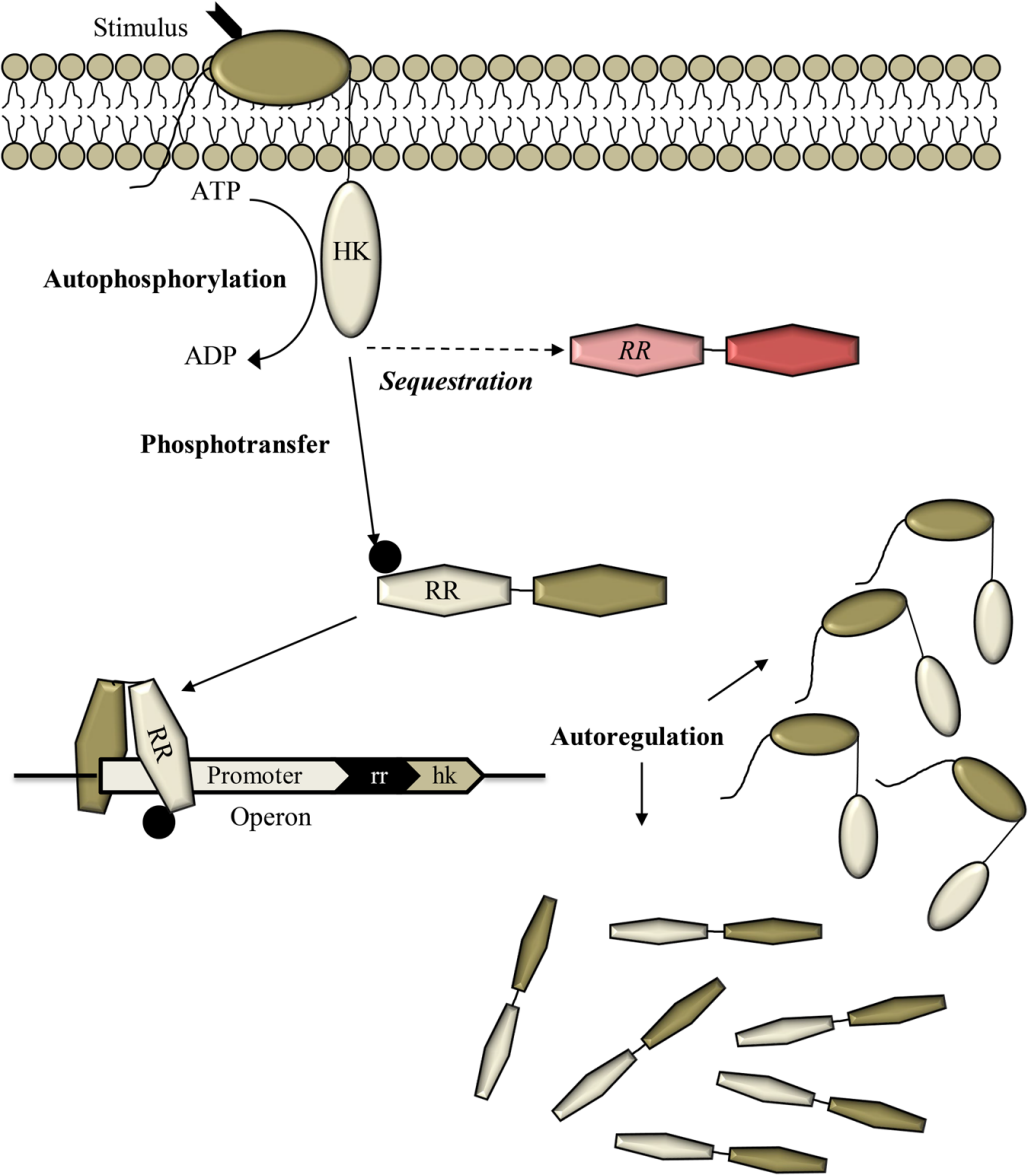
Schematic of the mathematical model. The model considers an extracellular stimulus triggering the autophosphorylation of HK and activating a TCS pathway. The phosphorylated HK can transfer the phosphoryl group to its cognate RR, which can bind DNA and trigger a response including the synthesis of the HK and RR proteins, marking positive autoregulation. The phosphorylated HK could bind non-cognate RRs (red) preferentially, when the latter have higher affinity for the HK than its cognate RR, resulting in HK sequestration and the suppression of cognate signaling. Only with a sufficiently strong stimulus does sufficient HK autophosphorylation result leading to cognate RR binding despite sequestration and the mounting of a response through the cognate pathway. Equations (1–19) list the reaction events in this model. The rate equations are in “Methods” section.

We developed a mathematical model of TCS signaling in vivo with positive autoregulation and the presence of a non-cognate RR (Fig. 4; “Methods” section). The model comprises the following events.

1 $$({{{{{\rm{Ligand}}}}}}\,{{{{{\rm{binding}}}}}})\,H{K}_{basal}+I \mathop{\rightleftharpoons }\limits_{{k}_{d}^{lig}}^{{{k}_{f}^{lig}}}HK$$

2 $$({{{{{\rm{ATP}}}}}}\,{{{{{\rm{binding}}}}}})\,HK+ATP \mathop{\rightleftharpoons }\limits_{{k}_{f}^{ATP}/{K}_{E}}^{{{k}_{f}^{ATP}}}HK-ATP$$

3 $$({{{{{\rm{Autophosphorylation}}}}}})\,HK-ATP\mathop{\longrightarrow }\limits^{{k}_{p}}H{K}^{\ast }+ADP$$

4 $$({{{{{\rm{Cognate}}}}}}\,{{{{{\rm{RR}}}}}}\,{{{{{\rm{binding}}}}}}\,HK)\,HK+R{R}_{c}\mathop{\rightleftharpoons }\limits_{{k}_{f}^{RR}\cdot {K}_{D}^{HK}}^{{{k}_{f}^{RR}}}HK-R{R}_{c}$$

5 $$({{{{{\rm{Cognate}}}}}}\,{{{{{\rm{RR}}}}}}\,{{{{{\rm{binding}}}}}}\,H{K}^{\ast })\,H{K}^{\ast }+R{R}_{c}\mathop{\rightleftharpoons }\limits_{{k}_{f}^{RR}\cdot {K}_{D}}^{{{k}_{f}^{RR}}}H{K}^{\ast }-R{R}_{c}$$

6 $$({{{{{\rm{Transition}}}}}}\,{{{{{\rm{complex}}}}}}\,{{{{{\rm{formation}}}}}})\,H{K}^{\ast }-R{R}_{c}\mathop{\rightleftharpoons }\limits_{{k}_{f}^{tc}\cdot {K}_{tc}}^{{{k}_{f}^{tc}}}HK-P-R{R}_{c}$$

7 $$({{{{{\rm{Phosphotransfer}}}}}})\,HK-P-R{R}_{c} \mathop{\rightleftharpoons }\limits_{{k}_{dp}^{RR}}^{{{k}_{p}^{RR}}}HK+R{R}_{c}^{\ast }$$

8 $$({{{{{\rm{Phosphatase}}}}}}\,{{{{{\rm{activity}}}}}})\,HK-P-R{R}_{c}\mathop{\longrightarrow }\limits^{{k}_{d}^{tc}}HK+R{R}_{c}+{P}_{i}$$

9 $$({{{{{\rm{Cognate}}}}}}\,{{{{{{\rm{RR}}}}}}}^{\ast }{{{{{\rm{binding}}}}}}\,H{K}_{basal})\,H{K}_{basal}+R{R}_{c}^{\ast }\mathop{\longrightarrow }\limits^{{k}_{dp}^{RR}}H{K}_{basal}-P-R{R}_{c}$$

10 $$({{{{{\rm{Phosphatase}}}}}}\,{{{{{\rm{activity}}}}}})\,H{K}_{basal}-P-R{R}_{c}\mathop{\longrightarrow }\limits^{{k}_{d}^{tc}}H{K}_{basal}+R{R}_{c}+{P}_{i}$$

11 $$({{{{{\rm{Noncognate}}}}}}\,{{{{{\rm{RR}}}}}}\,{{{{{\rm{binding}}}}}}\,HK)\,HK+R{R}_{nc} \mathop{\rightleftharpoons }\limits_{{k}_{f}^{RR-nc}\cdot {K}_{D-nc}^{HK}}^{{{k}_{f}^{RR-nc}}}HK-R{R}_{nc}$$

12 $$({{{{{\rm{Noncognate}}}}}}\,{{{{{\rm{RR}}}}}}\,{{{{{\rm{binding}}}}}}\,H{K}^{\ast })\,H{K}^{\ast }+R{R}_{nc} \mathop{\rightleftharpoons }\limits_{{k}_{f}^{RR-nc}\cdot {K}_{D-nc}}^{{{k}_{f}^{RR-nc}}}H{K}^{\ast }-R{R}_{nc}$$

13 $$({{{{{\rm{Transition}}}}}}\,{{{{{\rm{complex}}}}}}\,{{{{{\rm{formation}}}}}})\,H{K}^{\ast }-R{R}_{nc} \mathop{\rightleftharpoons }\limits_{{k}_{f}^{tc}\cdot {K}_{tc}}^{{k}_{f}^{tc}} HK-P-R{R}_{nc}$$

14 $$({{{{{\rm{Phosphatase}}}}}}\,{{{{{\rm{activity}}}}}})\,HK-P-R{R}_{nc}\mathop{\longrightarrow }\limits^{{k}_{d}^{tc-nc}}HK+R{R}_{nc}+{P}_{i}$$

15 $$({{{{{\rm{Dephosphorylation}}}}}})\,R{R}_{c}^{\ast }\mathop{\longrightarrow }\limits^{{k}_{d}^{RR}}R{R}_{c}+{P}_{i}$$

16 $$({{{{{\rm{Promoter}}}}}}\,{{{{{\rm{binding}}}}}})\,2R{R}_{c}^{\ast }+P \mathop{\rightleftharpoons }\limits_{{k}_{d}^{DNA}}^{{{k}_{f}^{DNA}}}{(R{R}_{c}^{\ast })}_{2}-P$$

17 $$({{{{{\rm{Basal}}}}}}\,{{{{{\rm{transcription}}}}}})\,P\mathop{\longrightarrow }\limits^{{k}_{btpn}}P+m$$

18 $$({{{{{\rm{Enhanced}}}}}}\,{{{{{\rm{transcription}}}}}})\,{(R{R}_{c}^{\ast })}_{2}-P\mathop{\longrightarrow }\limits^{{k}_{tpn}}{(R{R}_{c}^{\ast })}_{2}-P+m$$

19 $$({{{{{\rm{Translation}}}}}})\,m\mathop{\longrightarrow }\limits^{{k}_{tln}}m+\lambda H{K}_{basal}+R{R}_{c}$$

Briefly, the model considers HK in a basal state, denoted *HK*_*basal*_, which is activated by an input or stimulus,*I* (Eq. 1), to yield an activated HK, denoted *HK*. *HK* then binds ATP (Eq. 2) and gets autophosphorylated (Eq. 3). *HK* can bind the cognate RR, denoted *RR*_*c*_ (Eq. 4). The phosphorylated HK, denoted HK^*^, can bind *RR*_*c*_ (Eq. 5) and form a transition complex, $HK-P-R{R}_{c}$, poised for phosphotransfer (Eq. 6). The complex either effects phosphotransfer (Eq. 7), yielding phosphorylated RR, denoted $R{R}_{c}^{\ast }$, or dissociates with the loss of the phosphoryl group into inorganic phosphate, *P*_*i*_ (Eq. 8). *HK*_*basal*_ can also bind $R{R}_{c}^{\ast }$ forming a transition complex (Eq. 9) and exerting phosphatase activity (Eq. 10). We also allow the transition between *HK*_*basal*_ and *HK*, encoded in Eq. (1), to occur when *HK*_*basal*_ or *HK* are bound to other species. These events in Eqs. (1–10) are consistent with the prevalent understanding of HKs in TCSs^29^ where the basal state of the HK predominantly displays phosphatase activity, whereas ligand binding transforms it into a state prone to autophosphorylation and phosphotransfer to cognate RR.

We now consider events in the presence of a non-cognate RR. *HK* can bind the non-cognate RR, denoted *RR*_*nc*_ (Eq. 11). Importantly, HK^*^ can bind *RR*_*nc*_ (Eq. 12) and form a transition complex (Eq. 13). Unlike the complex above, the complex here is assumed not to be able to effect phosphotransfer to *RR*_*nc*_, resulting in the sequestration of HK^*^ by *RR*_*nc*_. Like the complex above, however, the transition complex may lead to the dephosphorylation of HK^*^ (Eq. 14).

Subsequent signal transduction is due to $R{R}_{c}^{\ast }$. $R{R}_{c}^{\ast }$ can get dephosphorylated spontaneously (Eq. 15) or dimerize and bind the promoter region P (Eq. 16). The basal transcription of downstream genes (Eq. 17) yielding mRNA, *m*, now happens at an enhanced rate (Eq. 18). Translation of the mRNA results in the production of the HK and RR_c_ proteins (Eq. 19), closing the positive autoregulation loop, and other response proteins.

We constructed rate expressions for the above events, which resulted in a set of coupled ordinary differential equations (“Methods” section). The parameters (rate constants) and their estimated values are listed in Table S4. Most parameters were set to values known from previous studies. We estimated the remaining parameters by fitting a reduced version of the model to the in vitro data above. We describe the latter fitting next.

### Mathematical model fits in vitro data

We recognized that the in vitro system has fewer events than those considered in the in vivo model above. In particular, the events post phosphotransfer to RR, including gene expression and autoregulation, do not occur in vitro. We therefore constructed models representative of the in vitro system and performed formal model selection (“Methods” section, Note S2, Table S5, and Fig. S12). The resulting model comprised Eqs. (2–8) and (11–15) above and offered good fits to data including time-courses of MtrB~P and MtrA~P with and without NarL and of MtrB~P levels in the control experiment without MtrA (“Methods” section; Fig. 3). The best-fit parameters estimated are in Table S4. The parameters all had reliable confidence intervals. Further, the estimated fraction of RR proteins that was active, 3.6% (CI: 2.5–4.7%; see Table S4), was consistent with the small fraction (~3–15%) of active TCS proteins in vitro observed in previous studies^26,30^. The fits reiterated the observation that sequestration by non-cognate RRs can suppress the phosphorylation of the cognate RR. With all the parameters thus identified, we applied the full model to examine how the sequestration would influence signal transduction via the cognate TCS pathway in vivo.

### Sequestration by non-cognate RRs establishes a threshold for cognate TCS signal transduction

**Fig. 5:**
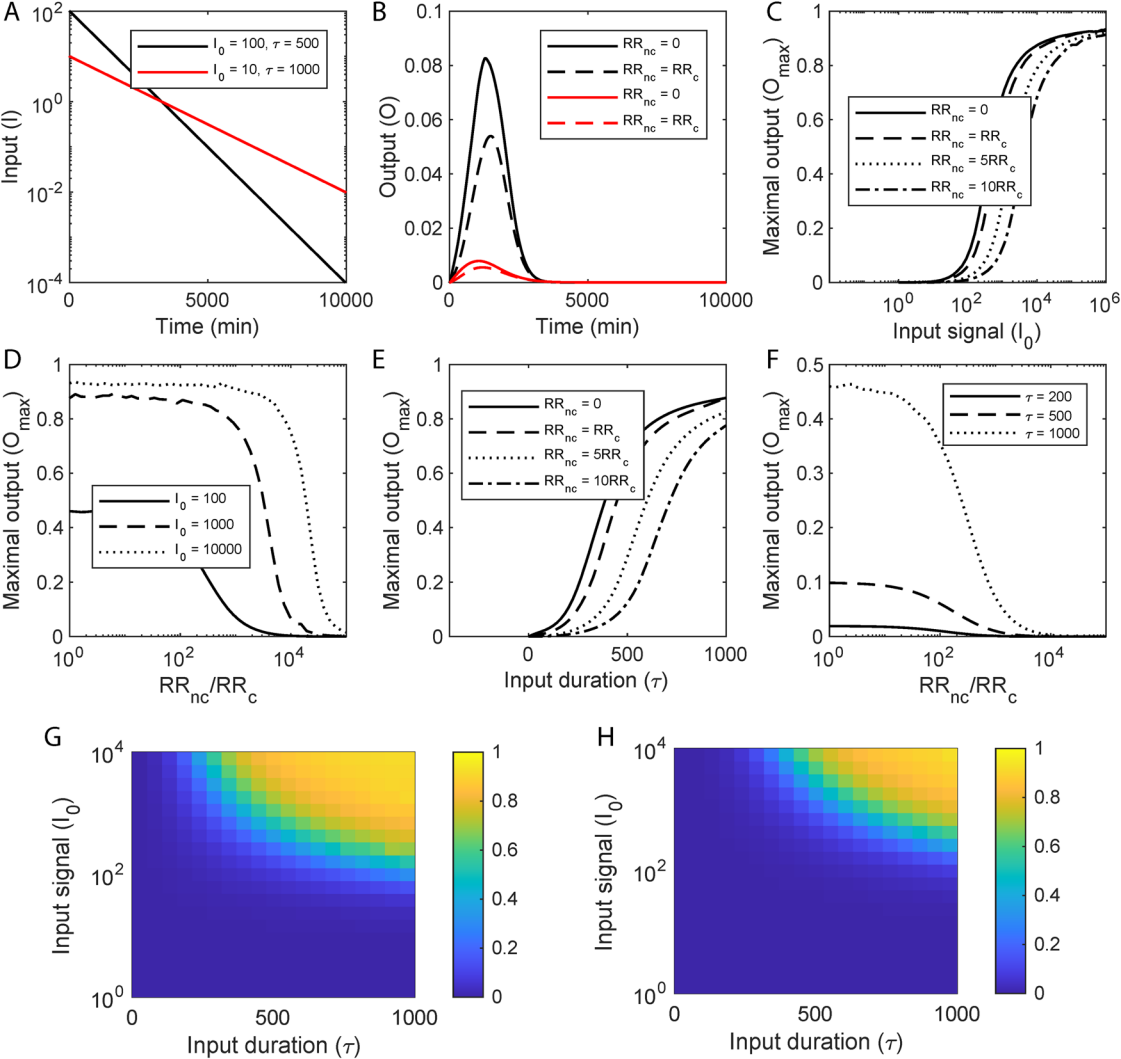
Model predictions of TCS signal transduction and the impact of sequestration. **A** Representative inputs, *I*, indicating strong but short-lived (black) and weak but extended (red) stimuli. **B** The corresponding outputs without (solid lines) and with (dashed lines) sequestration by a non-cognate RR. **C** The peak of the response (*O*_*max*_) as a function of the maximum input, *I*_0_, for different extents of sequestration, determined by the ratios of the non-cognate RRs, RR_nc_, to the cognate RR, RR_c_, indicated. **D** *O*_*max*_ as a function of the ratio RR_nc_/RR_c_ for different *I*_0_. **E** *O*_*max*_ as a function of the signal half-life, *τ*, for different values of RR_nc_/RR_c_. **F** *O*_*max*_ as a function of RR_nc_/RR_c_ for different values of *τ*. (*τ* is in minutes throughout.) Heatmaps showing *O*_*max*_ as functions of *I*_0_ and *τ* in the (**G**) absence or (**H**) presence of non-cognate RRs, indicating the threshold stimulation for response shifting to higher *I*_0_ and *τ* with sequestration. Corresponding calculations for the total response, *O*_*total*_, are in Fig. S13. Model predictions were obtained by solving Eqs. (20–47) (“Methods” section) using parameter values listed in Table S4.

We considered the input, *I*, to represent an environmental cue that rises sharply and then declines exponentially: *I* = *I*_0_exp(-*t/τ*). Here, *I*_0_ represents the strength of the signal and *τ* a measure of its duration. (We also considered square pulses, described below.) A large *I*_0_ and a small *τ* would represent a strong but fleeting stimulus, whereas a small *I*_0_ and a large *τ* would be a weak but lasting stimulus (Fig. 5A). We solved our model for a wide range of values of *I*_0_ and *τ*. We quantified the response, or output, as the fractional occupation of the promoter region by the cognate RR (“Methods” section).

For a given signal, the output, *O*, in the absence of sequestration (when no non-cognate RRs are present; i.e., when [RR_nc_] = 0) rose, attained a maximum, *O*_max_, and then declined to zero (Fig. 5B). (The output would stay elevated in response to a persistent signal (*τ*→∞); our focus here was on temporary and weak signals.) Increasing *I*_0_ or *τ* increased the maximum response, indicating that stronger or more sustained stimuli led to stronger responses. In the presence of sequestration ([RR_nc_]>0), however, the rise in the output was delayed, the maximum output was suppressed, and the output vanished sooner (Fig. 5B). In effect, sequestration by non-cognate RRs suppressed signal transduction through the cognate pathway.

We examined next how the maximum output, *O*_*max*_, and the cumulative output, *O*_*total*_, (area under the output-time curve) varied with *I*_0_ and *τ* in the absence and presence of sequestration. We found that *O*_*max*_ exhibited a sigmoidal dependence on *I*_0_, remaining low until a threshold level, *I*_*threshold*_, was crossed, rising sharply thereafter, and then reaching a saturation level, *O*_*sat*_, where further increases in *I*_0_ triggered marginal increases in *O*_*max*_ (Fig. 5C). Such nonlinear, sigmoidal responses are attributed to positive autoregulation^7^. With sequestration, we found that the sigmoid shifted to the right, i.e., to higher values of *I*_0_. Thus, in particular, *I*_*threshold*_ increased, indicating that a much larger level of stimulation was necessary for a significant response to be mounted. The shift increased with the level of RR_nc_, amounting to greater sequestration.

Conversely, for a given level of stimulation, *I*_0_, the maximum response, *O*_*max*_, exhibited an inverse sigmoidal dependence on RR_nc_ (Fig. 5D). As RR_nc_ increased from zero, *O*_*max*_ decreased gently until a critical RR_nc_ was crossed. At this point, *O*_*max*_ decreased sharply and reached minimal levels. The critical RR_nc_ was the value for which *I*_0_ became comparable to the threshold stimulation level, *I*_*threshold*_. Any RR_nc_ above this would amount to sequestration being strong enough that *I*_0_ would remain below *I*_*threshold*_, yielding a weak response. Thus, sequestration prevents the mounting of a strong response to short-lived stimuli.

A similar behavior was observed when *I*_0_ was kept constant and *τ* was varied. As *τ* increased, *O*_*max*_ rose in a sigmoidal manner, indicating the existence of a threshold duration, *τ*_*threshold*_, below which the output was weak and above which the output rose sharply to saturation, *O*_*sat*_ (Fig. 5E). As the level of sequestration increased, i.e., as RR_nc_ rose, *τ*_*threshold*_ increased, indicating that the stimulus had to last longer for a significant response to be mounted (Fig. 5E). Again, for a given *τ*, *O*_*max*_ exhibited an inverse sigmoidal dependence on RR_nc_, indicating the existence of a critical RR_nc_ at which *τ* became comparable to *τ*_*threshold*_ and above which *τ* was unable to elicit a significant response (Fig. 5F).

Heat maps comparing *O*_*max*_ as a function of *I*_0_ and *τ* with ([RR_nc_]>0) and without ([RR_nc_]=0) sequestration show that *O*_*max*_ was suppressed by sequestration when *I*_0_, *τ* or both were small (Fig. 5G, H). The same trend applied to *O*_*total*_ (Fig. S13), indicating that the results were robust to the choice of the output metric. We tested the robustness also to the nature of the input signals. We performed calculations using step inputs, instead of the exponentially decaying signals above, and found again that a threshold stimulation level for a significant response was introduced by sequestration (Fig. S14). Sequestration thus appeared as a design to prevent the mounting of a strong response despite positive autoregulation when the stimulation was weak or fleeting.

**Fig. 6:**
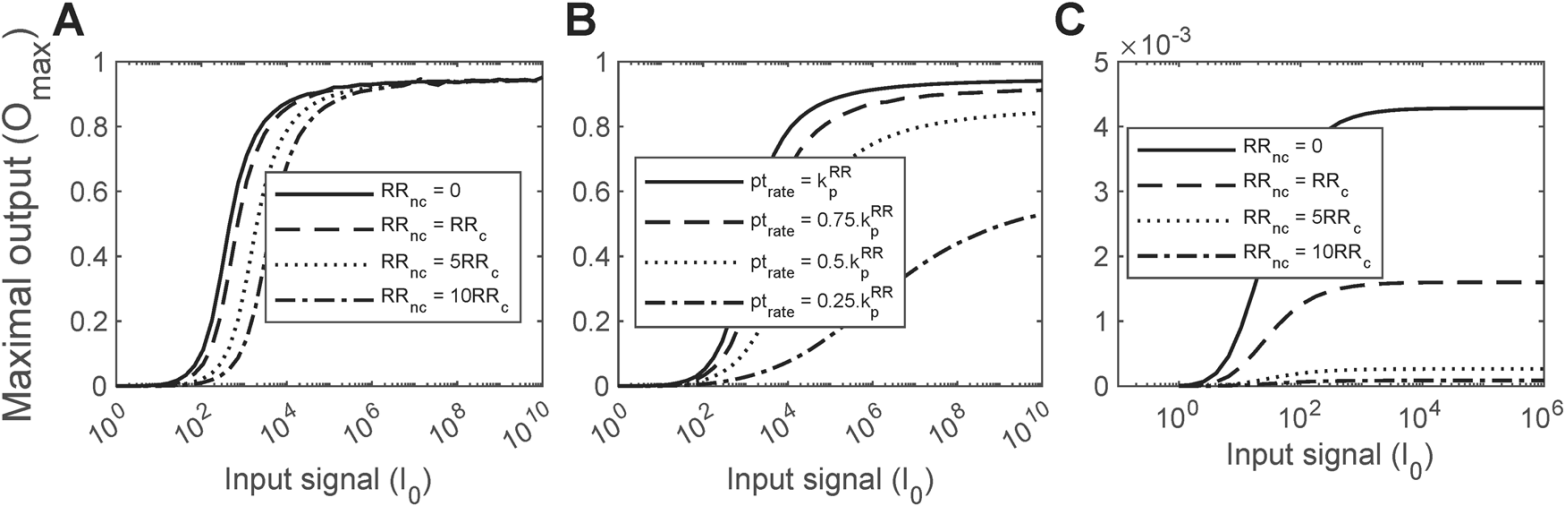
Advantage of sequestration over reduction in phospotransfer rate. **A** The peak of the response (*O*_*max*_) as a function of the maximum input, *I*_0_, for different extents of sequestration, determined by the ratios of the non-cognate RRs, RR_nc_, to the cognate RR, RR_c_, indicated. **B** *O*_*max*_ as a function of *I*_0_ for different phosphotransfer rates indicated. **C** *O*_*max*_ as a function of *I*_0_ for different ratios of the non-cognate RR_nc_/RR_c_ indicated, in the absence of positive autoregulation. Here, we also set protein degradation rates to zero, to eliminate the threshold introduced by the lack of proteins. Model predictions were obtained by solving Eqs. (20–47) (“Methods” section) using parameter values listed in Table S4.

Next, we applied our model to elucidate the advantage of sequestration over alternative designs. An obvious route to preventing the mounting of large responses is to reduce the phosphotransfer rate: The lower is the phosphotransfer rate, the weaker would be the response. We performed calculations in the absence of non-cognate RRs but with lower rates of phosphotransfer. Indeed, both lower phosphotransfer rates and non-cognate RRs gave rise to a threshold stimulation level for a robust response (Fig. 6A, B). However, with the lower phosphotransfer rate, the overall response was suppressed, even at high stimulation levels. With the non-cognate RRs, once the threshold was crossed, the response rose swiftly to the maximum level, allowing a more careful tuning of the response. This happened because once the threshold was crossed, the cognate signaling pathway took over due to autoregulation and dominated the response. With the lower phosphotransfer rate, the cognate pathway was permanently compromised.

Another route to preventing the mounting of large responses is sequestration but without positive autoregulation: greater is the extent of sequestration, greater would be the suppression of the cognate response. We therefore performed calculations with sequestration in the absence of positive regulation. Now, sequestration had an effect similar to that of lower phosphotransfer rates but stronger in magnitude, attenuating the response substantially even at high stimulation levels. (Fig. 6C). Thus, sequestration together with positive autoregulation offers the necessary design to prevent responses to weak or fleeting stimuli and mount robust responses to strong and lasting stimuli. This advantage of sequestration with positive autoregulation may have led to its evolutionary selection, explaining its prevalence in the TCSs we studied.

With this insight from mathematical modeling, together with evidence from our in vitro experiments, we examined whether this design was evident in vivo using our experimental models.

### Non-cognate RR suppresses TCS signal transduction in vivo

**Fig. 7:**
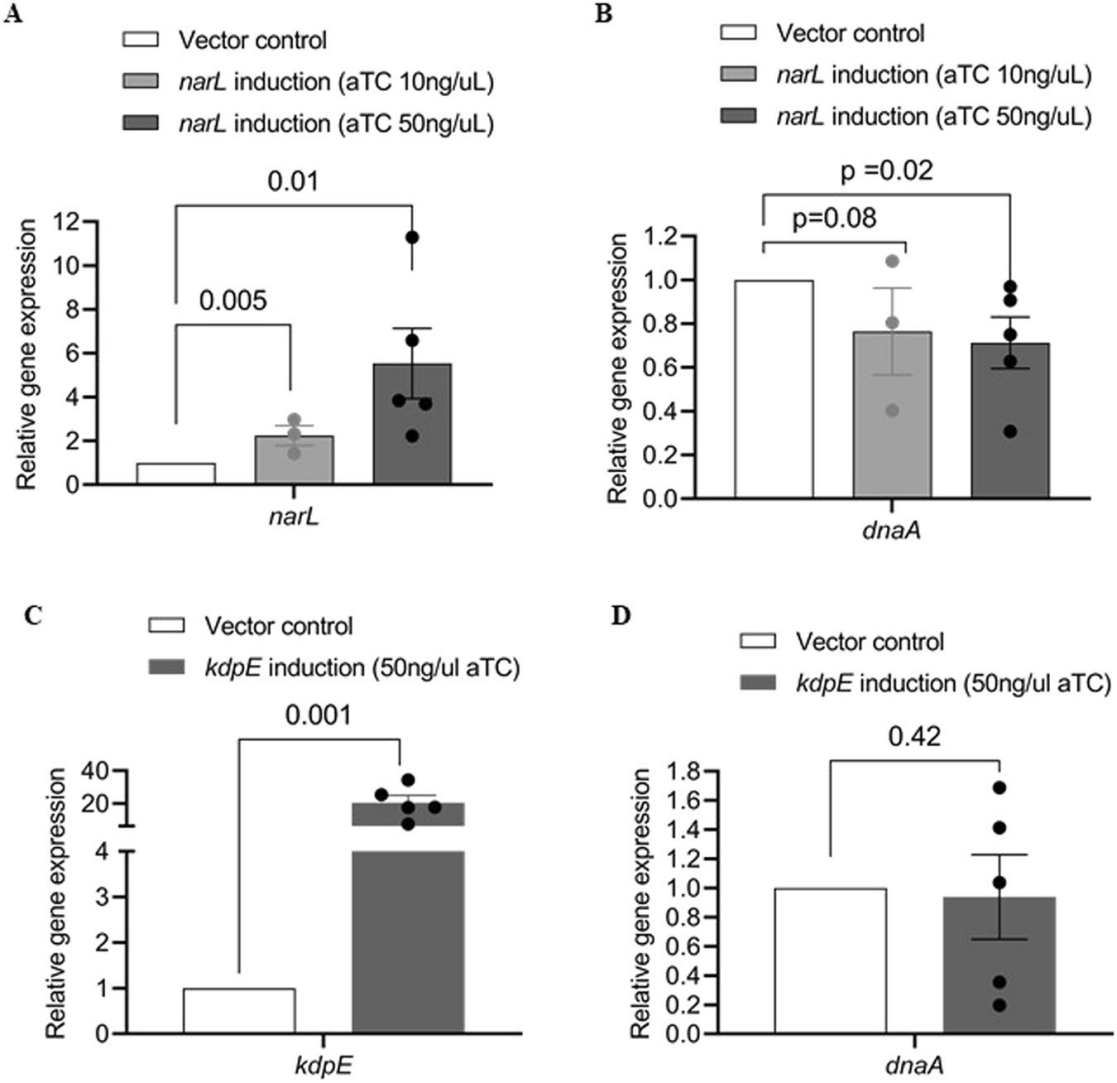
Effect of HK sequestration on the cognate TCS response in vivo. Effect of MtrB sequestration by two non-cognate RRs, a strong binder NarL and a weak binder KdpE examined by monitoring expression changes for MtrA-specific target *dnaA* in vivo. Overexpression of (**A**) *narL* in *M. bovis* BCG strain under tetracycline-inducible promoter using 10 ng/ml or 50 ng/ml aTC at OD_600_ ~ 0.5 for 8 h and (**B**) the corresponding expression level of *dnaA*. Analogous experiments with the induction of (**C**) *kdpE* using 50 ng/ml aTC at OD_600_ ~ 0.5 for 8 h and (**D**) the associated *dnaA* expression. Gene expression was normalized to the expression levels of 16 s rRNA, followed by the expression levels of the respective genes in the strain carrying only the vector pTic6 (vector control). Data represent *n* ≥ 3 biologically independent experiments. *P* values evaluated using one-tailed Student’s *t* test).

Our in vitro experiments above demonstrated the sequestration of MtrB~P by NarL. We examined the effect of this sequestration in vivo using *Mycobacterium bovis* BCG, a bovine-pathogenic surrogate of *M. tuberculosis*. The stimulus for the MtrAB system is unknown^31^. The system is known to be active during cell proliferation and regulates the expression of the downstream gene *dnaA*^32^. Because the stimulus is unknown, tuning it to define the threshold stimulus for responses was not possible. Our model predicted, however, that the extent of sequestration worked as a surrogate for the stimulus level/duration: increasing the extent of sequestration at a given stimulus level and duration was equivalent to decreasing the stimulus level or duration in the absence of sequestration (see Fig. 3C, E). Here, we therefore tested our design by altering the sequestration level. The expression level of NarL, a higher affinity non-cognate RR for MtrB, could be tuned, enabling control of the level of sequestration. We increased the level of NarL using the anhydrotetracycline (aTC) tunable expression system (pTIC6)^33^ and measured the changes in the transcript level of the gene *dnaA* as a function of NarL expression. We used two different levels of aTC, 10 ng/μl and 50 ng/μl, which resulted in a dose-dependent increase in NarL expression to ~2-fold and ~5.5-fold, respectively, above vector control (*p* = 0.005 and 0.001 using a one-tailed Student’s *t* test) (Fig. 7A). We simultaneously measured the *dnaA* transcript levels and found a significant decrease by ~25% and ~30%, respectively, in the two cases (*p* = 0.08 and 0.02 using a one-tailed Student’s *t* test) (Fig. 7B). As a control, we tuned the expression of another non-cognate RR KdpE shown to be a weak binder of phosphorylated MtrB in vitro. While the same aTC level (50 ng/μl) triggered a significant increase in kdpE expression, ~19-fold above vector control (*p* = 0.001, Fig. 7C), the expression of *dnaA* remained unaffected (*p* = 0.42, Fig. 7D). The reduction in *dnaA* levels due to upregulation of NarL was consistent with our model predictions made using parameter values representative of the in vivo scenario (Fig. S15). This marked, dose-dependent reduction in the level of *dnaA* due to the upregulation of the non-cognate RR, NarL, thus indicates the presence of the sequestration motif in vivo. Sequestration of phosphorylated HK by non-cognate RRs thus appears to be a design to prevent disproportionately amplified responses to weak or short-lived stimuli.

## Discussion

Our study reveals a new design principle underlying the regulation of bacterial two-component signaling (TCS) systems. Positive autoregulation of TCSs, where a TCS upregulates its own proteins in response to stimulation, is a widely prevalent feature that aids signal amplification and the mounting of a strong and lasting response to stimuli^10^. A downside of positive autoregulation, however, is that a disproportionate response may be triggered to weak or fleeting stimuli, which may cost resources and hence reduce bacterial fitness. How bacteria overcome this limitation has been puzzling. Here, we unraveled a mechanism, sequestration of HKs by non-cognate RRs, that offers an answer. We found with every one of the five mycobacterial TCSs we studied that HKs bind to at least one non-cognate RR more tightly than their cognate counterparts. The tighter-binding non-cognate RR thus sequesters the HK until a strong enough stimulus results in sufficient HK autophosphorylation that a ‘leak’ to the cognate RR ensues. Positive autoregulation then amplifies the HK and cognate RR protein levels, allowing significant signaling through the cognate pathway and the mounting of a proportionate response. We demonstrated this design principle using mathematical modeling as well as in vitro and in vivo experiments on mycobacterial TCSs.

The costs and benefits of positive autoregulation have been investigated extensively because of their importance to the regulation and control of biological processes in diverse settings^7,9,34–36^. Among the costs identified has been the delay in the response to a stimulus arising from the need to build up a sufficient amount of the entities involved before positive autoregulation can begin to amplify them^34–36^. When the positive autoregulatory network is made more sensitive, it allows a gain in speed, but could lead to excessive amplification of the long-term response, resulting in a substantial fitness cost. A coupled negative feedback loop has been proposed recently as a way to ensure speed in the response without the excessive long-term protein production^35^. The latter design seems appropriate when stimuli are strong and lasting, with the sensitive positive autoregulation ensuring speed and the coupled negative feedback preventing uncontrolled amplification. When the stimulus is weak or fleeting, however, for which a response may not be warranted, let alone a speedy one, the sensitive positive autoregulation may still initiate a response and amplify it, introducing a fitness cost. In the context of bacterial TCSs, we showed here that sequestration of HKs by non-cognate RRs introduces a threshold level of stimulation for a response to be mounted and prevents such unnecessarily speedy and amplified responses. Thus, while the negative regulation controls the response in the late stages, the sequestration we identified controls it in the early stages of stimulation. Together, the two present a more complete regulatory design of bacterial TCSs under positive autoregulation.

We examined alternative, simpler designs that could potentially help prevent disproportionate responses to weak signals but found them wanting in comparison to sequestration. For instance, lowering of the phosphotransfer rate, which could be achieved via mutations in the relevant HK/RR domains, does induce a threshold stimulus level for a response, but results in a subdued response even when the stimulus is strong, leaving the TCS compromised. Sequestration alone, *i.e*., in the absence of positive autoregulation, also leads to a similar global reduction in the cognate response and leaves the TCS compromised. An implication is that TCSs not under positive autoregulation may not favor sequestration for preventing responses to weak stimuli, which future studies may test.

We distinguish sequestration from sinks, the latter also argued to give rise to thresholds^20,24^. If non-cognate RR binding were to strip the HK of its phosphoryl group, then the non-cognate RR would act as a sink. It would become available for repeating the act with the next phosphorylated HK, creating a perpetual drain, or a sink, for phosphoryl groups. It is apparent that a sink would place a much harsher demand on the signal for eliciting a response because it would eliminate the possibility of signaling from any HK that it would bind. Sequestration, on the other hand, is likely to exert gentler control. It suppresses the signal temporarily and allows the HK to dissociate from the non-cognate RR with its phosphoryl group and retain its ability to trigger signaling through the cognate pathway. The circumstances that confer an evolutionary advantage upon sequestration over sinks remain to be elucidated.

We also distinguish between the threshold introduced by positive autoregulation alone from that due to sequestration. As mentioned above, the need to build up sufficient amounts of HK and RR proteins before positive autoregulation can begin to amplify them^34–36^ can introduce a threshold stimulus; a stimulus level that leads to HK and RR activation at rates lower than their degradation rates would preclude the necessary build up. The resulting threshold is thus largely determined by intrinsic protein degradation rates and may not be amenable to tuning. Sequestration, in contrast, introduces a threshold based on the levels/affinities of non-cognate RRs, which could be tuned. In a recent study, crosstalk between TCSs has been argued to be evolutionarily advantageous in programmed environments, where signals arise in predefined sequences, allowing the crosstalk to prime bacteria to upcoming signals^37^. In such programmed environments, one could imagine sequestration as a handle to tune responses to sequential signals: the signal stimulating a TCS may result in the upregulation of its RRs that may in turn act as sequesters of TCSs responsive to all but the subsequent signals, allowing a focusing of the bacterial response. The extent of upregulation of the RRs may tune the thresholds for the other TCSs. Future studies may evaluate these possibilities, via both modeling and experiments.

Mycobacterial TCSs have been found to engage in extensive cross-talk, where HKs transfer phosphoryl groups to non-cognate RRs^21^. A pre-requisite for this cross-talk is the binding of phosphorylated HKs with non-cognate RRs. The binding affinities of these interactions, however, had not been measured thus far. Here, we employed the recently developed, facile, solution-based technique, microscale thermophoresis^38,39^, to estimate the relative binding affinities of several cognate and non-cognate HK-RR pairs from within the sets that were shown to cross-talk in vitro. As we indicated above, we found for every HK we examined that at least one, but often multiple, non-cognate RRs existed that had higher affinity for the HK than the cognate counterpart. This finding, together with the recognition that phosphotransfer to non-cognate RRs is slow and inefficient compared to cognate RRs^27^, provided the basis for sequestration as a regulatory design principle that we unraveled.

We recognized that inferences from microscale thermophoresis experiments could be confounded by phosphotransfer from HK ~ P to RR. If such transfer were substantial, HK~P and RR may not remain the dominant species in the reaction mix. To address this concern, we generated mutants of MtrA and NarL that were incapable of accepting phosphoryl groups and repeated our measurements. Furthermore, we employed two additional, independent affinity measurement techniques, isothermal titration calorimetry, and BLI. In all these cases, we found the results to be consistent, with NarL exhibiting higher affinity than MtrA for MtrB~P, giving us confidence in our findings. With the other HK ~ P/RR pairs we studied (Fig. 2), we therefore relied on microscale thermophoresis. Future studies may establish the robustness of these latter measurements, by generating mutants or using other assays.

Evidence of the generality of the design comes also from the extent of conservation of the TCSs we studied across mycobacterial species. A recent study examined this conservation across 11 mycobacterial species^23^. The study found that the TCS MtrAB is conserved across all the 11 species. The NarLS system is conserved across 10 of the 11 species. Thus, the sequestration of MtrB~P by NarL that we examined here might be manifested across 10 mycobacterial species. Among the other TCSs we studied, PrrAB is conserved across all the 11 species and so is its non-cognate RR MprA, identifying another potential pan-mycobacterial applicability of our findings. The other TCSs we studied were present in several, if not all, mycobacterial species. Further, given the generality of the design, we expect it to hold beyond mycobacteria.

The extent of regulation by this design would depend on the relative binding affinities as well as the expression levels of the cognate and non-cognate RRs. In our in vivo studies, we demonstrated that increasing the levels of the higher affinity non-cognate RR NarL reduced the output of the TCS MtrAB. The sequestration motif may be prevalent even with non-cognate RRs that have lower binding affinities than the cognate ones if their expression levels are sufficiently high. In effect, one may view the collection of all non-cognate RRs as the source of sequestration. The abundance of RRs is typically greater than their cognate HKs^40–43^. A typical bacterial system may contain many tens to hundreds of distinct TCSs, thus providing a large pool of non-cognate RRs for any HK^44^. Furthermore, we speculate that “orphan” RRs, for which cognate HKs are unidentified^31^, may be functioning as sequesters.

In settings where sequestration is due to non-cognate RRs that bind more strongly to an HK than its cognate counterpart, targeting the non-cognate RR-HK binding may be a potential intervention strategy. It may compromise the ability of the bacterium to withhold responses to frivolous stimuli, draining its resources and reducing its viability. Further, the high affinity binding between the HK and the non-cognate RR suggests the existence of specific binding regions, which could be targeted by drugs or vaccines.

Finally, we recognize potential implications of our findings for applications in synthetic biology. Both positive autoregulation and sequestration have independently been recognized as important motifs in synthetic biological constructs^7,45^. Positive autoregulation has been shown to introduce thresholds in the stimulus strength for eliciting responses and amplify cellular heterogeneity^7,46^. For instance, positive autoregulation has been argued to drive cells infected by HIV into latent or virus-producing states^47^. Sequestration is often viewed as a motif for achieving negative feedback and control^20,48^. For instance, in a recent computational study, non-cognate RRs, rather than the classical cognate HK-RR pairs, placed downstream of the cognate signaling pathway have been proposed as a negative feedback mechanism to achieve control by increasing sequestration in proportion to the stimulus strength and thus regulating the response^48^. Here, by combining sequestration with positive autoregulation, we identify a new (naturally occurring) motif that enables finer control of signal transduction. The motif prevents responses to sub-threshold stimuli and enables robust responses to stronger stimuli. Furthermore, the threshold may be tuned by designing or choosing sequesters with the right affinities and/or controlling their concentrations.

In summary, our study presents evidence of a new feature of TCS signal regulation, where sequestration by non-cognate RRs ensures that responses are mounted only after a threshold level of stimulation is realized.

## Methods

### Mathematical model of TCS signaling with sequestration of HK by non-cognate RR

Our model describes the signal transduction via TCS in the presence of non-cognate RR (Eqs. 1–19). We wrote the following rate equations to describe the dynamics of signal transduction following stimulation by ligand, *I*. The equations build on earlier models of TCS signaling^21,49,50^ and advance them by incorporating the role of non-cognate RRs.

20 $$\frac{d[H{K}_{basal}]}{dt}=	-{k}_{f}^{lig}[H{K}_{basal}][I]+{k}_{d}^{lig}[HK]+\lambda \beta {P}_{T}\left(\frac{1+\alpha \frac{{\left[R{R}_{c}^{\ast }\right]}^{2}}{K}}{1+\frac{{\left[R{R}_{c}^{\ast }\right]}^{2}}{K}}\right)\\ 	 -{k}_{dp}^{RR}[H{K}_{basal}][R{R}_{c}^{\ast }]+ {k}_{d}^{tc}[H{K}_{basal}-P-R{R}_{c}] \\ 	+{k}_{d}^{tc-nc}[H{K}_{basal}-P-R{R}_{nc}]-{k}_{{{{{{{\rm{deg}}}}} }}}[H{K}_{basal}]$$

21 $$\frac{d[HK]}{dt}=	{k}_{f}^{lig}[H{K}_{basal}][I]-{k}_{d}^{lig}[HK]-{k}_{f}^{ATP}[HK][ATP] \\ 	+ ({k}_{f}^{ATP}/{K}_{E})[HK-ATP]+ {k}_{p}^{RR}[HK-P-R{R}_{c}]\\ 	-{k}_{dp}^{RR}[HK][R{R}_{c}^{\ast }]+{k}_{d}^{tc}[HK-P-R{R}_{c}^{\ast }] \\ 	+ {k}_{d}^{tc-nc}[HK-P-R{R}_{nc}]-{k}_{f}^{RR}[HK][R{R}_{c}] \\ 	+ {k}_{f}^{RR}\cdot {K}_{D}^{HK}[HK-R{R}_{c}]-{k}_{f}^{RR-nc}[HK][R{R}_{nc}]\\ 	+ {k}_{f}^{RR-nc}\cdot {K}_{D-nc}^{HK}[HK-R{R}_{nc}]-{k}_{{{{{{{\rm{deg}}}}} }}}[HK]$$

22 $$\frac{d[HK-ATP]}{dt}=	{k}_{f}^{ATP}[HK][ATP]-({k}_{f}^{ATP}/{K}_{E})[HK-ATP]-{k}_{p}[HK-ATP]\\ 	+ {k}_{f}^{lig}[H{K}_{basal}-ATP][I]-{k}_{d}^{lig}[HK-ATP]-{k}_{{{{{{{\rm{deg}}}}} }}}[HK-ATP]$$

23 $$\frac{d[H{K}^{\ast }]}{dt}=	{k}_{p}[HK-ATP]-{k}_{f}^{RR}[H{K}^{\ast }][R{R}_{c}]+{k}_{f}^{RR}\cdot {K}_{D}[H{K}^{\ast }-R{R}_{c}]\\ 	-{k}_{f}^{RR-nc}[H{K}^{\ast }][R{R}_{nc}]+{k}_{f}^{RR-nc}\cdot {K}_{D-nc}[H{K}^{\ast }-R{R}_{nc}]\\ 	+ {k}_{f}^{lig}[H{K}_{basal}^{\ast }][I]-{k}_{d}^{lig}[H{K}^{\ast }]-{k}_{{{{{\rm{deg}}}}} }[H{K}^{\ast }]$$

24 $$\frac{d[R{R}_{c}]}{dt}=	-{k}_{f}^{RR}[H{K}^{\ast }][R{R}_{c}]+{k}_{f}^{RR}\cdot {K}_{D}[H{K}^{\ast }-RR]+{k}_{d}^{tc}[HK-P-R{R}_{c}]\\ 	+ {k}_{d}^{tc}[H{K}_{basal}-P-R{R}_{c}]+\beta {P}_{T}\left(\frac{1+\alpha \frac{{[R{R}_{c}^{\ast }]}^{2}}{{K}_{1}}}{1+\frac{{[R{R}_{c}^{\ast }]}^{2}}{{K}_{1}}}\right)+{k}_{d}^{RR}[R{R}_{c}^{\ast }]-{k}_{f}^{RR}[HK][R{R}_{c}]\\ 	+ {k}_{f}^{RR}\cdot {K}_{D}^{HK}[HK-R{R}_{c}]-{k}_{{{{{\rm{deg}}}}} }[R{R}_{c}]$$

25 $$\frac{d[R{R}_{c}^{\ast }]}{dt}=	{k}_{p}^{RR}[HK-P-R{R}_{c}]-{k}_{dp}^{RR}[HK][R{R}_{c}^{\ast }]-{k}_{d}^{RR}[R{R}_{c}^{\ast }]\\ 	-{k}_{dp}^{RR}[H{K}_{basal}][R{R}_{c}^{\ast }]-{k}_{{{{{\rm{deg}}}}} }[R{R}_{c}^{\ast }]$$

26 $$\frac{d[R{R}_{nc}]}{dt}=	-{k}_{f}^{RR-nc}[H{K}^{\ast }][R{R}_{nc}]+{k}_{f}^{RR-nc}\cdot {K}_{D-nc}[H{K}^{\ast }-R{R}_{nc}]\\ 	-{k}_{f}^{RR}[HK][R{R}_{c}]+{k}_{f}^{RR}\cdot {K}_{D}^{HK}[HK-R{R}_{c}]+{k}_{d}^{tc-nc}[HK-P-R{R}_{nc}]\\ 	+ {k}_{d}^{tc-nc}[H{K}_{basal}-P-R{R}_{nc}]-{k}_{{{{{\rm{deg}}}}} }[R{R}_{nc}]$$

27 $$\frac{d[HK-R{R}_{c}]}{dt}=	{k}_{f}^{RR}[HK][R{R}_{c}]-{k}_{f}^{RR}\cdot {K}_{D}^{HK}[HK-R{R}_{c}]\\ 	+ {k}_{f}^{lig}[H{K}_{basal}-R{R}_{c}][I]-{k}_{d}^{lig}[HK-R{R}_{c}]-{k}_{{{{{\rm{deg}}}}} }[HK-R{R}_{c}]$$

28 $$\frac{d[H{K}^{\ast }-R{R}_{c}]}{dt}=	{k}_{f}^{RR}[H{K}^{\ast }][R{R}_{c}]-{k}_{f}^{RR}\cdot {K}_{D}[H{K}^{\ast }-R{R}_{c}]-{k}_{f}^{tc}[H{K}^{\ast }-R{R}_{c}]\\ 	+ {k}_{f}^{tc}\cdot {K}_{tc}[H{K}_{basal}-P-R{R}_{c}]+{k}_{f}^{lig}[H{K}_{basal}^{\ast }-R{R}_{c}][I]\\ 	-{k}_{d}^{lig}[H{K}^{\ast }-R{R}_{c}]-{k}_{{{{{\rm{deg}}}}} }[H{K}^{\ast }-R{R}_{c}]$$

29 $$\frac{d[HK-P-R{R}_{c}]}{dt}=	{k}_{f}^{tc}[H{K}^{\ast }-R{R}_{c}]-{k}_{f}^{tc}\cdot {K}_{tc}[HK-P-R{R}_{c}]-{k}_{p}^{RR}[HK-P-R{R}_{c}]\\ 	-{k}_{d}^{tc}[HK-P-R{R}_{c}]+{k}_{dp}^{RR}[HK][R{R}_{c}^{\ast }]+{k}_{f}^{lig}[H{K}_{basal}-P-R{R}_{c}][I]\\ 	-{k}_{d}^{lig}[HK-P-R{R}_{c}]-{k}_{{{{{\rm{deg}}}}} }[HK-P-R{R}_{c}]$$

30 $$\frac{d[HK-R{R}_{nc}]}{dt}=	{k}_{f}^{RR}[HK][R{R}_{nc}]-{k}_{f}^{RR}\cdot {K}_{D-nc}^{HK}[HK-R{R}_{nc}]\\ 	+ {k}_{f}^{lig}[H{K}_{basal}-R{R}_{nc}][I]-{k}_{d}^{lig}[HK-R{R}_{nc}]-{k}_{{{{{\rm{deg}}}}} }[HK-R{R}_{nc}]$$

31 $$\frac{d[H{K}^{\ast }-R{R}_{nc}]}{dt}=	{k}_{f}^{RR-nc}[H{K}^{\ast }][R{R}_{nc}]-{k}_{f}^{RR-nc}\cdot {K}_{D-nc}[H{K}^{\ast }-R{R}_{nc}]\\ 	-{k}_{f}^{tc}[H{K}^{\ast }-R{R}_{nc}]+{k}_{f}^{tc}\cdot {K}_{tc}[HK-P-R{R}_{nc}]+{k}_{f}^{lig}[H{K}_{basal}^{\ast }-R{R}_{nc}][I]\\ 	-{k}_{d}^{lig}[H{K}^{\ast }-R{R}_{nc}]-{k}_{{{{{\rm{deg}}}}} }[H{K}^{\ast }-R{R}_{nc}]$$

32 $$\frac{d[HK-P-R{R}_{nc}]}{dt}=	{k}_{f}^{tc}[H{K}^{\ast }-R{R}_{nc}]-{k}_{f}^{tc}\cdot {K}_{tc}[HK-P-R{R}_{nc}]\\ 	-{k}_{d}^{tc-nc}[HK-P-R{R}_{nc}]+{k}_{f}^{lig}[H{K}_{basal}-P-R{R}_{nc}][I]\\ 	-{k}_{d}^{lig}[HK-P-R{R}_{nc}]-{k}_{{{{{\rm{deg}}}}} }[HK-P-R{R}_{nc}]$$

33 $$\frac{d[H{K}_{basal}-ATP]}{dt}=	-{k}_{f}^{lig}[H{K}_{basal}-ATP][I]+{k}_{d}^{lig}[HK-ATP]\\ 	-{k}_{{{{{\rm{deg}}}}} }[H{K}_{basal}-ATP]$$

34 $$\frac{d[H{K}_{basal}^{\ast }]}{dt}=-{k}_{f}^{lig}[H{K}_{basal}^{\ast }][I]+{k}_{d}^{lig}[H{K}^{\ast }]-{k}_{{{{{\rm{deg}}}}} }[H{K}_{basal}^{\ast }]$$

35 $$\frac{d[H{K}_{basal}-R{R}_{c}]}{dt}=	-{k}_{f}^{lig}[H{K}_{basal}-R{R}_{c}][I]+{k}_{d}^{lig}[HK-R{R}_{c}]\\ 	-{k}_{{{{{\rm{deg}}}}} }[H{K}_{basal}-R{R}_{c}]$$

36 $$\frac{d[H{K}_{basal}^{\ast }-R{R}_{c}]}{dt}=	-{k}_{f}^{lig}[H{K}_{basal}^{\ast }-R{R}_{c}][I]+{k}_{d}^{lig}[H{K}^{\ast }-R{R}_{c}]\\ 	-{k}_{{{{{\rm{deg}}}}} }[H{K}_{basal}^{\ast }-R{R}_{c}]$$

37 $$\frac{d[H{K}_{basal}-P-R{R}_{c}]}{dt}=	-{k}_{d}^{tc}[H{K}_{basal}-P-R{R}_{c}]+{k}_{dp}^{RR}[H{K}_{basal}][R{R}_{c}^{\ast }]\\ 	-{k}_{f}^{lig}[H{K}_{basal}-P-R{R}_{c}][I]+{k}_{d}^{lig}[HK-P-R{R}_{c}]\\ 	-{k}_{{{{{\rm{deg}}}}} }[H{K}_{basal}-P-R{R}_{c}]$$

38 $$\frac{d[H{K}_{basal}-R{R}_{nc}]}{dt}=	-{k}_{f}^{lig}[H{K}_{basal}-R{R}_{nc}][I]+{k}_{d}^{lig}[HK-R{R}_{nc}]\\ 	-{k}_{{{{{\rm{deg}}}}} }[H{K}_{basal}-R{R}_{nc}]$$

39 $$\frac{d[H{K}_{basal}^{\ast }-R{R}_{nc}]}{dt}=	-{k}_{f}^{lig}[H{K}_{basal}^{\ast }-R{R}_{nc}][I]+{k}_{d}^{lig}[H{K}^{\ast }-R{R}_{nc}]\\ 	-{k}_{{{{{\rm{deg}}}}} }[H{K}_{basal}^{\ast }-R{R}_{nc}]$$

40 $$\frac{d[H{K}_{basal}-P-R{R}_{nc}]}{dt}=	-{k}_{d}^{tc-nc}[H{K}_{basal}-P-R{R}_{nc}]\\ 	-{k}_{f}^{lig}[H{K}_{basal}-P-R{R}_{nc}][I]+{k}_{d}^{lig}[HK-P-R{R}_{nc}]\\ 	-{k}_{{{{{\rm{deg}}}}} }[H{K}_{basal}-P-R{R}_{nc}]$$

The equations were constructed by writing standard rate expressions for the events in Eqs. (1–19). Thus, the concentration of basal HK can change due to the following events: ligand binding and unbinding, autoregulation, phosphatase activity, and degradation. These form all the terms on the right-hand side of Eq. (20) above. Similar expressions were formulated for the other species (Eqs. 21–40). The meanings of the rate constants are in Table S4. We explain the non-obvious terms here. Following HK^*^ binding to *RR*_*c*_, the resulting complex, $H{K}^{\ast }-R{R}_{c}$, is assumed to form the transition complex $HK-P-R{R}_{c}$, poised for phosphotransfer to *RR*_*c*_. A similar model, where the phosphoryl group is transferred within the complex, has been used earlier^6^. The transition complex, $HK-P-R{R}_{c}$, can either effect phosphotransfer yielding *HK* and $R{R}_{c}^{\ast }$ or dissociate, releasing inorganic phosphate (*P*_*i*_) and leaving behind unphosphorylated *HK* and *RR*_*c*_. This formalism is different from the classical phosphatase activity found in other models^21,49^, which ignore the transition complex. We considered the complex because of its importance to non-cognate RR binding. The non-cognate RR, RR_nc_, could form the analogous transition complex, $HK-P-R{R}_{nc}$, but the latter complex was assumed not to be able to effect phosphotransfer. Because phosphatase activity is generally slower than phosphotransfer, the latter complex becomes relatively long-lived and acts as a sequester.

We note here that our formalism is consistent with the dimerization of HK typically observed^29^. HK typically exists as symmetric homodimers, which function primarily as phosphatases. Ligand binding renders them asymmetric and lets their primary function change to autophosphorylation and phosphotransfer. Because of the separation of functions, the symmetric and asymmetric HK dimers become analogous to the basal and ligand-bound forms of HK in our model. The rest of the signal transduction process follows the same steps as in our model. The only change is that translation now produces HK monomers, which then must dimerize. The latter step does not alter the behavior of our model. Also, our model explicitly considers RR dimerization. Thus, our model is consistent with the current understanding of HK and RR dimerization. We note further that variations in these features – namely, the activities of basal and ligand-bound forms of HK or the relative concentrations of their monomeric and dimerized states – do not influence our key conclusions. The effect of sequestration by non-cognate RRs, of interest here, is expected independently of these variations.

The autoregulation leading to the expression of HK and RR proteins was modeled using the pseudo-equilibrium approximation following earlier studies^21,50^. We let *P*_*T*_ be the total concentration of promoter binding sites present on the bacterial genome. If *f*_*b*_ and *f*_*f*_ were the fractions of promoter sites in the bound and free states respectively, then the equilibrium of the events in Eq. 16 would yield

41 $${k}_{f}^{DNA}{f}_{f}{P}_{T}{\left[R{R}_{c}^{\ast }\right]}^{2}={k}_{d}^{DNA}{f}_{b}{P}_{T}$$

If we let $K=\frac{{k}_{d}^{DNA}}{{k}_{f}^{DNA}}$ be the corresponding equilibrium dissociation coefficient, and since ${f}_{f}+{f}_{b}=1$, it followed that

42 $${f}_{f}=\frac{1}{1+\frac{{\left[R{R}_{c}^{\ast }\right]}^{2}}{K}}\,{{{{{\rm{and}}}}}}\,{f}_{b}=\frac{1}{1+\frac{K}{{\left[R{R}_{c}^{\ast }\right]}^{2}}}$$

The change of mRNA concentration can be written from Eqs. 14, 15 as

43 $$\frac{d[m]}{dt}={{{{{{\rm{k}}}}}}}_{{{{{{\rm{btpn}}}}}}}\,{f}_{f}{P}_{T}+{{{{{{\rm{k}}}}}}}_{{{{{{\rm{tpn}}}}}}}\,{f}_{b}{P}_{T}-{{{{{{\rm{k}}}}}}}_{{{{{\rm{deg}}}}} }^{m}[m]$$

Applying the pseudo-equilibrium approximation^21,50^ for mRNA dynamics, i.e., $\frac{dm}{dt} \, \approx \, 0$, and using Eq. (42), we obtained

44 $$[m]=\frac{{{{{{{\rm{k}}}}}}}_{{{{{{\rm{btpn}}}}}}}{P}_{T}}{{{{{{{\rm{k}}}}}}}_{{{{{\rm{deg}}}}} }^{m}}\frac{\left(1+\frac{{{{{{{\rm{k}}}}}}}_{{{{{{\rm{tpn}}}}}}}}{{{{{{{\rm{k}}}}}}}_{{{{{{\rm{btpn}}}}}}}}\frac{{[R{R}_{c}^{\ast }]}^{2}}{K}\right)}{1+\frac{{[R{R}_{c}^{\ast }]}^{2}}{K}}$$

Translation of the mRNA molecules results in the production of the two TCS proteins *HK*_*basal*_ and *RR* molecules, with *λ* the ratio of the two production rates^21,50^.

45 $$\frac{d[H{K}_{basal}]}{dt}=\lambda {{{{{{\rm{k}}}}}}}_{{{{{{\rm{trn}}}}}}}[m]\,{{{{{\rm{and}}}}}}\,\frac{d[R{R}_{c}]}{dt}={{{{{{\rm{k}}}}}}}_{{{{{{\rm{trn}}}}}}}[m]$$

Substituting $\frac{{{{{{{\rm{k}}}}}}}_{{{{{{\rm{trn}}}}}}}{{{{{{\rm{k}}}}}}}_{{{{{{\rm{btpn}}}}}}}}{{{{{{{\rm{k}}}}}}}_{{{{{\rm{deg}}}}} }^{m}}=\beta$ and $\frac{{{{{{{\rm{k}}}}}}}_{{{{{{\rm{tpn}}}}}}}}{{{{{{{\rm{k}}}}}}}_{{{{{{\rm{btpn}}}}}}}}=\alpha$, we obtained the synthesis rates of *HK*_*basal*_ and *RR*_*c*_ by translation as

46 $$\frac{d[H{K}_{basal}]}{dt}=\lambda \beta {P}_{T}\left(\frac{1+\alpha \frac{{[R{R}_{c}^{\ast }]}^{2}}{K}}{1+\frac{{[R{R}_{c}^{\ast }]}^{2}}{K}}\right)\,{{{{{\rm{and}}}}}}\,\frac{d[R{R}_{c}]}{dt}=\beta {P}_{T}\left(\frac{1+\alpha \frac{{[R{R}_{c}^{\ast }]}^{2}}{K}}{1+\frac{{[R{R}_{c}^{\ast }]}^{2}}{K}}\right)$$

which we used in Eqs. (20) and (24) above to describe autoregulation. We defined the output, *O*, of the signal transduction events as the fraction of bound promoter regions, *f*_*b*_, so that:

47 $$O=\frac{{\left[R{R}_{c}^{\ast }\right]}^{2}}{K+{\left[R{R}_{c}^{\ast }\right]}^{2}}$$

### Data fitting and parameter estimation

The parameter values employed are listed in Table S4 along with their sources. The dissociation constants between the cognate pairs MtrB and MtrA, *K*_*D*_, and non-cognate pairs MtrB and NarL, ${K}_{D-nc}$, were estimated using microscale thermophoresis (Fig. 1). The autophosphorylation of MtrB has been studied earlier and the equilibrium constant, *K*_*E*_, and the phosphorylation rate, ${k}_{p}$, estimated^30^. ${\phi }_{HK}$, the fraction of MtrB active was also estimated^30^. The natural dephosphorylation rate of $R{R}_{c}^{\ast }$, ${k}_{d}^{RR}$, has been estimated for PhoB^28^. We assumed the same natural dephosphorylation rate for the other RRs. The activation and deactivation rates for HKs, ${k}_{f}^{lig}$ and ${k}_{d}^{lig}$, the forward rates for *HK* binding to *ATP*, ${k}_{f}^{ATP}$, and of HK^*^ binding to *RR*_c_ and *RR*_nc_, ${k}_{f}^{RR}$ and ${k}_{f}^{RR-nc}$, respectively, as well as the total promoter concentration, *P*_*T*_, were chosen from an earlier study^49^. The equilibrium dissociation constant for DNA binding, *K*, the transcription rate, *α*, the translation rate, *β*, and the ratio of HK and RR produced by translation, *λ*, were from another previous study^50^.

The unknown parameters were the percentage activity of RR_c_, ${\phi }_{RR}$, the equilibrium dissociation constant of the transition complex, ${K}_{tc}$, the phospho-transfer rate constant between MtrB and MtrA, ${k}_{p}^{RR}$, the binding rate constant of *HK* and $R{R}_{c}^{\ast }$, ${k}_{dp}^{RR}$, the phosphatase activity rate constant between MtrB and MtrA, ${k}_{d}^{tc}$, and the phosphatase activity rate constant between MtrB and NarL, ${k}_{d}^{tc-nc}$. We assumed that the phosphotransfer rate from MtrB to NarL was negligible and set it to zero. The forward rate of transition complex formation, ${k}_{f}^{tc}$, was assumed to be 100 min^−1^, to represent fast dynamics typically associated with transition complexes compared to other processes.

To estimate the unknown parameters, we simultaneously fit our model to data from the three time course assays in Fig. 3. The termination of reactions resulted in denaturing of protein complexes. Therefore, while measuring HK^*^ after reaction termination, we assumed that the complexes $H{K}^{\ast }-R{R}_{c}$ and $H{K}^{\ast }-R{R}_{nc}$ could denature to HK^*^ so that the concentration which we measured at each time point was the sum of HK^*^, $H{K}^{\ast }-R{R}_{c}$ and $H{K}^{\ast }-R{R}_{nc}$ species. All the concentrations were normalized to the initial total active HK, ${\phi }_{HK}$. We recognized that several processes in the in vivo model, such as autoregulation, would not apply in vitro. We therefore used multiple models (Note S2) with increasing levels of complexity to describe the in vitro data (Fig. 3). We solved the models using the MULTISTART function in MATLAB to do a global parameter search and used the LSQCURVEFIT function in MATLAB for optimization. The model equations were solved using ODE23 in MATLAB. We ensured that repeated optimization yielded the same results. We compared the different models using the Akaike information criterion (AICc) and chose the best one (Table S5). The data constrained the model well, yielding best-fit estimates with reliable confidence intervals. The resulting simplified model was

48 $$({{{{{\rm{ATP}}}}}}\,{{{{{\rm{binding}}}}}})\,HK+ATP \mathop{\rightleftharpoons }\limits_{{k}_{f}^{ATP}/{K}_{E}}^{{{k}_{f}^{ATP}}}HK-ATP$$

49 $$({{{{{\rm{Autophosphorylation}}}}}})\,HK-ATP\mathop{\longrightarrow }\limits^{{k}_{p}}H{K}^{\ast }+ADP$$

50 $$({{{{{\rm{Cognate}}}}}}\,{{{{{\rm{RR}}}}}}\,{{{{{\rm{binding}}}}}}\,HK)\,HK+R{R}_{c} \mathop{\rightleftharpoons }\limits_{{k}_{f}^{RR}\cdot {K}_{D}^{HK}}^{{{k}_{f}^{RR}}}HK-R{R}_{c}$$

51 $$({{{{{\rm{Cognate}}}}}}\,{{{{{\rm{RR}}}}}}\,{{{{{\rm{binding}}}}}}\,H{K}^{\ast })\,H{K}^{\ast }+R{R}_{c} \mathop{\rightleftharpoons }\limits_{{k}_{f}^{RR}\cdot {K}_{D}}^{{{k}_{f}^{RR}}}H{K}^{\ast }-R{R}_{c}$$

52 $$({{{{{\rm{Transition}}}}}}\,{{{{{\rm{complex}}}}}}\,{{{{{\rm{formation}}}}}})\,H{K}^{\ast }-R{R}_{c}\mathop{\rightleftharpoons }\limits_{{k}_{f}^{tc}\cdot {K}_{tc}}^{{{k}_{f}^{tc}}}HK-P-R{R}_{c}$$

53 $$({{{{{\rm{Phosphotransfer}}}}}})\,HK-P-R{R}_{c} \mathop{\rightleftharpoons }\limits_{{k}_{dp}^{RR}}^{{{k}_{p}^{RR}}}HK+R{R}_{c}^{\ast }$$

54 $$({{{{{\rm{Phosphatase}}}}}}\,{{{{{\rm{activity}}}}}})\,HK-P-R{R}_{c}\mathop{\longrightarrow }\limits^{{k}_{d}^{tc}}HK+R{R}_{c}+{P}_{i}$$

55 $$({{{{{\rm{Noncognate}}}}}}\,{{{{{\rm{RR}}}}}}\,{{{{{\rm{binding}}}}}}\,HK)\,HK+R{R}_{nc} \mathop{\rightleftharpoons }\limits_{{k}_{f}^{RR-nc}\cdot {K}_{D-nc}^{HK}}^{{{k}_{f}^{RR-nc}}}HK-R{R}_{nc}$$

56 $$({{{{{\rm{Noncognate}}}}}}\,{{{{{\rm{RR}}}}}}\,{{{{{\rm{binding}}}}}}\,H{K}^{\ast })\,H{K}^{\ast }+R{R}_{nc} \mathop{\rightleftharpoons }\limits_{{k}_{f}^{RR-nc}\cdot {K}_{D-nc}}^{{{k}_{f}^{RR-nc}}}H{K}^{\ast }-R{R}_{nc}$$

57 $$({{{{{\rm{Transition}}}}}}\,{{{{{\rm{complex}}}}}}\,{{{{{\rm{formation}}}}}})\,H{K}^{\ast }-R{R}_{nc}\mathop{\rightleftharpoons }\limits_{{k}_{f}^{tc}\cdot {K}_{tc}}^{{{k}_{f}^{tc}}}HK-P-R{R}_{nc}$$

58 $$({{{{{\rm{Phosphatase}}}}}}\,{{{{{\rm{activity}}}}}})\,HK-P-R{R}_{nc}\mathop{\longrightarrow }\limits^{{k}_{d}^{tc-nc}}HK+R{R}_{nc}+{P}_{i}$$

59 $$({{{{{\rm{Dephosphorylation}}}}}})\,R{R}_{c}^{\ast }\mathop{\longrightarrow }\limits^{{k}_{d}^{RR}}R{R}_{c}+{P}_{i}$$

We accordingly used a subset of the rate equations above, restricting the equations to those associated with the simplified model, and estimated the unknown parameters from the fits. We thus estimated the six parameters: ${\phi }_{RR}$, ${K}_{tc}$, ${k}_{p}^{RR}$, ${k}_{dp}^{RR}$, ${k}_{d}^{tc}$, and ${k}_{d}^{tc-nc}$. The best-fit estimates and the associated confidence intervals are in Table S4.

### Molecular modeling of HK:RR complexes

Sequence alignments of queries and templates were generated using PSI-blast^51^. Structural models were generated using the homology-based modeling tool MODELLER v9.12^52^. Side-chain conformations of the models with the highest DOPE score^53^ and best GA341 score^54^ were refined using the SCRWL 4.0 library^55^ and the structures were energy minimized using GROMACS(v5.1)^56^ with conjugate gradient as step integrator to remove short-contacts, if any. FoldX(4.0) Repair PDB^57^ was also used to obtain a stable complex. Stereochemical quality of the refined models was ensured using PROCHECK(v3)^58^ and MolProbity^59^. Further analyses have been done using these models. Interface residues of HK:RR complexes were identified from the structural models using the standard van der Waals radii cut-offs, where any two residues are considered to be interacting if the distance between any two atoms in the residues is at most 0.5 Å greater than the summation of the respective van der Waals radii^60^. The nature of the interactions between the interacting residues was identified using the in-house protein interaction calculator (PIC)^61^. Interface regions of complexes were then visually scanned using PyMol.

### Experimental methods

#### Materials

All the media chemicals, biochemicals, and protein reagents were purchased from Sigma Merck (USA); antibiotics and DTT (Dithiothreitol) from Goldbio (USA); protein markers were from Thermo Fisher (USA), agarose-GSH resin and Ni^+2^-NTA resin were from GE Healthcare (USA); and restriction enzymes from Thermo Fisher (USA). Primers were synthesized from Bioserve (India) and γ^32^P ATP ( > 3500 Ci/mmol) was purchased from BRIT-Jonaki (India).

#### Bacterial strains and plasmids

Protein overexpression was carried out in *E. coli* Origami and Origami B (Novagen Inc., USA). These strains carrying the recombinant plasmids were propagated in LB containing ampicillin (100 μg/ml). The recombinant plasmids used for protein overexpression have been reported previously^21^. In brief, for the HKs, the plasmids containing only the cytosolic catalytic domains were used and for RRs the entire coding gene was used. For all GFP (Green Fluorescent Protein) tagged proteins, the GFP gene was cloned downstream of cytosolic catalytic domain of the respective HK with a linker region that encoded for the GSGGG spacer peptide, which facilitated functional separation of the two proteins as reported previously^24^.

#### Recombinant protein purification

For recombinant protein overexpression and purification, *E. coli* strains carrying the recombinant plasmid were grown at 37 °C, in 200 ml of 2x YT broth until OD600 of 0.4−0.6, then IPTG (0.5 mM) was added to the culture and incubated further for 15–20 h at 12–15 °C for protein overexpression. Cells were harvested by centrifugation and stored until use at −80 °C. For protein purification in soluble conditions, the protocol described previously was followed^24^.

#### Autophosphorylation and phosphotransfer activity of GFP-tagged HKs using PAGE/autoradiography

The functional validation of the activity of GFP-tagged HKs was conducted as previously reported for MtrB-GFP^27^. In brief, 5 μM of the purified GFP-tagged HK, i.e., PhoR-GFP was incubated in the kinase buffer (50 mM Tris-HCl, pH 8.0, 50 mM KCl, 10 mM MgCl_2_) containing 50 μM ATP and 1 μCi of γ^32^P-labeled ATP at 30 °C for 2 h (Fig. S8). Equimolar amount of the recombinant cognate RR PhoP diluted in kinase buffer was added to allow phosphotransfer for 5 min. The reaction was terminated by adding 1× SDS-PAGE sample buffer. The samples were resolved on 12% v/v SDS-PAGE. After electrophoresis, the gel was washed and exposed to phosphor screen (Fujifilm Bas cassette2, Japan) for 4 h followed by imaging with Typhoon 9210 phosphorimager (GE Healthcare, USA) and Azure Sapphire (Azure Biosystems, USA).

#### Determination of affinities of HK~P for RR using microscale thermophoresis (MST)

In all, 5 μM of the purified GFP-tagged HK was autophosphorylated as mentioned above using 50 μM ATP at 30 °C for 90 min. The autophosphorylated HK~P (50 nM) was mixed with increasing concentrations of titrant RRs (concentration range mentioned in figure legends), in the autophosphorylation buffer and kept at 30 °C for 5 min. The sample was then loaded into standard treated capillaries and analyzed using a Monolith NT.115 (NanoTemper Technologies Germany). The blue laser was used for a duration of 35 s for excitation (MST power = 60%, LED power 40%). For the interactions involving fluorescently labeled recombinant proteins, the HKs were autophosphorylated and then mixed with 50 nM of Monolith NT His-tag labelling Kit Red Tris-NTA (L008; nanotemper Technologies Germany) incubated at 30 °C for 30 min to allow labeling of the His-tag with the red fluorescent dye NT-647 and the red laser was used for a duration of 35 s for excitation (MST power = 60%, LED power 90%). The data were analyzed using MO Control software (NanoTemper Technologies Germany) to determine the *K*_*D*_ for the interacting proteins.

#### Time course analysis of phosphotransfer assays

In the phosphotransfer assays, 100 pmoles of the RRs diluted in kinase buffer were added to the reaction containing 50 pmoles of autophosphorylated HK, followed by incubation at 30 °C for indicated time points. The reaction was terminated after the incubation by adding 1x SDS-PAGE gel loading buffer and resolved on a 15% SDS-PAGE gel. After electrophoresis, the gels are washed with deionized water and exposed on a phosphorscreen (Fujifilm, Japan) followed by imaging with Typhoon phosphorimager (GE Healthcare, USA). Semi-quantitative densitometric analysis of the autoradiograms was done using ImageJ software.

### RNA extraction, reverse transcription, and quantitative gene expression analysis

*Mycobacterium bovis* BCG cultures harboring either pTic6 vector alone or containing the *narL* gene were grown in 10 ml 7H9 medium supplemented with 1xADC and 0.05% tween 80 till the mid-log phase. Expression of NarL was induced with 10 and 50 ng/mL anhydrotetracyline (aTC) for 8 h. Total RNA was extracted using the RNeasy Mini Kit (Qiagen, USA) according to the manufacturer’s prescribed protocol from the exponentially grown cultures. RNA yield was quantified using the NanoDrop® ND-1000 UV-Vis Spectrophotometer (NanoDrop Technologies, USA). DNAse digestion was performed with TURBO DNA-free kit (invitrogen, Thermo Fisher Scientific) using manufacturer’s protocol. Approximately 500 ng of DNAse digested purified RNA was used to make cDNA using the iScript™ cDNA synthesis kit (Bio-Rad, USA) using the manufacturer’s protocol. Quantitative real time PCR (qRT-PCR) was performed with 10 µl of the cDNA reaction using the PowerUp SYBR green (Appliedbiosystems, Thermo Fisher Scientific) in the QIAquant 96 5plex(Qiagen, USA) according to the manufacturer’s protocol.

### Isothermal Titration calorimetry

Isothermal titration calorimetry (ITC) experiment was carried out in a MicroCal VP-ITC calorimeter. Different protein solutions were prepared in buffer (50 mM Tris-HCl pH-8.0, 100 mM NaCl, 25 mM KCl, 10 mM MgCl_2_), and MtrB solution was incubated for 30 min with 1 mM ATP to prepare MtrB~P. The reference cell of the calorimeter was filled with the same buffer (50 mM Tris-HCl pH-8.0, 100 mM NaCl, 25 mM KCl, 10 mM MgCl_2_) solution. Then different protein solutions were placed in the sample cell, and MtrB~P was titrated with the solution present in the cell at 25 °C. Automated addition of 5 μL titrant was continued for 55 injections with a time interval of 120 s and stirring speed was 307 rpm. The duration of each addition of titrant was 10 s, and filter spacing between two injections was 2 s. The integrated data were fitted by Origin 7.0 software.

#### Biolayer interferometry

BLI was performed in the Octet system (Sartorius, USA) using 96-well black plates. Briefly, we labeled MtrB with biotin and the biotinylated-MtrB was incubated for 30 min with 1 mM ATP to prepare MtrB~P. The biotinylated MtrB~P was then immobilized on a streptavidin biosensor surface and titrated with different concentrations of the RRs, either MtrA or NarL. The shift in the interference pattern, reflective of the number of bound complexes, was recorded during the binding and unbinding phases of the experiment and the data was analyzed to obtain estimates of the binding affinities of MtrB~P for the RRs. GraphPad Prism 8.4.2 was used for BLI data representation.

### Reporting summary

Further information on research design is available in the Nature Portfolio Reporting Summary linked to this article.

## Supplementary information

Supplementary Information

Reporting Summary

## Data Availability

The experimental data generated in this study are provided in the Supplementary Information and Source Data file, and have been deposited in the Zenodo database under accession code 115507 (10.5281/zenodo.8132755) as a consolidated source file and raw images files along with BLI data. Source data are provided with this paper.

## Source data

Source Data

## Code availability

The computer code used to implement mathematical modeling is available on GitHub (https://github.com/rubeshr1991/tcs_project.git), indexed at Zenodo database as 10.5281/zenodo.8130204.
