# Supplementary material for: Sequestration of histidine kinases by non-cognate response regulators establishes a threshold level of stimulation for bacterial two-component signaling
Source: Nat Commun. 2023 Jul 25;14:4483. doi: 10.1038/s41467-023-40095-2 (PMC10368727; doi:10.1038/s41467-023-40095-2)
Supplement: Supplementary file 1 — Supplementary Information [file 41467_2023_40095_MOESM1_ESM.docx]

**Supplementary Information for**

**Sequestration of histidine kinases by non-cognate response regulators establishes a threshold level of stimulation for bacterial two-component signaling**

**Note S1: Molecular modeling**

We employed molecular modeling to elucidate plausible origins of the tighter binding of the non-cognate RR NarL than the cognate RR MtrA to the HK MtrB. We constructed molecular models of the cognate complex MtrB:MtrA and the non-cognate complex MtrB:NarL. As a control, we also constructed the structure of the complex MtrB:PdtaR, where PdtaR is known not to bind MtrB. For comparison, we also considered the cognate pair NarL:NarS. Homology modelling was used to obtain structural models for the complexes using Modeller with a single template with the highest query coverage and sequence identity (PDB ID: 5UHT <https://doi.org/10.2210/pdb5UHT/pdb>). The sequence identity and similarity between template and query are in Table S2. We modeled the kinase domains of HK and the receiver domains of RR because of the absence of the other domains in the crystal structure of the template. The sequence alignment of the query with the template was good and only a few gaps were observed in the alignment. HK was modeled in the homodimeric state and each monomeric unit interacted with a molecule of RR (Figure S6). Next, we analysed the interfacial residues in the modelled HK:RR complexes using distance criteria. This analysis showed the presence of an extensive interaction interfaces in the MtrB:MtrA, NarS:NarL and MtrB:NarL complexes. The type of inter protein interactions and the residues involved were identified using the PIC server and are presented in Figure S7. Our manual analysis of the interfaces shows that the non-cognate MtrB:NarL interface is enriched in ionic interactions (Figure S7). Further, we estimated the interaction energies of the four HK:RR models using FoldX (Table S3). The non-cognate pair MtrB:NarL had a much higher interaction energy (-40 Kcal/mol) than the cognate pair MtrB:MtrA interaction (-15 Kcal/mol). As expected, PdtaR had hardly any interaction with MtrB (5 Kcal/mol). These estimates reinforce our findings *in vitro* of the higher affinity of NarL than MtrA for MtrB and suggest that they arise from enriched ionic interactions between NarL and MtrB.

**Note S2: Systematic model building and selection**

To describe the *in vitro* system, we constructed mathematical models with increasing levels of complexity and examined their ability to fit data (Figure 3). The models draw from existing reaction kinetics based models of HK autophosphorylation and phosphotransfer to RR[^1^](#_ENREF_1), [^2^](#_ENREF_2). We present the models below.

***Model 1:* *Minimal model of sequestration***

Here, HK is assumed to bind ATP reversibly (Eq. S1) and get phosphorylated (Eq. S2). Phosphorylated HK can transfer the phosphoryl group to its cognate RR (Eq. S3). HK can also exert phosphatase activity on phosphorylated RR (Eq. S4). Non-cognate RR can bind phosphorylated HK and sequester it, eventually triggering the loss of the phosphoryl group (Eq. S5).

(ATP binding) (S1)

(Autophosphorylation) (S2)

(Phosphotransfer) (S3)

(Phosphatase activity) (S4)

(Dephosphorylation) (S5)

***Model 2:* *Model of sequestration with all possible HK-RR complexes***

In this model, we expanded the events above to include all possible HK-RR complexes. Thus, phosphorylated or unphosphorylated HK could bind cognate or non-cognate RR (Eqs. S8, S9, S12 and S13). The sequestration of phosphorylated HK by non-cognate RR is now explicit in the complex formed in Eq. S13.

(ATP binding) (S6)

(Autophosphorylation) (S7)

(Cognate RR binding $HK$) (S8)

(Cognate RR binding $HK^{*}$) (S9)

(Phosphotransfer) (S10)

(Phosphatase activity) (S11)

(Noncognate RR binding $HK$) (S12)

(Noncognate RR binding $HK^{*}$) (S13)

(Phosphatase activity) (S14)

***Model 3:* *Model of sequestration with transition complexes***

Here, in addition to the HK-RR complexes, we considered transition complexes where HK is poised to transfer phosphoryl groups to RR (Eqs. S19 and S24). We also allowed for possible spontaneous dephosphorylation of phosphorylated RR (Eq. S26).

(ATP binding) (S15)

(Autophosphorylation) (S16)

(Cognate RR binding $HK$) (S17)

(Cognate RR binding $HK^{*}$) (S18)

(Transition complex formation) (S19)

(Phosphotransfer) (S20)

(Phosphatase activity) (S21)

(Noncognate RR binding $HK$) (S22)

(Noncognate RR binding $HK^{*}$) (S23)

(Transition complex formation) (S24)

(Phosphatase activity) (S25)

(Dephosphorylation) (S26)

We constructed rate equations for each of the models, following the procedure in the Methods, and fit the models to the data in Figure 3 (Methods, Figure S12). Using the best-fits, we estimated the Akaike Information Criterion for the models (Table S5).

**Table S1: Kinetic and thermodynamic parameters obtained from isothermal calorimetry.**

| **Species** | **K_D_ (μM)** | **K (M^-1^)** | **∆G (KJ/mol)** | **∆H (cal/mol)** | **∆S (cal/mol/deg)** | **N (Sites)** | **χ^2^/DOF** |
| --- | --- | --- | --- | --- | --- | --- | --- |
| **MtrB~P + MtrA** | 12.78 | (7.82 ± 0.94) ×10^4^ | -27.843 | 9505 ± 393.5 | 54.2 | 1 | 1.17 × 10^5^ |
| **MtrB~P + NarL** | 4.78 | (2.09 ± 0.27) × 10^5^ | -30.386 | 3948 ± 124.1 | 37.6 | 1 | 3.04 × 10^4^ |
| **MtrB~P + PdtaR** | N/O | N/O | N/O | N/O | N/O | N/O |  |

**K_D_**: equilibrium dissociation constant, **K**: binding constant, **∆G:** change in free energy**, ∆H**: change in enthalpy, **∆S**: change in entropy, **N**: number of binding sites, **χ^2^/DOF**: statistical parameter. The data were analyzed using a one site binding model. **N/O**: No heat change observed.

**Table S2. Sequence identity and similarity in comparison with template structure.**

| **Protein** | **Identity with template (%)** | **Similarity with template (%)** |
| --- | --- | --- |
| MtrB | 31 | 54 |
| MtrA | 34 | 58 |
| NarS | 35 | 50 |
| NarL | 33 | 53 |
| PdtaR | 35.5 | 57.9 |

**Table S3. Interaction energies of the HK:RR pairs determined using FoldX.**

| **Protein complex** | **Interaction energy (Kcal/mol)** |
| --- | --- |
| MtrB:NarL | -40 |
| MtrB:MtrA | -15 |
| MtrB:PdtaR | 5 |
| NarS:NarL | -30 |

**Table S4. Model parameters and their estimates**

| Symbol | Meaning | Value (95% CI) | Units | Source |
| --- | --- | --- | --- | --- |
| $k_{d}^{lig}$ | Ligand binding rate constant | $6\times10^{4}$ | $\text{min}^{\text{-1}}$ | Ref. [^3^](#_ENREF_3) |
| $k_{f}^{lig}$ | Ligand dissociation rate constant | $6\times10^{3}$ | $\text{min}^{\text{-1}}$ | Ref. [^3^](#_ENREF_3) |
| $k_{f}^{ATP}$ | ATP binding rate constant | $0.06$ | $\text{nM}^{\text{-1}}\text{min}^{\text{-1}}$ | Ref. [^2^](#_ENREF_2) |
| $K_{E}$ | ATP equilibrium association constant | $1.7\times10^{-6}$ | $\text{nM}^{\text{-1}}$ | Ref. [^2^](#_ENREF_2) |
| $k_{p}$ | Autophosphorylation rate constant | $0.27$ | $\text{min}^{\text{-1}}$ | Ref. [^2^](#_ENREF_2) |
| $k_{f}^{RR}$, $k_{f}^{RR-nc}$ | Binding rate constant of HK with cognate and non-cognate RR | $0.06$ | $\text{nM}^{\text{-1}}\text{min}^{\text{-1}}$ | Ref. [^3^](#_ENREF_3) |
| $K_{D}$ | Equilibrium dissociation constant of HK^*^-RR_c_ binding | $444$ | $\text{nM}$ | Present study (Fig. 1) |
| $K_{D-nc}$ | Equilibrium dissociation constant of HK^*^-RR_nc_ binding | $83$ | $\text{nM}$ | Present study (Fig. 1) |
| $K_{D}^{HK}$ | Equilibrium dissociation constant of HK-RR_c_ binding | 936 | $\text{nM}$ | Present study (Fig. S1) |
| $K_{D-nc}^{HK}$ | Equilibrium dissociation constant of HK-RR_nc_ binding | 402 | $\text{nM}$ | Present study (Fig. S1) |
| $k_{f}^{tc}$ | Rate constant of transition complex formation | $100$ | $\text{min}^{\text{-1}}$ | Assumed based on fast dynamics |
| $K_{tc}$ | Equilibrium dissociation constant of transition complex | $2.05\text{ }\left(1.16\text{ }-\text{ }3.63\right)$ | − | Present study (Fig. 3) |
| $k_{p}^{RR}$ | Phosphotransfer rate constant | $2.39\text{ }\left(1.22\text{ }-\text{ }4.66\right)$ | $\text{min}^{\text{-1}}$ | Present study (Fig. 3) |
| $k_{dp}^{RR}$ | Binding rate constant of HK and $RR_{c}$ | $8\times10^{-4}\text{ }\left(5\times10^{-4}\text{ }-\text{ }16\times10^{-4}\right)$ | $\text{nM}^{\text{-1}}\text{min}^{\text{-1}}$ | Present study (Fig. 3) |
| $k_{d}^{tc}$ | Rate constant of dissociation of transition complex | $0.86\text{ }\left(0.45\text{ }-\text{ }1.62\right)$ | $\text{min}^{\text{-1}}$ | Present study (Fig. 3) |
| $k_{d}^{tc-nc}$ | Rate constant of dissociation of transition complex containing RR_nc_ | $0.11\text{ }\left(0.05\text{ }-\text{ }0.25\right)$ | $\text{min}^{\text{-1}}$ | Present study (Fig. 3) |
| $k_{d}^{RR}$ | Dephosphorylation rate constant of $RR_{c}^{*}$ | $0.0144$ | $\text{min}^{\text{-1}}$ | Ref. [^4^](#_ENREF_4) |
| $P_{T}$ | Total promotor binding regions | $100$ | $\text{nM}$ | Ref. [^3^](#_ENREF_3) |
| $\alpha$ | Fold-increase in transcription due to $RR_{c}^{*}$ binding | $10$ | − | Ref. [^5^](#_ENREF_5) |
| $\beta$ | Effective rate constant of $RR_{c}$ synthesis | $0.36$ | $\text{nM} \text{min}^{\text{-1}}$ | Ref. [^5^](#_ENREF_5) |
| $\lambda$ | Rate of HK synthesis relative to $RR_{c}$ synthesis | $0.1$ | − | Ref. [^5^](#_ENREF_5) |
| $K$ | Equilibrium association constant of $RR_{c}$ with promoter | $5\times10^{5}$ | $\text{nM}^{2}$ | Ref. [^5^](#_ENREF_5) |
| $k_{\deg}$ | Degradation rate constant of proteins | $4.17\times10^{-4}$ | $\text{min}^{\text{-1}}$ | Ref. [^6^](#_ENREF_6) |
|  | Fraction of reaction competent HK | $0.14$ | − | Ref. [^2^](#_ENREF_2) |
|  | Fraction of reaction competent $RR_{c}$ | $0.036\text{ }\left(0.025\text{ }-\text{ }0.047\right)$ | − | Present study (Fig. 3) |

**Table S5. Model selection.** Akaike Information Criterion with the small sample size correction (AICc) of the models in Note S2 obtained from the best-fits shown in Figure S12. (The number of datapoints is n=25).

| **Model** | **AICc** |
| --- | --- |
| Model 1 | -46.7 |
| Model 2 | -59.8 |
| Model 3 | -83.9 |

**SUPPLEMENTARY FIGURES**

**
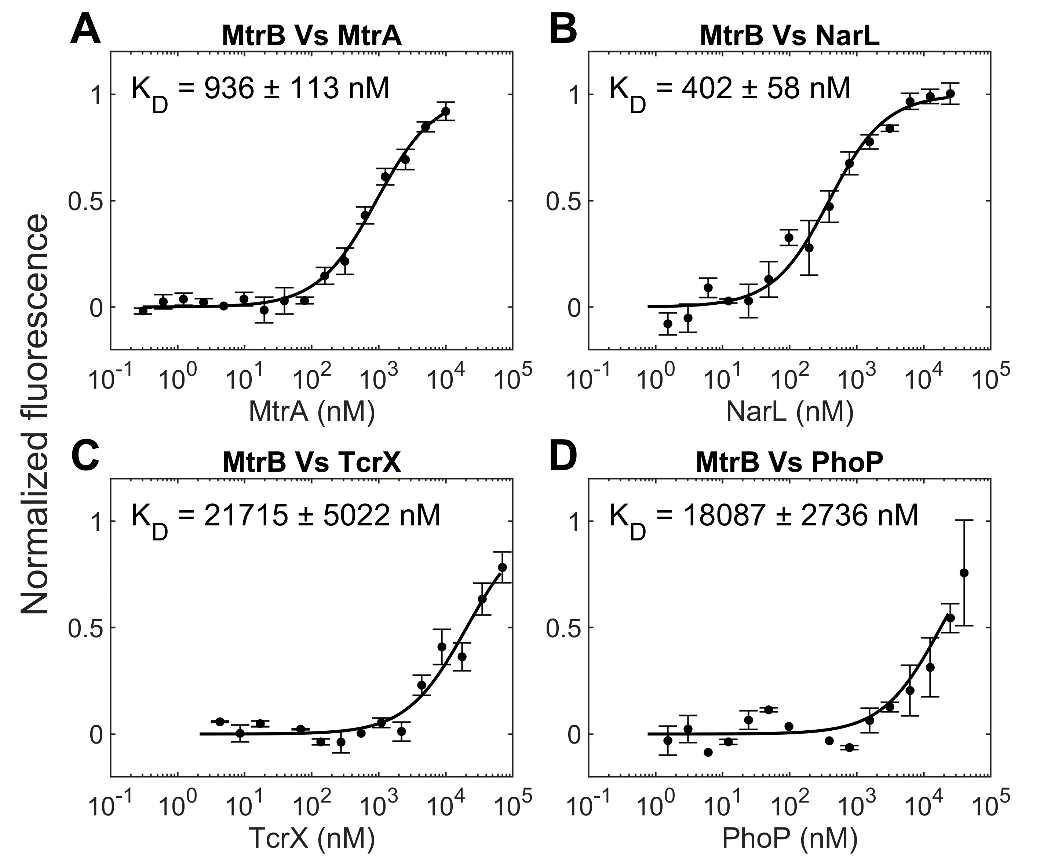
**

**Figure S1. Binding affinities of unphosphorylated MtrB for cognate and non-cognate RRs.** Normalized fluorescence intensity obtained from microscale thermophoresis (see Methods) of 50 nM of fluorescently tagged MtrB, MtrB-GFP, as a function of the concentration of the titrant RR (concentration range): **(A)** MtrA (0.3 nM to 10 μM), **(B)** NarL (1.5 nM to 25 μM), **(C)** PhoP (1.52 nM to 40 μM), and **(D)** TcrX (4.27 nM to 70 μM). Curves are best-fits and symbols are mean ± S.E.M (n =4 independent experiments for MtrB with MtrA and for MtrB with NarL; n=3 independent experiments for the MtrB with TcrX and MtrB with PhoP plots).

**
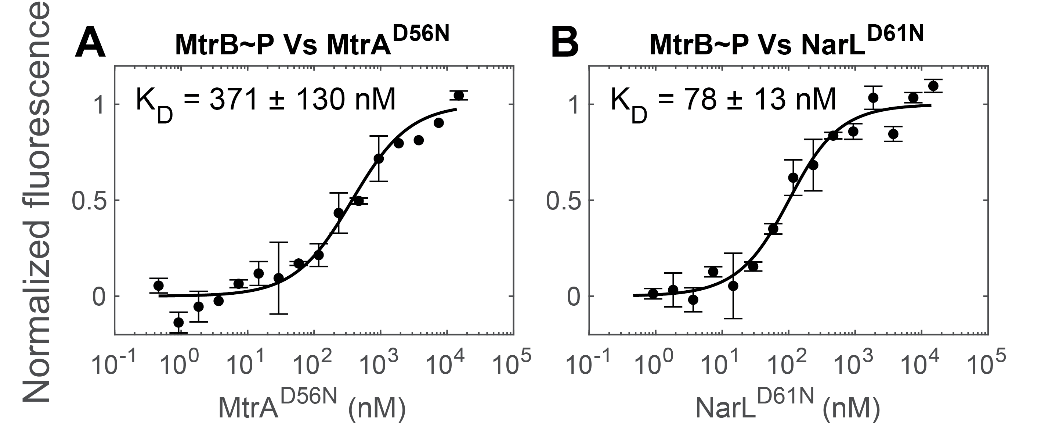
**

**Figure S2. Binding affinities of phosphorylated MtrB for cognate and non-cognate mutant RRs.** Normalized fluorescence intensity obtained from microscale thermophoresis (see Methods) of 50 nM of fluorescently tagged MtrB post autophosphorylation, P~MtrB-GFP, as a function of the concentration of the titrant RR (concentration range): **(A)** MtrA^D56N^ (0.45 to 15 μM), **(B)** NarL^D61N^ (0.45 to 15 μM) The resulting K_D_ values are indicated. Curves are best-fits and symbols are mean ± S.E.M (n =3 independent experiments).


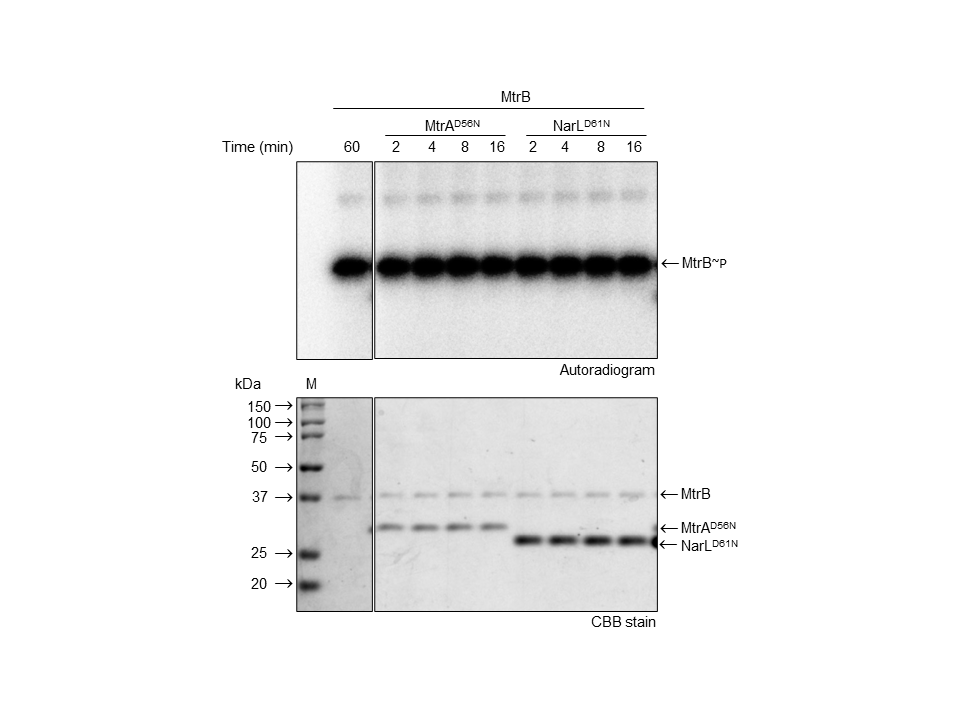


**Figure S3. MtrB~P levels in the presence of cognate and non-cognate RRs over timescales associated with the MST measurements.** Time-course of the levels of MtrB~P in the absence or presence of either MtrA^D65N^ or NarL^D61N^ following coincubation of 50 nM of MtrB post autophosphorylation and 100 nM of MtrA^D56N^ or NarL^D61N^. The top panel is an autoradiogram and the bottom panel is the corresponding Coomassie brilliant blue (CBB) stained image. Of note,the MtrB~P levels remain conserved over these timescales in all cases. The PAGE/Autoradiography image is representative of three independent experiments.


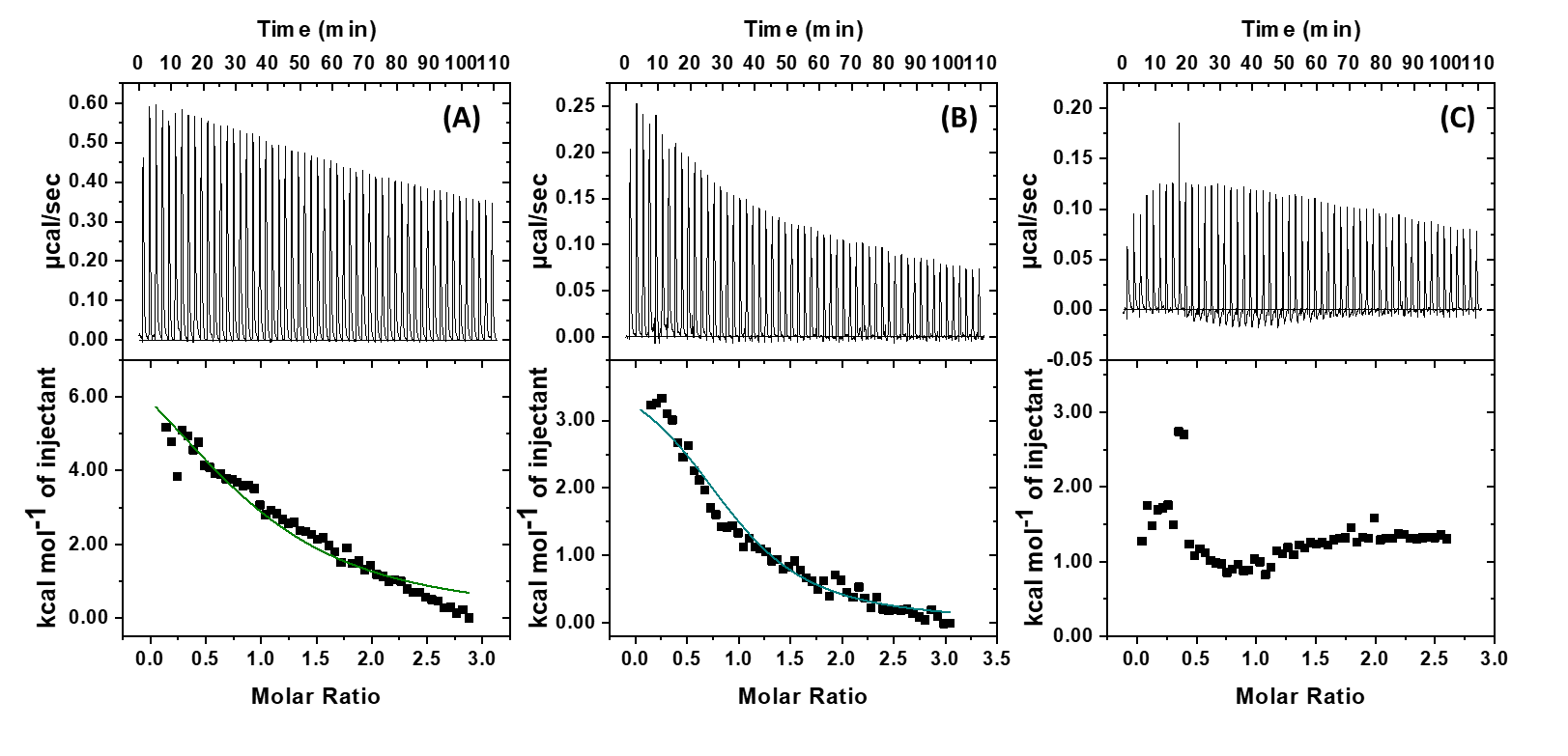


**Figure S4. Isothermal calorimetry measurements for HK~P–RR affinity.** Heat profiles for the interaction of the phosphorylated HK MtrB~P with **(A)** the cognate RR MtrA and the non-cognate RRs **(B)** NarL and **(C)** PdtaR. The top portion of each panel represents baseline corrected thermograms. The bottom portion of each panel shows the corresponding binding isotherms generated by plotting the integrated heat peaks against the molar ratio of the protein.

**
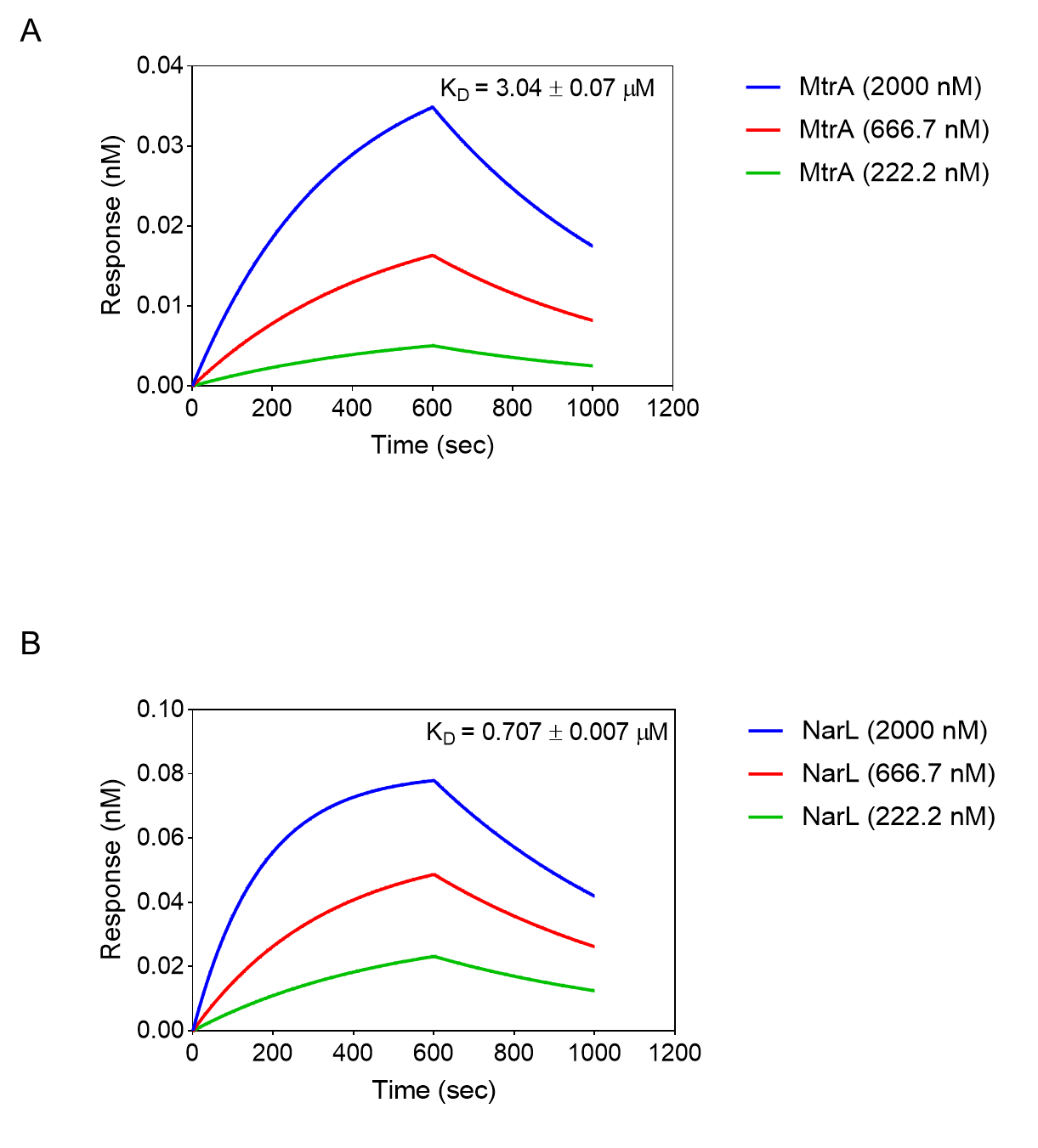
**

**Figure S5. Biolayer interferometry measurements of the affinity of MtrB~P for MtrA and NarL.** Biotinylated MtrB~P immobilized on a streptavidin biosensor surface and titrated against different concentrations (indicated) of (A) MtrA or (B) NarL. The shift in the interference pattern (in nm) measured during the binding and unbinding phases yielded estimates of the binding affinities (Methods). MtrB~P had 4.3-fold higher affinity for NarL than MtrA.


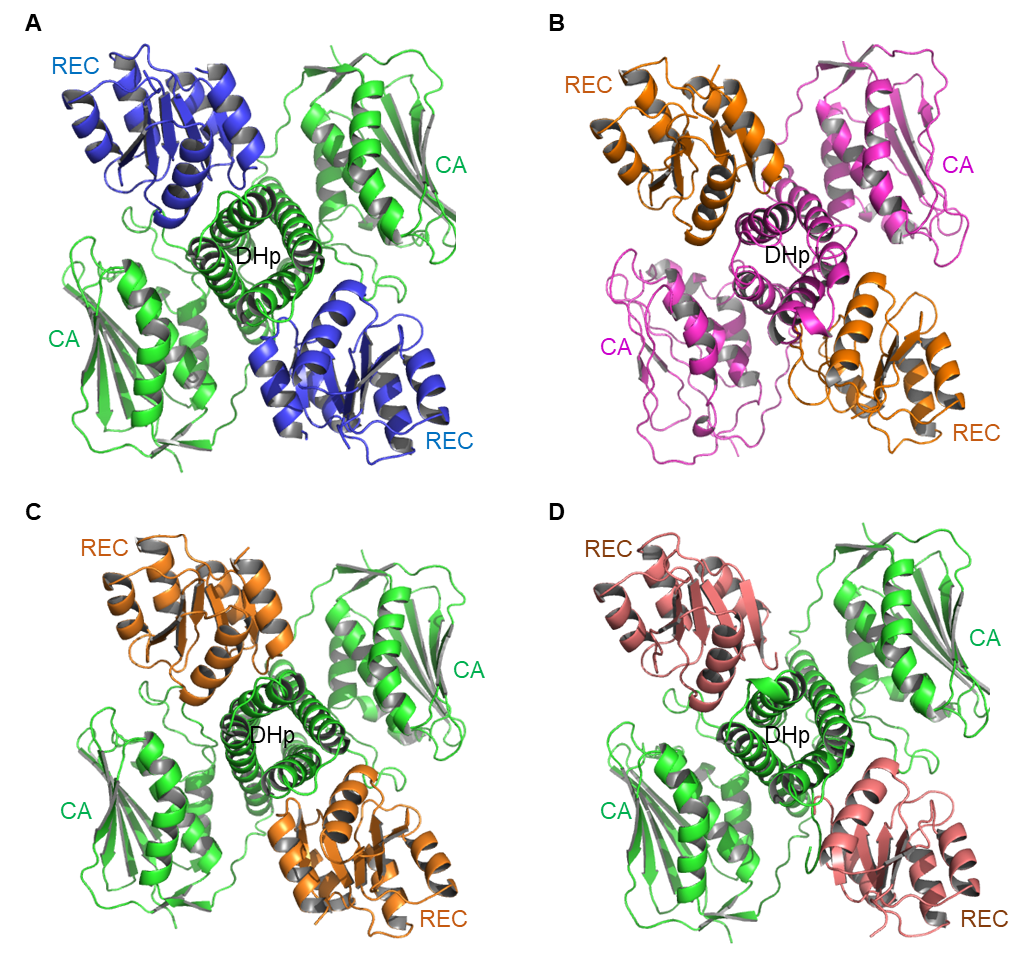


**Figure S6. Structural models of HK:RR pairs built using homology modelling. (A)** Model of MtrB (green) in complex with its cognate RR MtrA (blue). **(B)** Model of NarS (pink) in complex with its cognate RR NarL (orange). **(C)** Model of MtrB (green) in complex with the non-cognate RR NarL (orange). **(D)** Model of MtrB (green) in complex with the non-cognate RR PdtaR (red). **DHp:** dimerization and histidine phosphotransfer domain; **CA:** catalytic domain; **REC:** receiver domain of RR.


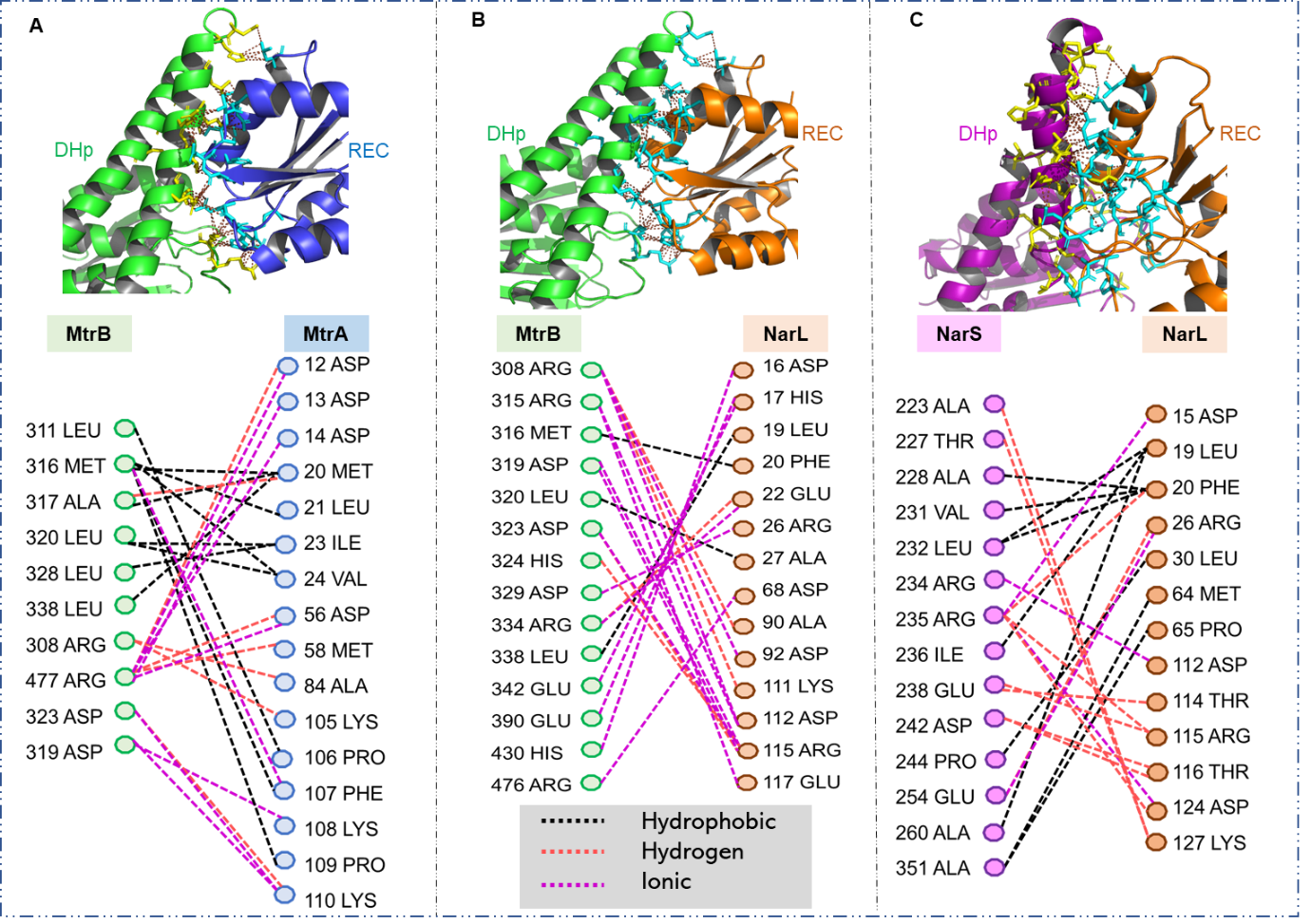


**Figure S7.** **Interface analysis of HK:RR complexes.** (A) A cartoon representation of the HK MtrB (green) in interaction with its cognate RR MtrA (blue) where interfacial residues are shown in stick representation and the interactions are highlighted in brown dotted lines. Below is a pictorial representation of the interactions between MtrB and MtrA. (B) A cartoon representation of the HK MtrB (green) in interaction with the non-cognate RR NarL (orange) where interfacial residues are shown in stick representation and the interactions are highlighted in brown dotted lines. Below is a pictorial representation of interactions between MtrB and NarL. (C) A cartoon representation of the HK NarS (pink) in interaction with its cognate RR NarL (orange) where interfacial residues are shown in stick representation and the interactions are highlighted in red dotted lines. Below is a pictorial representation of the interactions between NarS and NarL. In the pictorial representations, a dotted black line indicates a hydrophobic interaction, a dotted red line indicates hydrogen bonding, and a dotted pink line indicates ionic interaction. DHp: dimerization and histidine phosphotransfer domain of HK; REC: receiver domain of RR***.***

**
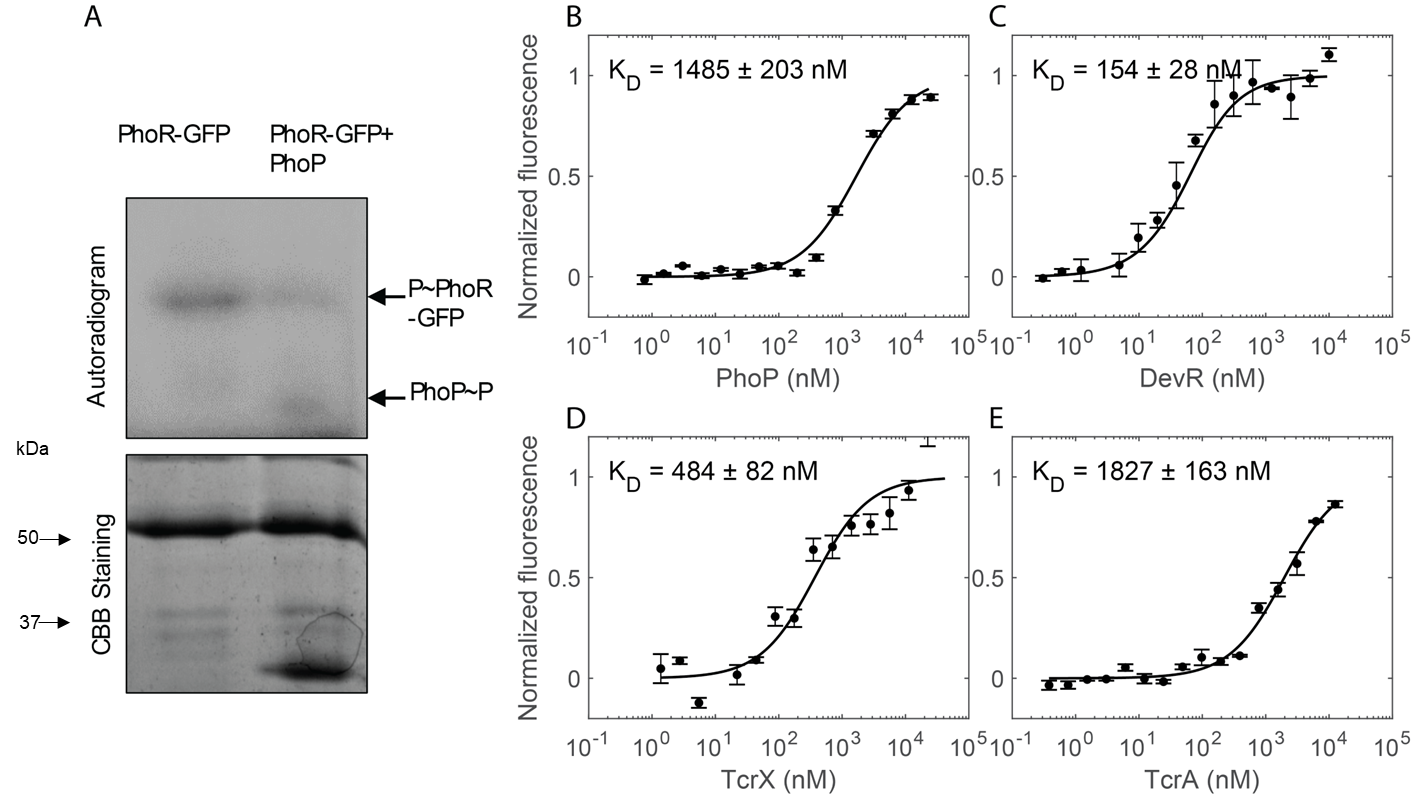
**

**Figure S8. Functional activity assay and binding affinities of phosphorylated PhoR-GFP for cognate and non-cognate RRs. (A)** Autophosphorylation of the phosphorylated HK PhoR-GFP with subsequent phosphotransfer to the cognate RR PhoP. **(B-E)** Normalized fluorescence intensity obtained from microscale thermophoresis (see Methods) of 50 nM of P~PhoR-GFP as a function of the concentration of the titrant RR (concentration range): (**B)** PhoP (0.73 nM to 25 μM), **(C)** DevR (0.31 nM to 10 μM), **(D)** TcrX (1.37 nM to 22.5 μM), (**E)** TcrA (0.38 nM to 12.5 μM). The resulting K_D_ values are indicated. Symbols are mean ± S.E.M. from n=3 independent experiments and curves are best-fits.

**
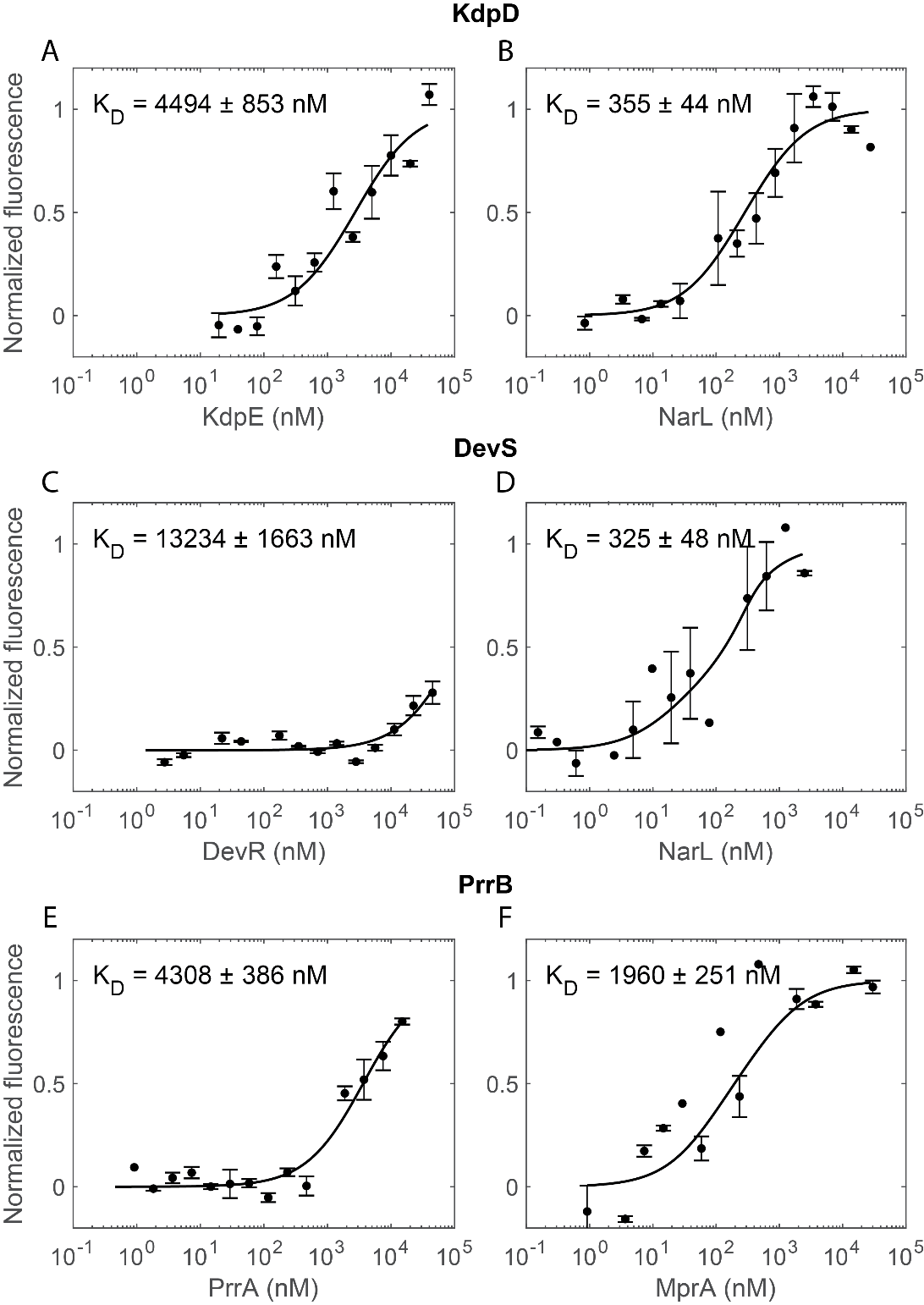
**

**Figure S9. Binding affinities of phosphorylated HKs for cognate and non-cognate RRs.** Normalized fluorescence intensity obtained from microscale thermophoresis (see Methods) of 50 nM of fluorescently tagged HK post autophosphorylation as a function of the concentration of the titrant RR. The HKs are indicated as headers and the RRs as x-axis labels. The titrant concentration ranges used are as follows: **(A)**  KdpE (19.5 nM to 40 μM), **(B)**  NarL (0.84 nM to 27.5 μM), **(C)** DevR (2.75 nM to 45 μM), **(D)** NarL (0.15 nM to 5 μM), **(E)** PrrA (0.92 nM to 15 μM), and **(F)** MprA (0.92 nM to 30 μM). The resulting K_D_ values are indicated. Curves are best-fits and symbols are mean ± S.E.M (n =4 independent experiments for KdpD~P with KdpE and for DevS~P with DevR; n=3 independent experiments for the remaining plots).


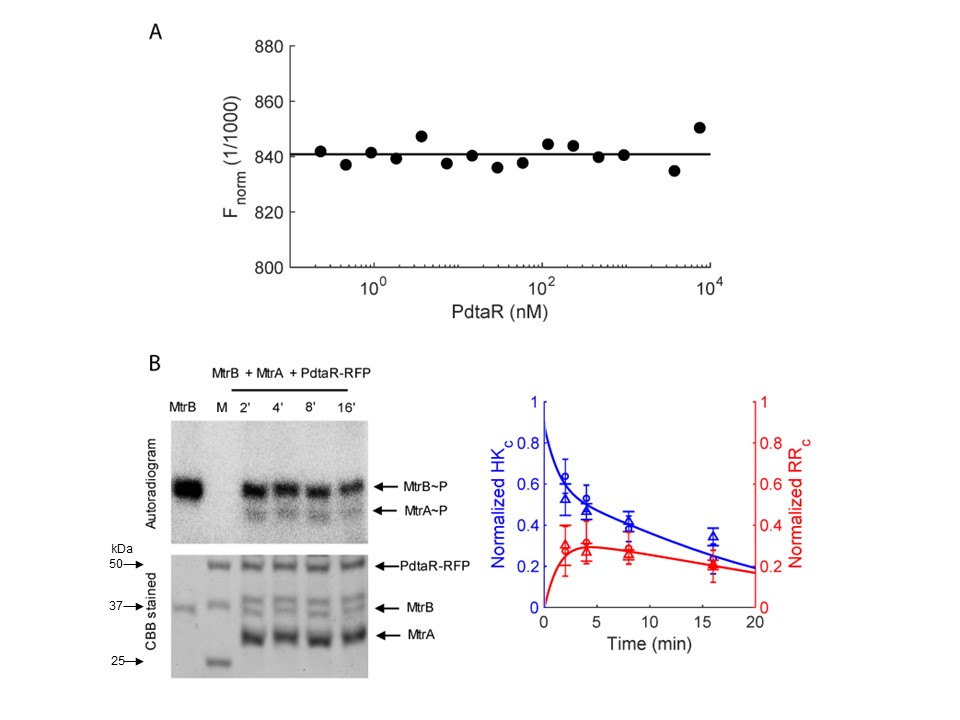


**Figure S10. Phosphotransfer kinetics from MtrB to MtrA with non-binding PdtaR-RFP. (A)** Representative thermophoretic change profile of 50 nM P~MtrB-GFP with the titrant RR PdtaR (0.23 nM to 7.5 μM), indicating no binding. **(B)** Time course assay of the phosphotransfer from MtrB~P to the cognate RR MtrA (100 pmol) in the presence of the non-cognate RR PdtaR-RFP (left) and its densitometric analysis, performed using autoradiograph band intensities normalized by the same band in the CBB stained gel (right). Top panels are autoradiograms and bottom panels corresponding Coomassie Brilliant Blue (CBB) stained gels (left). The autophosphorylation control was used to normalize the intensities of the individual bands. Blue symbols represent MtrB~P and red symbols MtrA~P. Lines represent best-fits of our model (Methods). The error bars represent mean ± S.E.M (n=3).


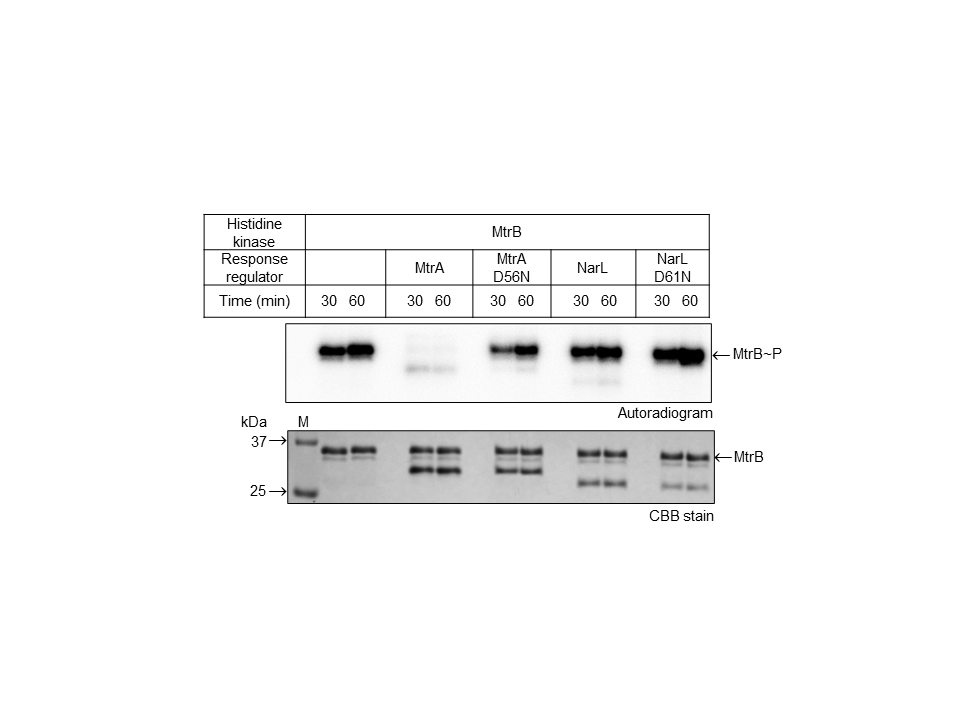


**Figure S11. Autophosphorylation of MtrB in the presence of cognate and non-cognate RRs.** Autophosphorylation time-course analysis of the HK MtrB in the absence of any RR or in the presence of the cognate RR MtrA, the non-cognate RR NarL, or the phosphotransfer defective mutant RRs MtrA^D56N^ or NarL^D61N^. The top panel has autoradiograms and the bottom panel the corresponding Coomassie brilliant blue (CBB) stained images. The substantial decay of MtrB~P in the presence of wildtype MtrA compared to the other RRs is noteworthy. The PAGE/Autoradiography image is representative of three independent experiments.


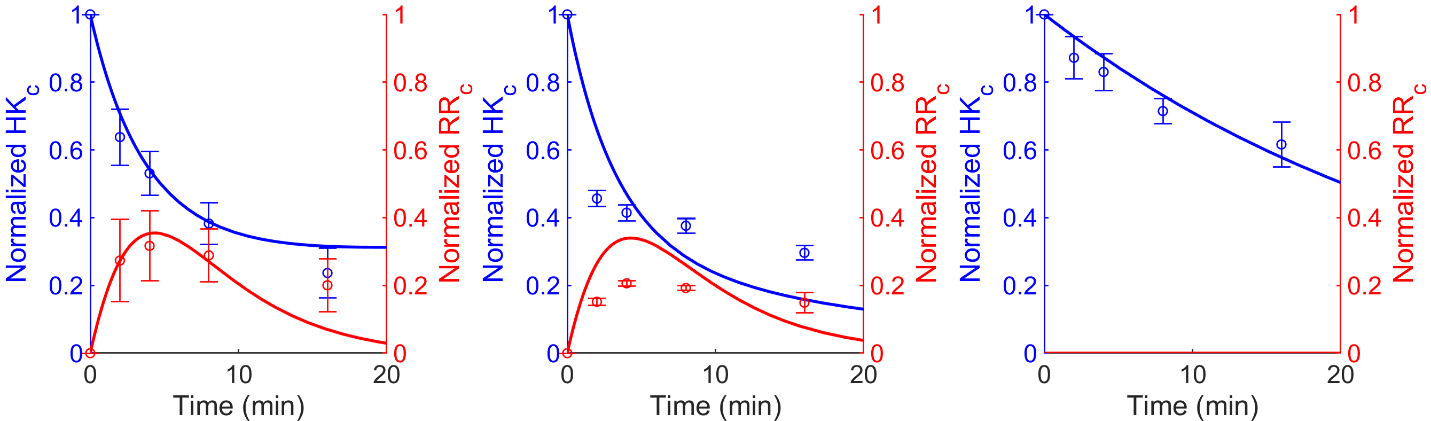


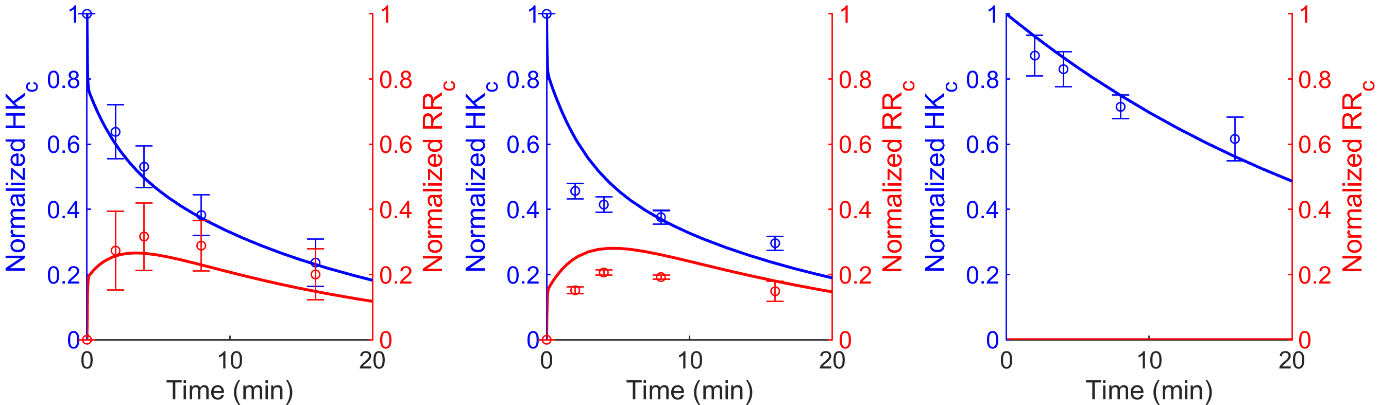


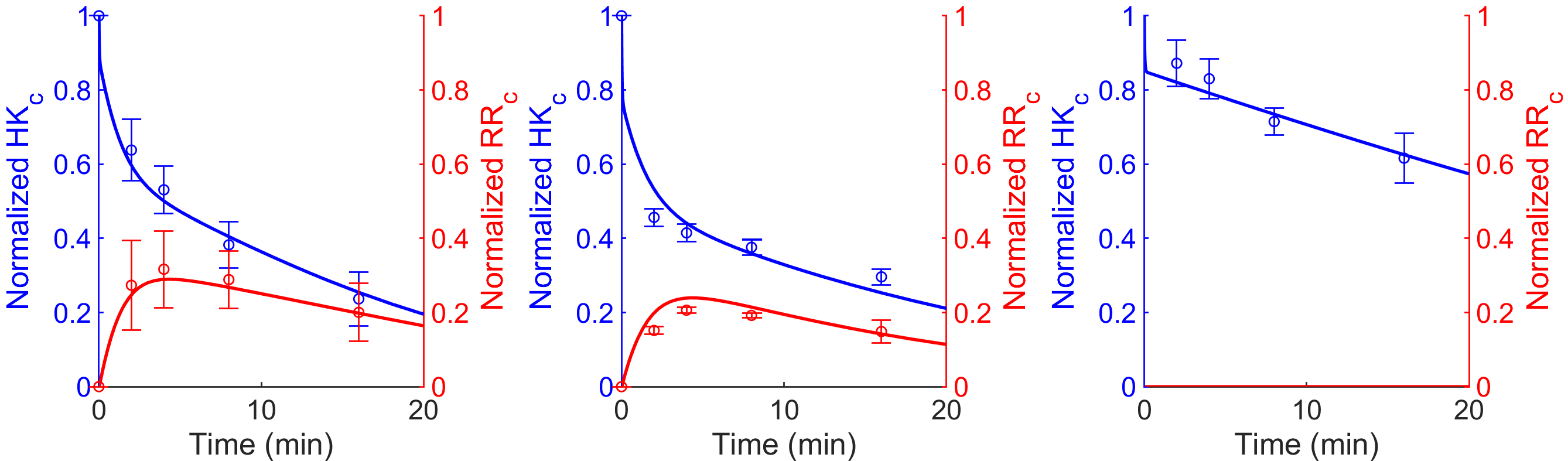


**Figure S12. Model selection and fits to data.** Fits (lines) of the different models (see Note S2) to the data (symbols) in Figure 3. *Top row*: Fit of Model 1. Best-fit parameter estimates (95% confidence intervals) are $k_{p}^{RR}=2.1\times10^{-4} (1.1\times10^{-4}-4.2\times10^{-4});$ $k_{dp}^{RR}=4.7\times10^{-4} (2.9\times10^{-4}-7.8\times10^{-4});$ $k_{dp}^{RR-nc}=3.6\times10^{-5} (1.9\times10^{-5}-6.9\times10^{-5});$ and. *Middle row*: Fit of Model 2. Best-fit parameter estimates (95% confidence intervals) are $k_{p}^{RR}=34.98(0.19-6346);$ $k_{dp}^{RR}=1.1\times10^{-4} (1.6\times10^{-5}-8.2\times10^{-4}$ $k_{dc}^{RR}=6.01 (0.23-155.6);$ $k_{dc}^{RR-nc}=0.036 (0.026-0.050);$ and . *Bottom row*: Fit of Model 3. Best-fit parameter estimates (95% confidence intervals) are listed in Table S4. The fit of model 3 is reproduced in Figure 3. The error bars represent mean ± S.E.M (n=3).

**
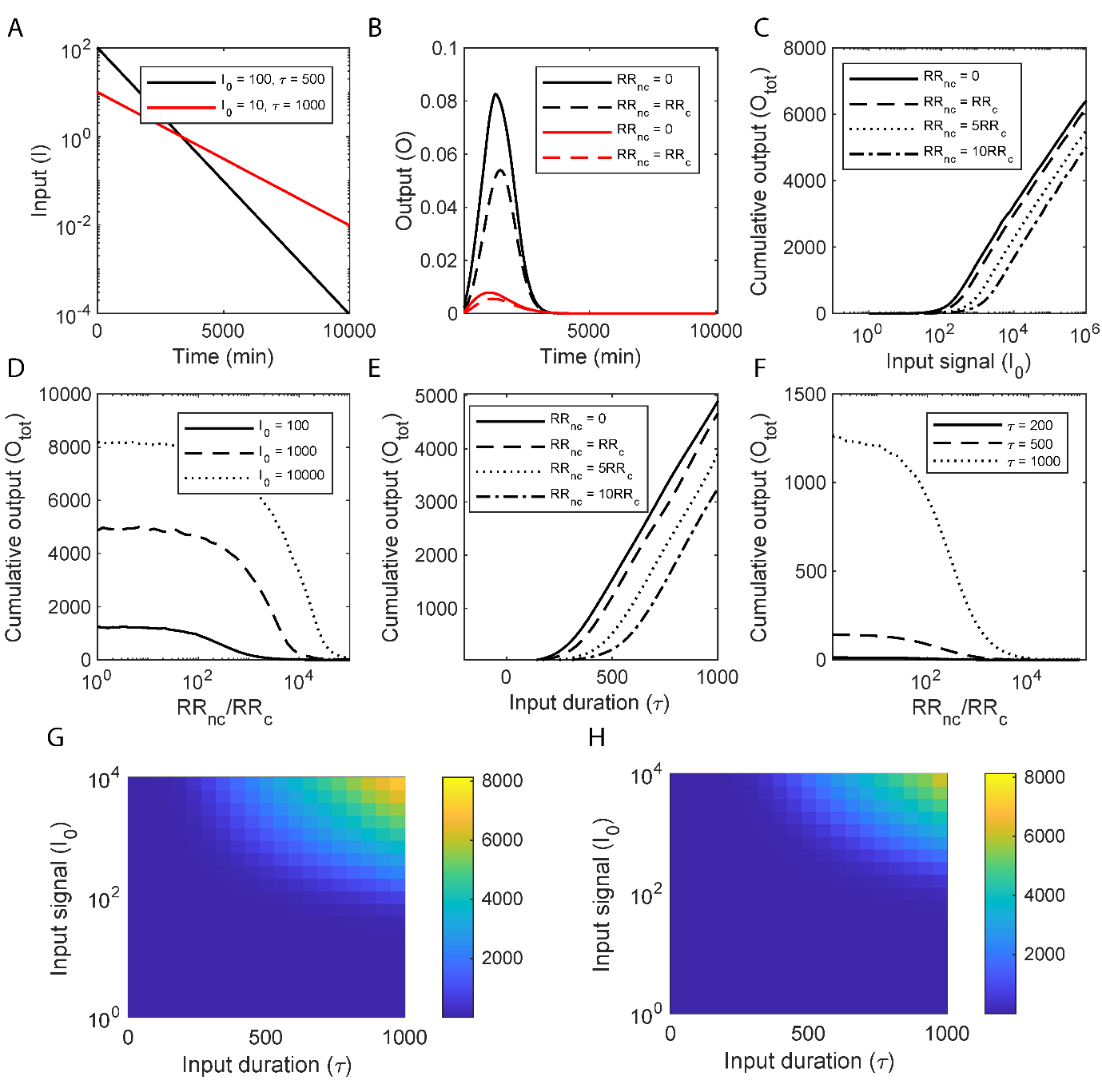
**

**Figure S13. Model predictions of the total TCS signal response (***O_total_***) and the impact of sequestration.** **(A)** Representative inputs, *I*, indicating strong but short-lived (black) and weak but extended (red) stimuli. **(B)** The corresponding outputs without (solid lines) and with (dashed lines) sequestration by a non-cognate RR. **(C)** The total response (*O_total_*) as a function of the maximum input, *I*_0_, for different extents of sequestration, determined by the ratios of the non-cognate RRs, RR­_nc_ to the cognate RR, RR_c_, indicated. **(D)** *O_total_* as a function of the ratio RR­_nc_/RR_c_ for different *I*_0_. **(E)** *O_total_* as a function of the signal half-life, *τ*, for different values of RR­_nc_/RR_c_. **(F)** *O_total_* as a function of RR­_nc_/RR_c_ for different values of *τ*. (*τ* is in minutes throughout.) Heatmaps showing *O_total_* as functions of *I*_0_ and *τ* in the **(G)** absence or **(H)** presence of non-cognate RRs, indicating the threshold stimulation for response shifting to higher *I*_0_ and *τ* with sequestration. Corresponding calculations for the peak response, *O_max_*, are in Figure 5. Model predictions were obtained by solving Eqs. (1)-(47) (Methods) using parameter values listed in Table S4.

**
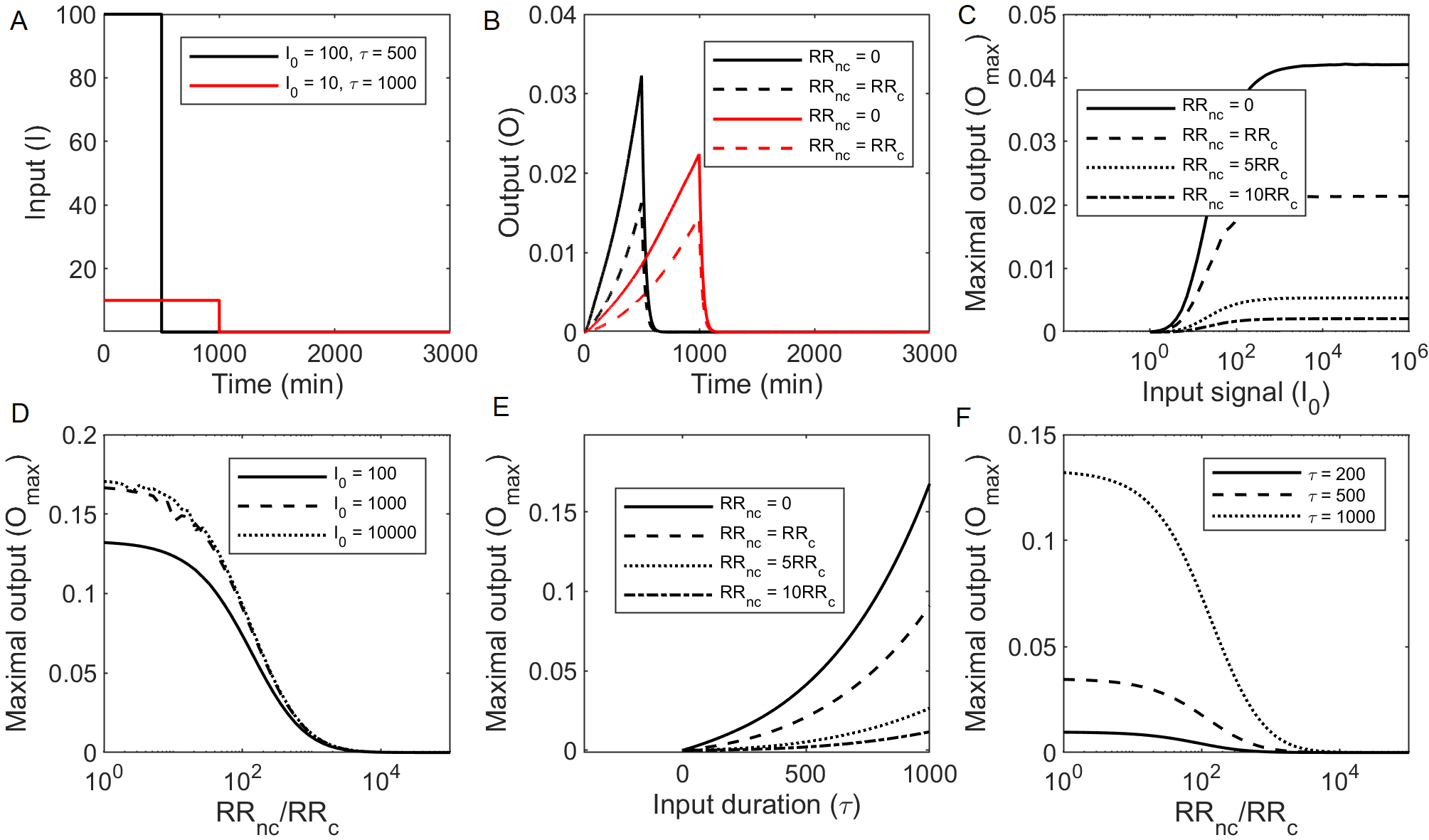
**

**Figure S14. Model predictions of TCS signal transduction and the impact of sequestration for step inputs.** **(A)** Representative inputs, *I*, indicating strong but short-lived (black) and weak but longer (red) stimuli. **(B)** The corresponding outputs without (solid lines) and with (dashed lines) sequestration by a non-cognate RR. **(C)** The peak of the response (*O_max_*) as a function of the maximum input, *I*_0_, for different extents of sequestration, determined by the ratios of the non-cognate RRs, RR­_nc_, to the cognate RR, RR_c_, indicated. **(D)** *O_max_* as a function of the ratio RR_nc_/RR_c_ for different *I*_0_. **(E)** *O_max_* as a function of the signal half-life, *τ*, for different values of RR­_nc_/RR_c_. **(F)** *O_max_* as a function of RR­_nc_/RR_c_ for different values of *τ*. (*τ* is in minutes throughout.) Model predictions were obtained by solving Eqs. (1)-(47) (Methods) using parameter values listed in Table S4.


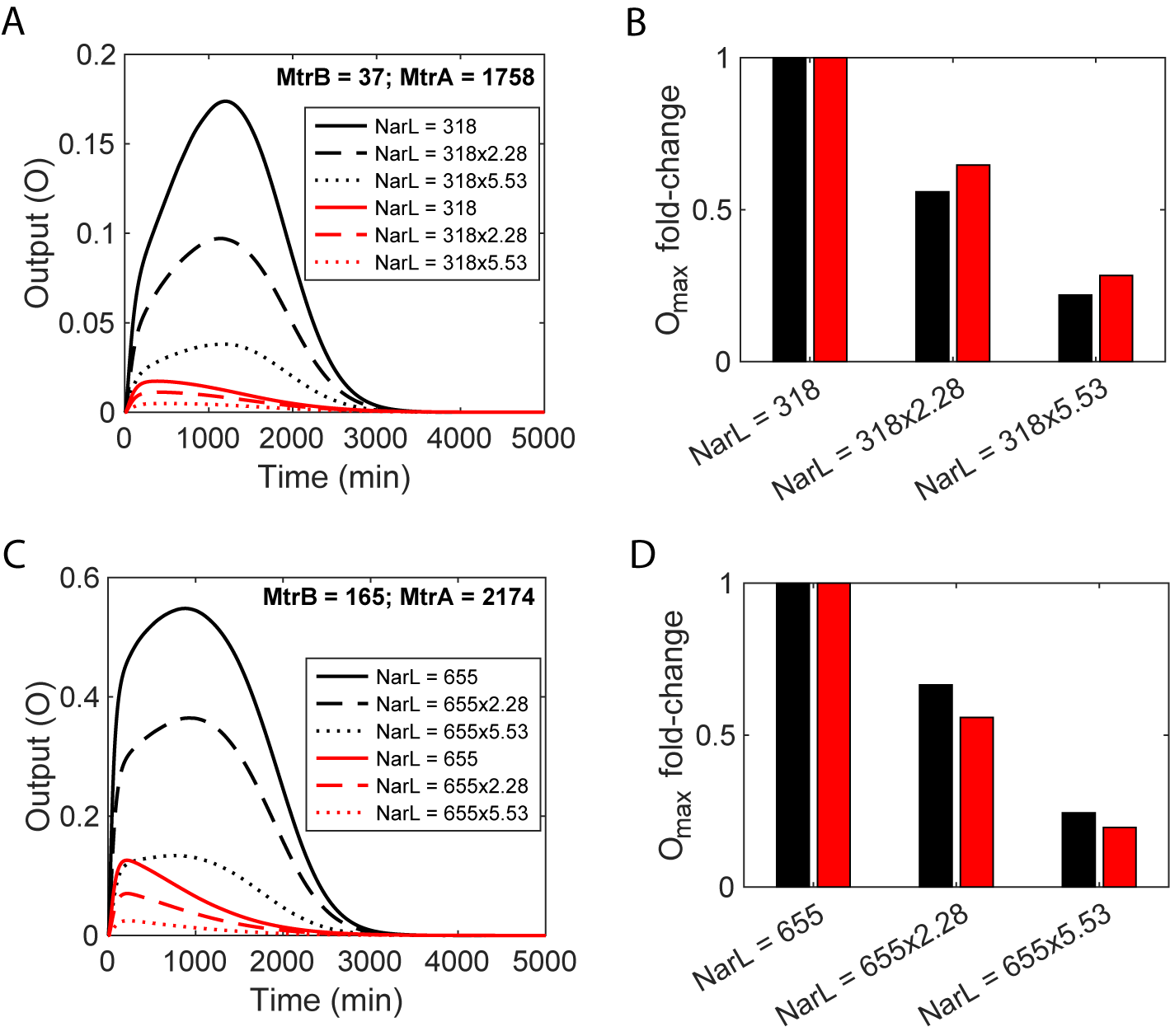


**Figure S15. Model predictions are consistent with the observations in vivo.** To recapitulate the observations in vivo (Figure 7), we performed model calculations using parameter settings representative of the in vivo scenario. Specifically, we used protein levels reported recently^7^ that are representative of **(A, B)** *M. tuberculosis H37Rv* (MtrA: 1758; MtrB: 37; NarL: 318 copies/cell) and **(C, D)** *M. bovis* (MtrA: 2174; MtrB: 165; NarL: 655 copies/cell). We used the binding affinities of the proteins we measured in the present study (Figures 1 and S1). All other parameters were the same as in Figure 5. We also let the stimuli be the same as in Figure 5 (I_0_=100 and τ=500 min (black); I_0_=10 and τ=1000 min (red)) and computed the output, O, as the promoter bound phosphorylated MtrA (Methods). We then performed calculations by increasing the level of the non-cognate RR, NarL, by amounts corresponding to the fold-changes realized using aTC induction in vivo, namely 2.3-fold and 5.5-fold with 10 ng/µL and 50 ng/µL aTC induction, respectively (Figure 7). **(A)** Time course of the output, and **(B)** fold-change in the peak output (O_max_) relative to the peak with no aTC induction for the *M. tuberculosis H37Rv* parameters. **(C, D)** Corresponding predictions with the *M. bovis* parameters. Note that the reduction of ~40% with 10 ng/µL aTC is consistent with our *in vivo* observations (mean 25% and range 10-45%; Figure 7). The predicted reduction of ~70% with 50 ng/µL aTC is larger than corresponding mean observations (~30%), but lies within the range observed (10-70%; Figure 7). These comparisons are remarkable given the lack of knowledge of the stimulus and that the measurements may not be precisely at the peak output.

**SUPPLEMENTARY REFERENCES**

1. Agrawal R, Pandey A, Rajankar MP, Dixit NM, Saini DK. The two-component signalling networks of *Mycobacterium tuberculosis* display extensive cross-talk in vitro. *Biochem J* **469**, 121-134 (2015).

2. Sankhe GD, Dixit NM, Saini DK. Activation of bacterial histidine kinases: Insights into the kinetics of the cis autophosphorylation mechanism. *mSphere* **3**, (2018).

3. Rowland MA, Deeds EJ. Crosstalk and the evolution of specificity in two-component signaling. *Proc Natl Acad Sci U S A* **111**, 5550-5555 (2014).

4. Gao R, Stock AM. Probing kinase and phosphatase activities of two-component systems in vivo with concentration-dependent phosphorylation profiling. *Proc Natl Acad Sci U S A* **110**, 672-677 (2013).

5. Tiwari A, Balazsi G, Gennaro ML, Igoshin OA. The interplay of multiple feedback loops with post-translational kinetics results in bistability of mycobacterial stress response. *Phys Biol* **7**, 036005 (2010).

6. Nath K, Koch AL. Protein degradation in Escherichia coli. II. Strain differences in the degradation of protein and nucleic acid resulting from starvation. *J Biol Chem* **246**, 6956-6967 (1971).

7. Schubert OT, Ludwig C, Kogadeev M, Zimmerman M, Rosenberger G, *et al*. Absolute proteome composition and dynamics during dormancy and resuscitation of Mycobacterium tuberculosis *Cell Host Microbe* **18**, 96-108 (2015).
