# Supplementary material for: Sequestration of histidine kinases by non-cognate response regulators establishes a threshold level of stimulation for bacterial two-component signaling
Source: Nat Commun. 2023 Jul 25;14:4483. doi: 10.1038/s41467-023-40095-2 (PMC10368727; doi:10.1038/s41467-023-40095-2)
Supplement: Supplementary file 2 — Reporting Summary [file 41467_2023_40095_MOESM2_ESM.pdf]

nature portfolio

Corresponding author(s): Deepak Kumar Saini, Narendra M. Dixit

Last updated by author(s): Jul 11, 2023

Reporting Summary

Nature Portfolio wishes to improve the reproducibility of the work that we publish. This form provides structure for consistency and transparency in reporting. For further information on Nature Portfolio policies, see our Editorial Policies and the Editorial Policy Checklist.

Statistics

For all statistical analyses, confirm that the following items are present in the figure legend, table legend, main text, or Methods section.

n/a	Confirmed	
☐	☒	The exact sample size (*n*) for each experimental group/condition, given as a discrete number and unit of measurement
☐	☒	A statement on whether measurements were taken from distinct samples or whether the same sample was measured repeatedly
☐	☒	The statistical test(s) used AND whether they are one- or two-sided<br>*Only common tests should be described solely by name; describe more complex techniques in the Methods section.*
☒	☐	A description of all covariates tested
☒	☐	A description of any assumptions or corrections, such as tests of normality and adjustment for multiple comparisons
☐	☒	A full description of the statistical parameters including central tendency (e.g. means) or other basic estimates (e.g. regression coefficient) AND variation (e.g. standard deviation) or associated estimates of uncertainty (e.g. confidence intervals)
☐	☒	For null hypothesis testing, the test statistic (e.g. F, t, r) with confidence intervals, effect sizes, degrees of freedom and P value noted<br>*Give P values as exact values whenever suitable.*
☒	☐	For Bayesian analysis, information on the choice of priors and Markov chain Monte Carlo settings
☒	☐	For hierarchical and complex designs, identification of the appropriate level for tests and full reporting of outcomes
☒	☐	Estimates of effect sizes (e.g. Cohen's *d*, Pearson's *r*), indicating how they were calculated

Our web collection on statistics for biologists contains articles on many of the points above.

Software and code

Policy information about availability of computer code

Data collection	Typhoon 9210 phosphorimager (GE Healthcare, USA), Azure Sapphire (Azure Biosystems, USA) for autoradiography, Monolith NT.115 (NanoTemper Technologies Germany) for MST data collection, NanoDrop® ND-1000 UV-Vis Spectrophotometer (NanoDrop Technologies, USA) to quantify RNA yield, QIAquant 96 5plex(Qiagen, USA) for RT-PCR analysis, MicroCal VP-ITC calorimeter for isothermal titration calorimetry (ITC) experiment, OctetRED96 for bio-layer interferometry (BLI).
Data analysis	PSI-blast for sequence alignment, MODELLER v9.12 for structural model, SCRWL 4.0 library for side-chain conformations, GROMACS (v5.1) for energy minimization, FoldX (4.0) Repair PDB to obtain a stable complex, PROCHECK53 and MolProbity to check stereochemical quality, Protein interaction calculator (PIC) to check nature of interaction, PyMol to visualize Interface regions, MO. Control and MO.Affinity Analysis software (NanoTemper Technologies Germany) for MST data collection and analysis respectively, QIAquant 96 software (Qiagen, USA) for RT-PCR data analysis, Origin 7.0 software for fitting ITC data, GraphPad Prism 8.4.2 for BLI data fitting and representation, Sapphire Capture and AzureSpot software for for phosphor imaging and analysis respectively, ImageJ software for autoradiography data quantification. A Custom code used for mathematical modeling is available on GitHub (https://github.com/rubeshr1991/tcs_project.git).

For manuscripts utilizing custom algorithms or software that are central to the research but not yet described in published literature, software must be made available to editors and reviewers. We strongly encourage code deposition in a community repository (e.g. GitHub). See the Nature Portfolio guidelines for submitting code & software for further information.

Data

Policy information about availability of data

All manuscripts must include a data availability statement. This statement should provide the following information, where applicable:

- Accession codes, unique identifiers, or web links for publicly available datasets
- A description of any restrictions on data availability
- For clinical datasets or third party data, please ensure that the statement adheres to our policy

For cognate and noncognate SK:RR complex analysis, we have used MtrB- “P9WGK9[https://www.uniprot.org/uniprotkb/P9WGK9/entry]”; MtrA- “P9WGM7[https://www.uniprot.org/uniprotkb/P9WGM7/entry]”; NarS- “O53857[https://www.uniprot.org/uniprotkb/O53857/entry]”; NarL- “P9WGM5[https://www.uniprot.org/uniprotkb/P9WGM5/entry]”; PdtaR- “P9WGM3[https://www.uniprot.org/uniprotkb/P9WGM3/entry]”, A template (PDB ID: 5UHT) used for modeling selected TCS SK:RR complexes. All data generated in this study are presented in the Figures and Tables. Source data are provided with this paper. The computer code used to implement mathematical modelling is available on GitHub. (https://github.com/rubeshr1991/tcs_project.git) indexed at Zenodo database as https://doi.org/10.5281/zenodo.8130204. The experimental data generated in this study has been deposited in the Zenodo database under accession code 115507 ((https://doi.org/10.5281/zenodo.8132755) as a consolidated source file and raw images files along with BLI data.

Human research participants

Policy information about studies involving human research participants and Sex and Gender in Research.

Reporting on sex and gender	Not applicable in the present study.
Population characteristics	Not applicable in the present study.
Recruitment	Not applicable in the present study.
Ethics oversight	Not applicable in the present study.

Note that full information on the approval of the study protocol must also be provided in the manuscript.

Field-specific reporting

Please select the one below that is the best fit for your research. If you are not sure, read the appropriate sections before making your selection.

☒ Life sciences ☐ Behavioural & social sciences ☐ Ecological, evolutionary & environmental sciences

For a reference copy of the document with all sections, see nature.com/documents/nr-reporting-summary-flat.pdf

Life sciences study design

All studies must disclose on these points even when the disclosure is negative.

Sample size	No statistical methods were used to pre-determine sample size. All the in vitro and in vivo experiment were performed in three or more biologically independent repetitions mentioned in figure legends to ascertain reproducibility and calculate standard statistical parameters. Standard statistical parameters like p values guided the sample space to assert the evaluated statistical significance.
Data exclusions	No data were excluded from the analysis.
Replication	For all the observations presented in this manuscript at least 3 independent experiments were performed to check reproducibility of the experimental findings. All the data of this manuscript were reproducible and has been represented with the statistical significance based on the P-value mentioned as required.
Randomization	Randomization is not relevant in because all mutants and experimental conditions used in each experiment were treated equally and all the in vitro data obtained were in deterministic regimes.
Blinding	The investigators of this study were performed the allocated experiments with no prior knowledge about the experimental outcome.

Reporting for specific materials, systems and methods

We require information from authors about some types of materials, experimental systems and methods used in many studies. Here, indicate whether each material, system or method listed is relevant to your study. If you are not sure if a list item applies to your research, read the appropriate section before selecting a response.

Materials & experimental systems

n/a	Involved in the study
☒	☐ Antibodies
☒	☐ Eukaryotic cell lines
☒	☐ Palaeontology and archaeology
☒	☐ Animals and other organisms
☒	☐ Clinical data
☒	☐ Dual use research of concern

Methods

n/a	Involved in the study
☒	☐ ChIP-seq
☒	☐ Flow cytometry
☒	☐ MRI-based neuroimaging
