# Supplementary material for: Sequestration of histidine kinases by non-cognate response regulators establishes a threshold level of stimulation for bacterial two-component signaling
Source: Nat Commun. 2023 Jul 25;14:4483. doi: 10.1038/s41467-023-40095-2 (PMC10368727; doi:10.1038/s41467-023-40095-2)
Supplement: Supplementary file 3 — Source Data [file 41467_2023_40095_MOESM3_ESM.zip › Source Data/Source Data/Raw gel blots Data images .pdf]

Result presented showing (edited) and unedited/uncropped raw images

Figure 3A

Presented figure

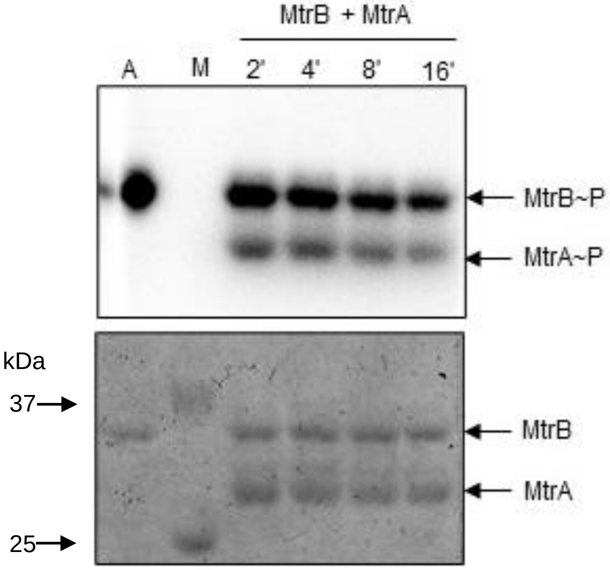

Unedited/uncropped image

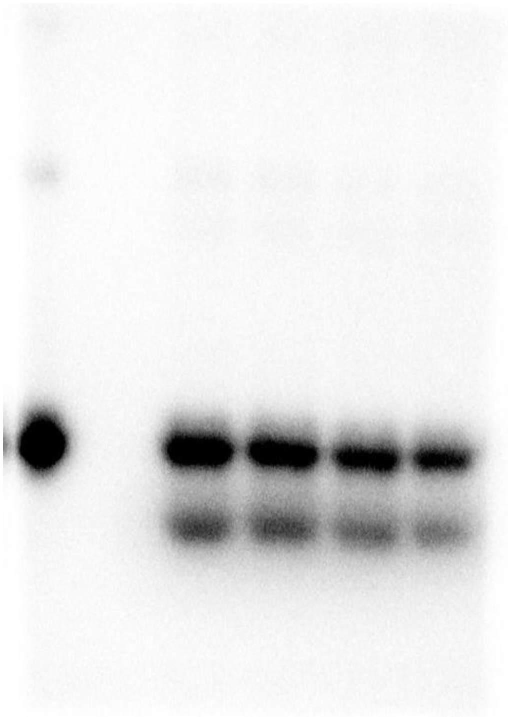

Autoradiogram

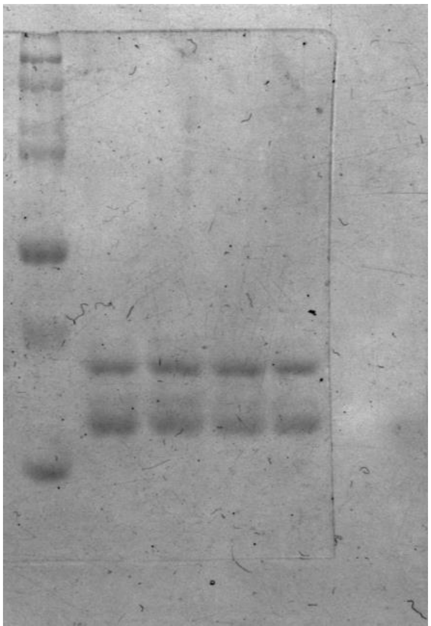

CBB

Figure 3B

Presented figure

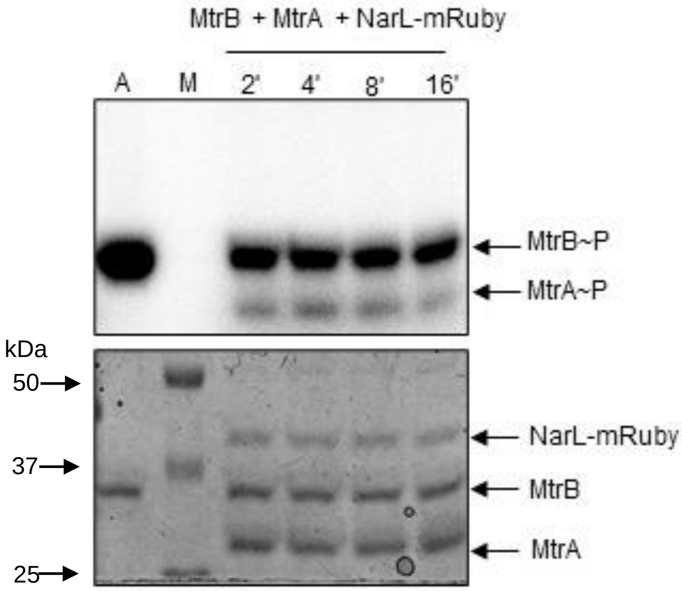

Unedited/uncropped image

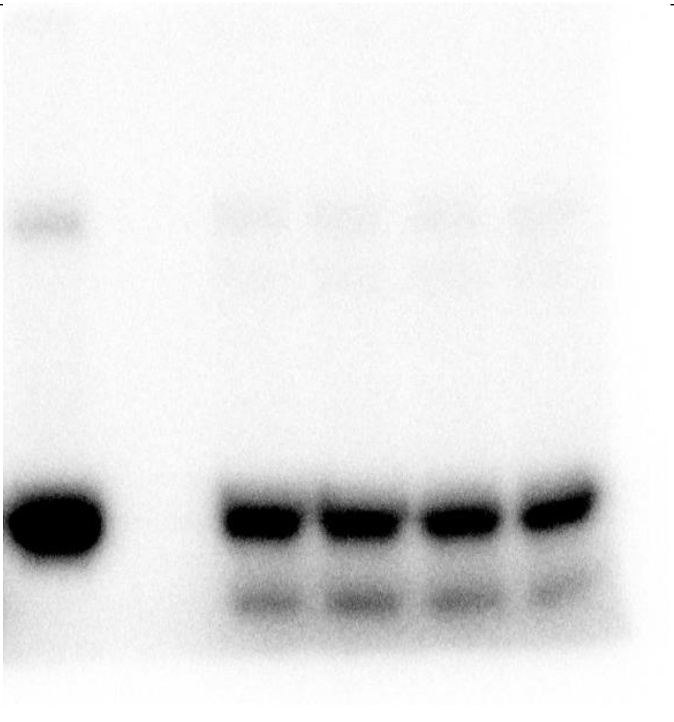

Autoradiogram

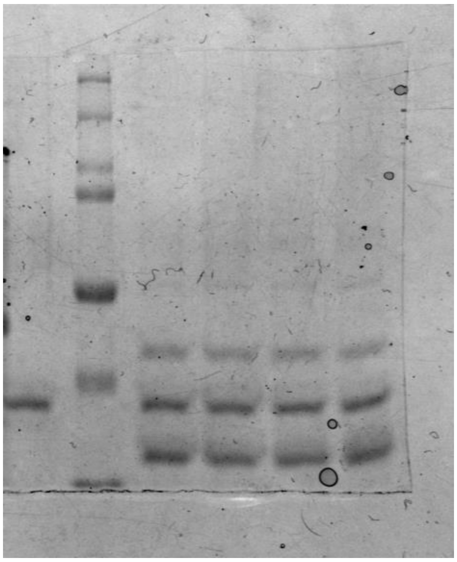

CBB

Figure 3C

Presented figure

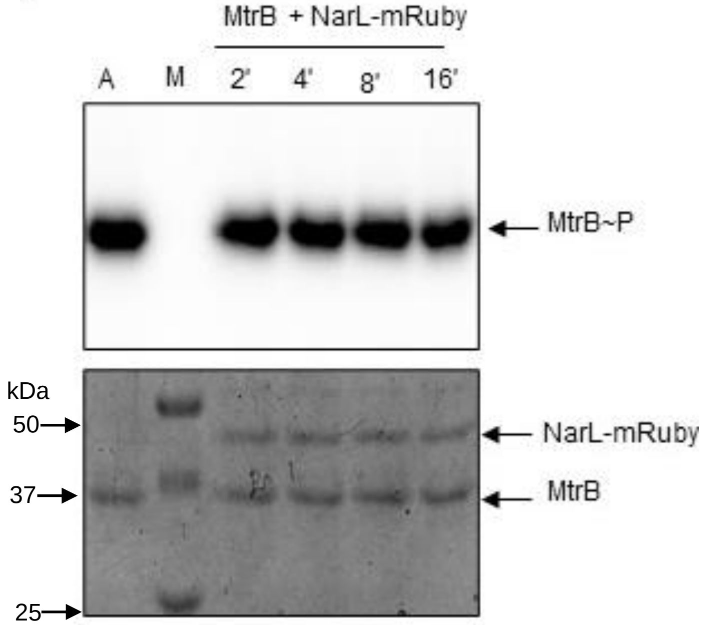

Unedited/uncropped image

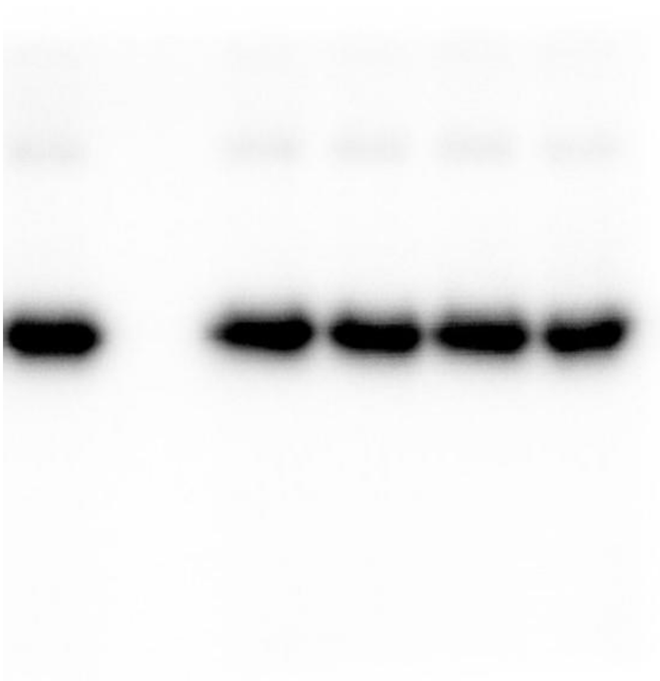

Autoradiogram

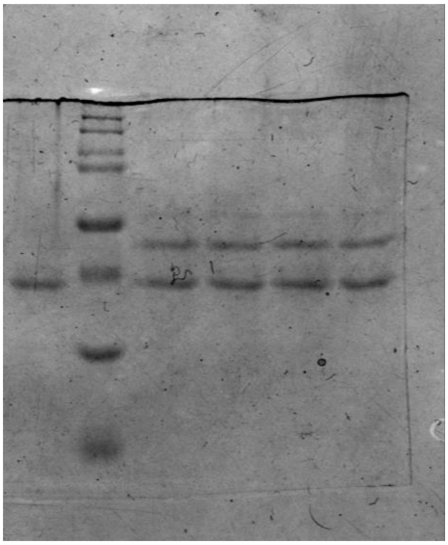

CBB

Figure S3

Presented figure

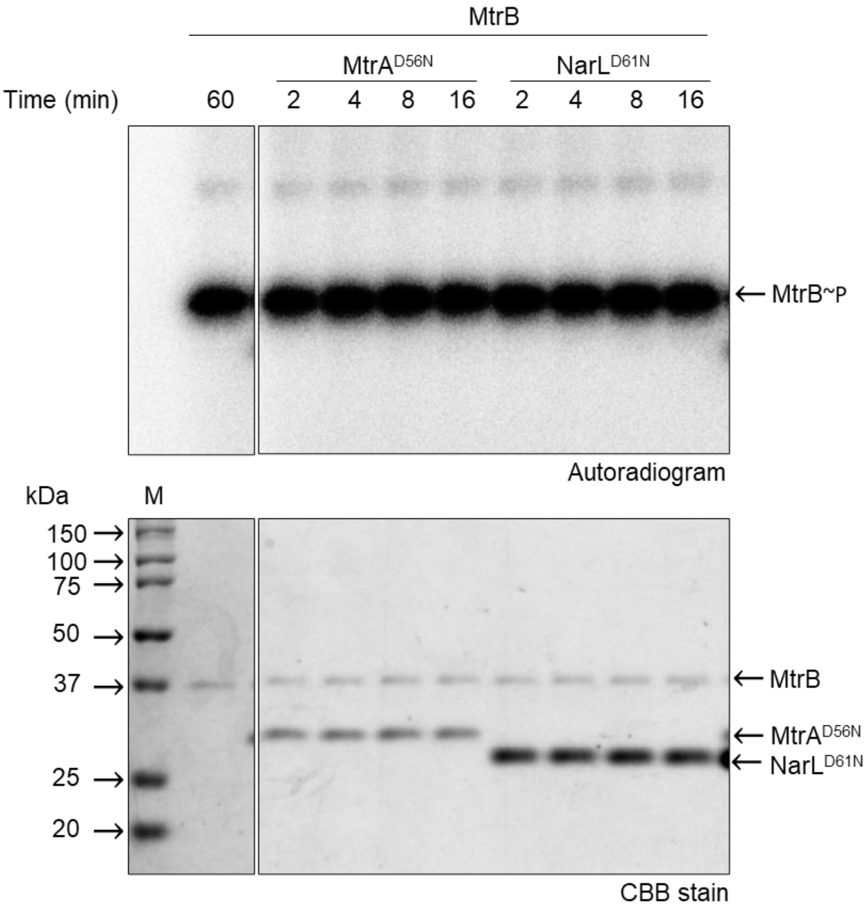

Unedited/uncropped image

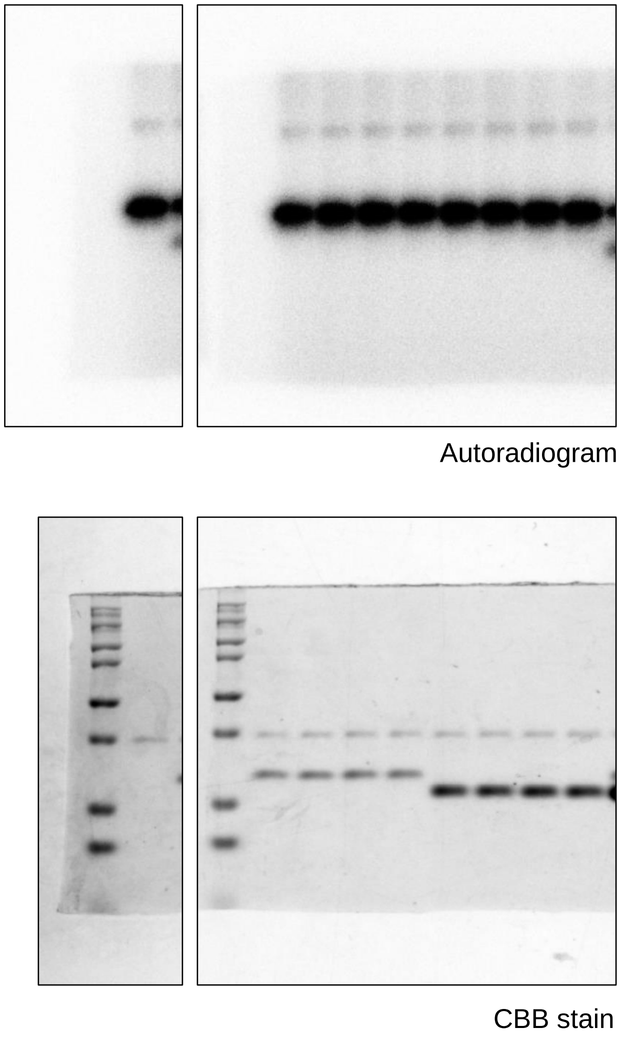

Figure S8A

Presented figure

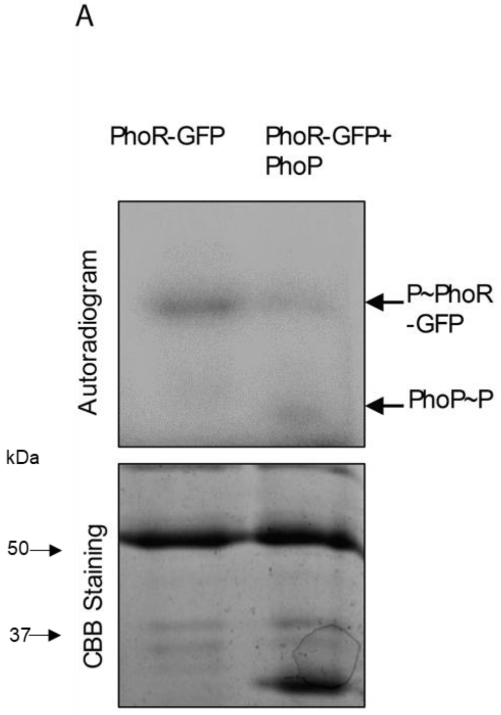

Unedited/uncropped image

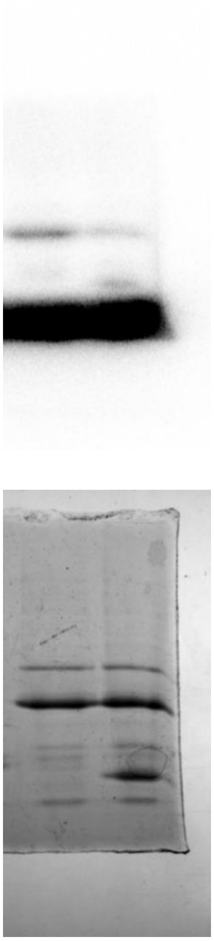

Figure S10B

Presented figure

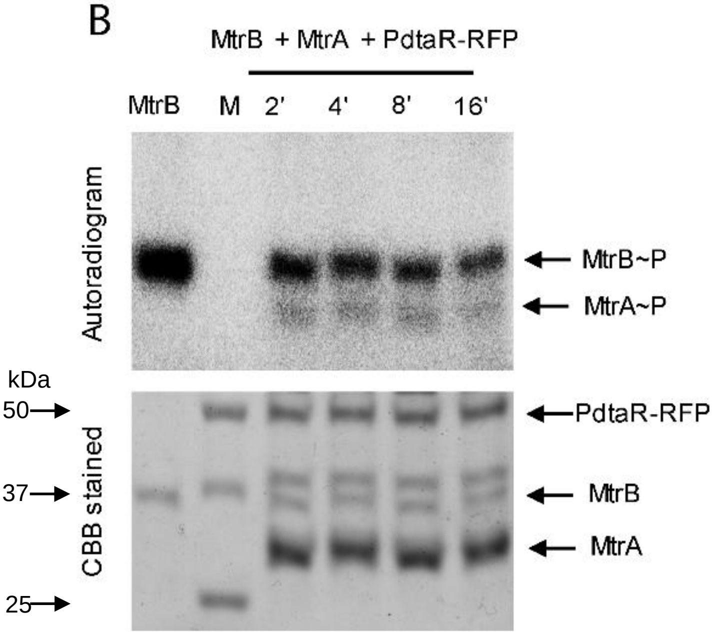

Unedited/uncropped image

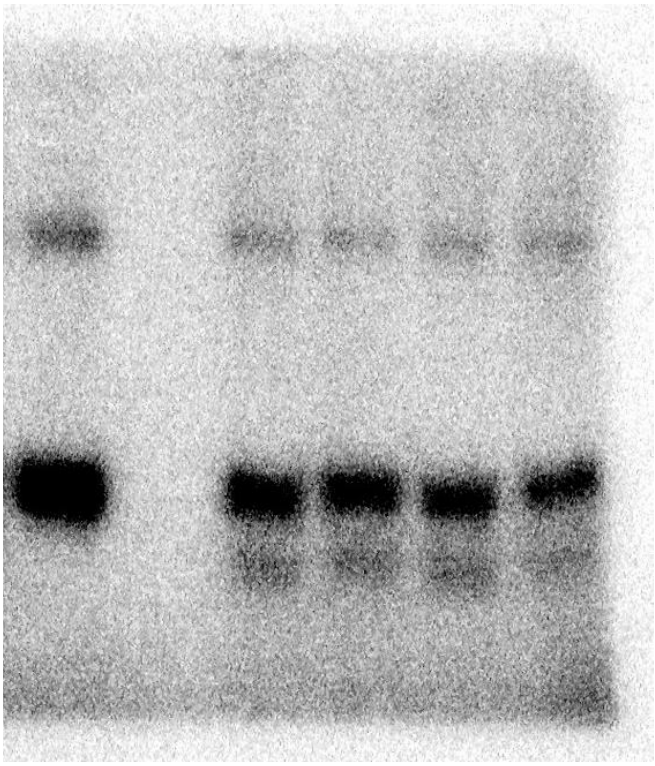

Autoradiogram

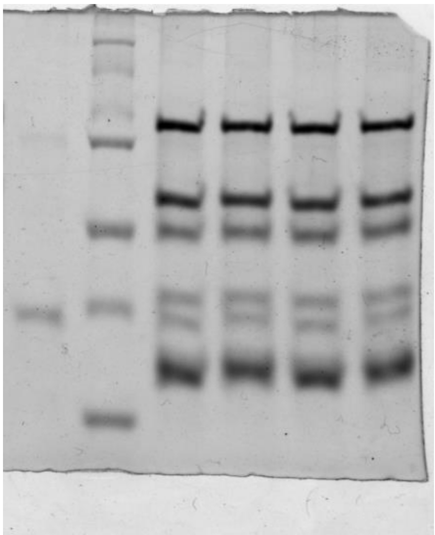

CBB

Figure S11

Presented figure

Histidine kinase	MtrB				
Response regulator		MtrA	MtrA D56N	NarL	NarL D61N
Time (min)	30 60	30 60	30 60	30 60	30 60

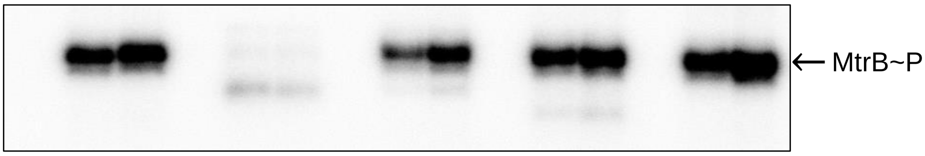

Autoradiogram

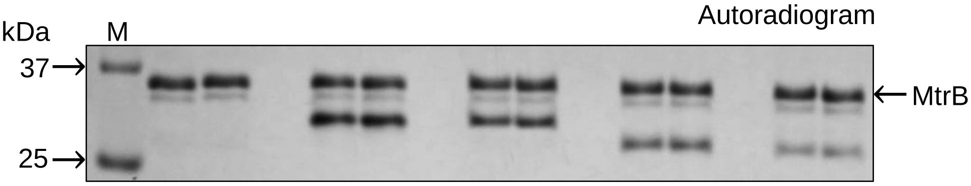

CBB stain

Unedited/uncropped image

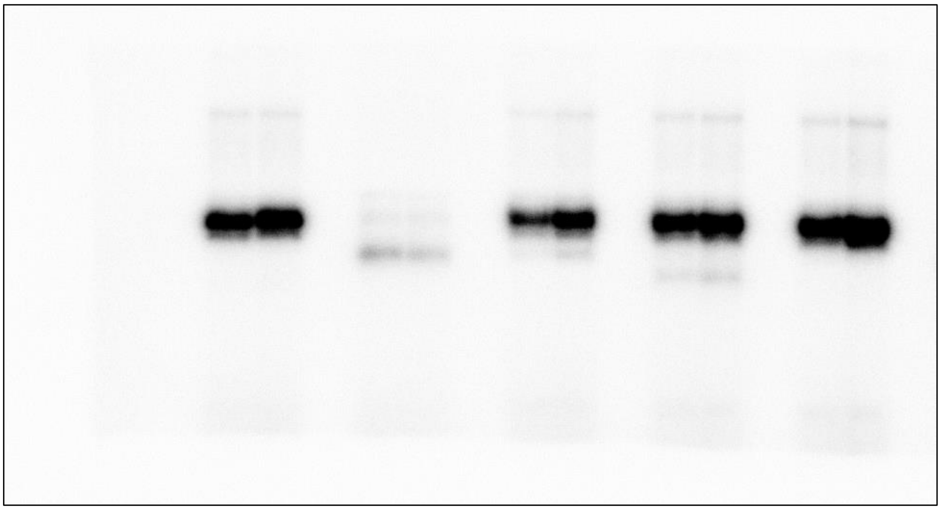

Autoradiogram

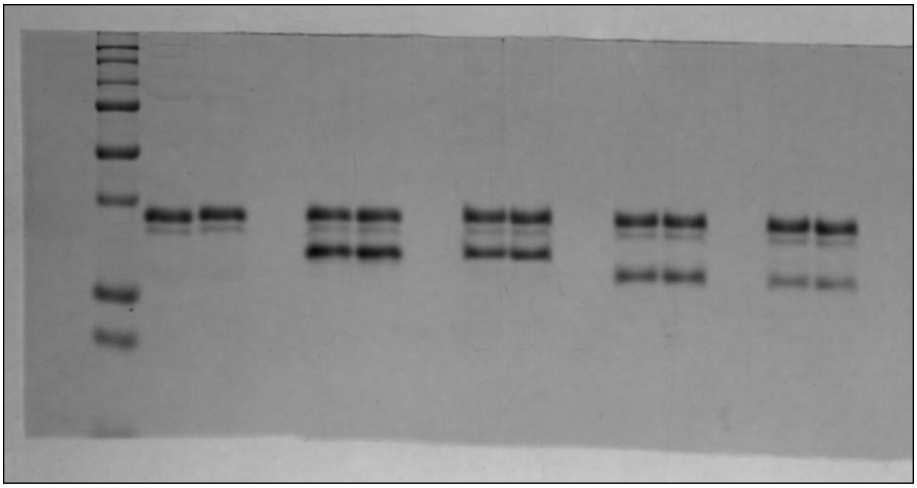

CBB stain
